# Supplementary material for: Role of Intracellular and Extracellular Annexin A1 in MIA PaCa-2 Spheroids Formation and Drug Sensitivity
Source: Cancers (Basel). 2022 Sep 29;14(19):4764. doi: 10.3390/cancers14194764 (PMC9563593; doi:10.3390/cancers14194764)
Supplement: Supplementary file 1 [file cancers-14-04764-s001.zip › Table S1.pdf]

Accession	Gene Symbol	Description	MW [kDa]	Fc ANXA1 KO *vs* WT	Log (2) Fc ANXA1 KO *vs* WT	P-Value ANXA1 KO *vs* WT	- Log (10) P-Value ANXA1 KO *vs* WT	Gene Ontology (biological process)	Gene Ontology (GO)
P03956	MMP1	Interstitial collagenase OS	54	100.00	6.64	1.91E-16	15.72	cellular response to UV-A [GO:0071492]; collagen catabolic process [GO:0030574]; extracellular matrix disassembly [GO:0022617]; extracellular matrix organization [GO:0030198]; positive regulation of protein-containing complex assembly [GO:0031334]; proteolysis [GO:0006508]	extracellular matrix [GO:0031012]; extracellular region [GO:0005576]; endopeptidase activity [GO:0004175]; metalloendopeptidase activity [GO:0004222]; peptidase activity [GO:0008233]; serine-type endopeptidase activity [GO:0004252]; zinc ion binding [GO:0008270]; cellular response to UV-A [GO:0071492]; collagen catabolic process [GO:0030574]; extracellular matrix disassembly [GO:0022617]; extracellular matrix organization [GO:0030198]; positive regulation of protein-containing complex assembly [GO:0031334]; proteolysis [GO:0006508]
P01893	HLA-H	Putative HLA class I histocompatibility antigen, alpha chain H OS	40.9	100.00	6.64	1.91E-16	15.72	antigen processing and presentation of endogenous peptide antigen via MHC class Ib [GO:0002476]; antigen processing and presentation of peptide antigen via MHC class I [GO:0002474]; immune response [GO:0006955]	azurophil granule membrane [GO:0035577]; cell surface [GO:0009986]; early endosome membrane [GO:0031901]; ER to Golgi transport vesicle membrane [GO:0012507]; external side of plasma membrane [GO:0009897]; extracellular space [GO:0005615]; Golgi membrane [GO:0000139]; integral component of lumenal side of endoplasmic reticulum membrane [GO:0071556]; integral component of plasma membrane [GO:0005887]; MHC class I protein complex [GO:0042612]; phagocytic vesicle membrane [GO:0030670]; plasma membrane [GO:0005886]; recycling endosome membrane [GO:0055038]; beta-2-microglobulin binding [GO:0030881]; antigen processing and presentation of endogenous peptide antigen via MHC class Ib [GO:0002476]; antigen processing and presentation of peptide antigen via MHC class I [GO:0002474]; immune response [GO:0006955]

P61020	RAB5B	Ras-related protein Rab-5B OS	23.7	100.00	6.64	1.91E-16	15.72	antigen processing and presentation [GO:0019882]; endocytosis [GO:0006897]; endosome organization [GO:0007032]; intracellular protein transport [GO:0006886]; plasma membrane to endosome transport [GO:0048227]; regulation of endocytosis [GO:0030100]	anchored component of synaptic vesicle membrane [GO:0098993]; early endosome [GO:0005769]; early endosome membrane [GO:0031901]; endocytic vesicle [GO:0030139]; endosome [GO:0005768]; extracellular exosome [GO:0070062]; intracellular membrane-bounded organelle [GO:0043231]; melanosome [GO:0042470]; membrane [GO:0016020]; plasma membrane [GO:0005886]; secretory granule membrane [GO:0030667]; G protein activity [GO:0003925]; GDP binding [GO:0019003]; GTP binding [GO:0005525]; GTP-dependent protein binding [GO:0030742]; GTPase activity [GO:0003924]; antigen processing and presentation [GO:0019882]; endocytosis [GO:0006897]; endosome organization [GO:0007032]; intracellular protein transport [GO:0006886]; plasma membrane to endosome transport [GO:0048227]; regulation of endocytosis [GO:0030100]
Q9BTE7	DCUN1D5	DCN1-like protein 5 OS	27.5	100.00	6.64	1.91E-16	15.72	cellular response to DNA damage stimulus [GO:0006974]; positive regulation of protein neddylation [GO:2000436]; positive regulation of ubiquitin-protein transferase activity [GO:0051443]; protein neddylation [GO:0045116]; regulation of cell growth [GO:0001558]; regulation of protein neddylation [GO:2000434]	cytoplasm [GO:0005737]; nucleus [GO:0005634]; spindle [GO:0005819]; ubiquitin ligase complex [GO:0000151]; cullin family protein binding [GO:0097602]; ubiquitin conjugating enzyme binding [GO:0031624]; ubiquitin-like protein binding [GO:0032182]; cellular response to DNA damage stimulus [GO:0006974]; positive regulation of protein neddylation [GO:2000436]; positive regulation of ubiquitin-protein transferase activity [GO:0051443]; protein neddylation [GO:0045116]; regulation of cell growth [GO:0001558]; regulation of protein neddylation [GO:2000434]
Q13410	BTN1A1	Butyrophilin subfamily 1 member A1 OS	58.9	100.00	6.64	1.91E-16	15.72	regulation of cytokine production [GO:0001817]; T cell receptor signaling pathway [GO:0050852]	external side of plasma membrane [GO:0009897]; extracellular space [GO:0005615]; integral component of plasma membrane [GO:0005887]; plasma membrane [GO:0005886]; signaling receptor activity [GO:0038023]; signaling receptor binding [GO:0005102]; regulation of cytokine production [GO:0001817]; T cell receptor signaling pathway [GO:0050852]

Q9NXW9	ALKBH4	Alpha-ketoglutarate-dependent dioxygenase alkB homolog 4 OS	33.8	100.00	6.64	1.91E-16	15.72	actomyosin structure organization [GO:0031032]; chromatin organization [GO:0006325]; cleavage furrow ingression [GO:0036090]; DNA demethylation [GO:0080111]; oxidative demethylation [GO:0070989]; protein demethylation [GO:0006482]; regulation of chromatin organization [GO:1902275]	contractile ring [GO:0070938]; cytoplasm [GO:0005737]; midbody [GO:0030496]; nucleolus [GO:0005730]; 2-oxoglutarate-dependent dioxygenase activity [GO:0016706]; actin binding [GO:0003779]; demethylase activity [GO:0032451]; metal ion binding [GO:0046872]; oxidative DNA demethylase activity [GO:0035516]; oxidoreductase activity [GO:0016491]; actomyosin structure organization [GO:0031032]; chromatin organization [GO:0006325]; cleavage furrow ingression [GO:0036090]; DNA demethylation [GO:0080111]; oxidative demethylation [GO:0070989]; protein demethylation [GO:0006482]; regulation of chromatin organization [GO:1902275]
Q8WWK9	CKAP2	Cytoskeleton-associated protein 2 OS	76.9	100.00	6.64	1.91E-16	15.72	apoptotic process [GO:0006915]; mitotic cytokinesis [GO:0000281]; negative regulation of microtubule depolymerization [GO:0007026]; positive regulation of transcription by RNA polymerase II [GO:0045944]	centrosome [GO:0005813]; cytoplasm [GO:0005737]; microtubule [GO:0005874]; microtubule cytoskeleton [GO:0015630]; mitotic spindle [GO:0072686]; spindle pole [GO:0000922]; apoptotic process [GO:0006915]; mitotic cytokinesis [GO:0000281]; negative regulation of microtubule depolymerization [GO:0007026]; positive regulation of transcription by RNA polymerase II [GO:0045944]
O60447	EVI5	Ecotropic viral integration site 5 protein homolog OS	92.9	100.00	6.64	1.91E-16	15.72	activation of GTPase activity [GO:0090630]; cell cycle [GO:0007049]; cell division [GO:0051301]; positive regulation of GTPase activity [GO:0043547]; retrograde transport, endosome to Golgi [GO:0042147]	cytosol [GO:0005829]; microtubule organizing center [GO:0005815]; nucleus [GO:0005634]; spindle [GO:0005819]; GTPase activator activity [GO:0005096]; small GTPase binding [GO:0031267]; activation of GTPase activity [GO:0090630]; cell cycle [GO:0007049]; cell division [GO:0051301]; positive regulation of GTPase activity [GO:0043547]; retrograde transport, endosome to Golgi [GO:0042147]
P12883	MYH7	Myosin-7 OS	223	100.00	6.64	1.91E-16	15.72	adult heart development [GO:0007512]; ATP metabolic process [GO:0046034]; cardiac muscle contraction [GO:0060048]; cardiac muscle hypertrophy in response to stress [GO:0014898]; muscle contraction [GO:0006936]; muscle filament sliding [GO:0030049]; regulation of heart rate [GO:0002027]; regulation of slow-twitch	muscle myosin complex [GO:0005859]; myofibril [GO:0030016]; myosin complex [GO:0016459]; myosin filament [GO:0032982]; myosin II complex [GO:0016460]; sarcomere [GO:0030017]; stress fiber [GO:0001725]; Z disc [GO:0030018]; actin filament binding [GO:0051015]; ATP binding [GO:0005524]; calmodulin binding [GO:0005516]; microfilament motor activity [GO:0000146]; adult heart

								skeletal muscle fiber contraction [GO:0031449]; regulation of the force of heart contraction [GO:0002026]; regulation of the force of skeletal muscle contraction [GO:0014728]; sarcomere organization [GO:0045214]; skeletal muscle contraction [GO:0003009]; striated muscle contraction [GO:0006941]; transition between fast and slow fiber [GO:0014883]; ventricular cardiac muscle tissue morphogenesis [GO:0055010]	development [GO:0007512]; ATP metabolic process [GO:0046034]; cardiac muscle contraction [GO:0060048]; cardiac muscle hypertrophy in response to stress [GO:0014898]; muscle contraction [GO:0006936]; muscle filament sliding [GO:0030049]; regulation of heart rate [GO:0002027]; regulation of slow-twitch skeletal muscle fiber contraction [GO:0031449]; regulation of the force of heart contraction [GO:0002026]; regulation of the force of skeletal muscle contraction [GO:0014728]; sarcomere organization [GO:0045214]; skeletal muscle contraction [GO:0003009]; striated muscle contraction [GO:0006941]; transition between fast and slow fiber [GO:0014883]; ventricular cardiac muscle tissue morphogenesis [GO:0055010]
Q14558	PRPSAP1	Phosphoribosyl pyrophosphate synthase-associated protein 1 OS	39.4	100.00	6.64	1.91E-16	15.72	5-phosphoribose 1-diphosphate biosynthetic process [GO:0006015]; nucleobase-containing compound metabolic process [GO:0006139]; purine nucleotide biosynthetic process [GO:0006164]	cytoplasm [GO:0005737]; ribose phosphate diphosphokinase complex [GO:0002189]; enzyme inhibitor activity [GO:0004857]; identical protein binding [GO:0042802]; magnesium ion binding [GO:0000287]; ribose phosphate diphosphokinase activity [GO:0004749]; 5-phosphoribose 1-diphosphate biosynthetic process [GO:0006015]; nucleobase-containing compound metabolic process [GO:0006139]; purine nucleotide biosynthetic process [GO:0006164]
Q9NZM5	NOP53	Ribosome biogenesis protein NOP53 OS	54.4	100.00	6.64	1.91E-16	15.72	cellular response to DNA damage stimulus [GO:0006974]; cellular response to hypoxia [GO:0071456]; DNA repair [GO:0006281]; mitotic G2 DNA damage checkpoint signaling [GO:0007095]; negative regulation of phosphatidylinositol 3-kinase signaling [GO:0014067]; negative regulation of proteasomal ubiquitin-dependent protein catabolic process [GO:0032435]; negative regulation of protein kinase B signaling [GO:0051898]; negative regulation of protein-containing complex assembly [GO:0031333]; negative regulation of signal transduction by p53 class mediator	cytosol [GO:0005829]; fibrillar center [GO:0001650]; intracellular membrane-bounded organelle [GO:0043231]; nucleolus [GO:0005730]; nucleoplasm [GO:0005654]; 5S rRNA binding [GO:0008097]; identical protein binding [GO:0042802]; p53 binding [GO:0002039]; RNA binding [GO:0003723]; cellular response to DNA damage stimulus [GO:0006974]; cellular response to hypoxia [GO:0071456]; DNA repair [GO:0006281]; mitotic G2 DNA damage checkpoint signaling [GO:0007095]; negative regulation of phosphatidylinositol 3-kinase signaling [GO:0014067]; negative regulation of proteasomal ubiquitin-dependent protein catabolic process [GO:0032435]; negative regulation of protein kinase

								[GO:1901797]; negative regulation of transcription by RNA polymerase II [GO:0000122]; negative regulation of transcription of nucleolar large rRNA by RNA polymerase I [GO:1901837]; positive regulation of proteasomal ubiquitin-dependent protein catabolic process [GO:0032436]; positive regulation of protein K63-linked deubiquitination [GO:1903006]; protein localization to nucleolus [GO:1902570]; protein localization to nucleoplasm [GO:1990173]; protein stabilization [GO:0050821]; regulation of aerobic respiration [GO:1903715]; regulation of apoptotic process [GO:0042981]; regulation of cell cycle [GO:0051726]; regulation of protein phosphorylation [GO:0001932]; regulation of RIG-I signaling pathway [GO:0039535]; regulation of signal transduction by p53 class mediator [GO:1901796]; ribosomal large subunit assembly [GO:0000027]; rRNA processing [GO:0006364]	B signaling [GO:0051898]; negative regulation of protein-containing complex assembly [GO:0031333]; negative regulation of signal transduction by p53 class mediator [GO:1901797]; negative regulation of transcription by RNA polymerase II [GO:0000122]; negative regulation of transcription of nucleolar large rRNA by RNA polymerase I [GO:1901837]; positive regulation of proteasomal ubiquitin-dependent protein catabolic process [GO:0032436]; positive regulation of protein K63-linked deubiquitination [GO:1903006]; protein localization to nucleolus [GO:1902570]; protein localization to nucleoplasm [GO:1990173]; protein stabilization [GO:0050821]; regulation of aerobic respiration [GO:1903715]; regulation of apoptotic process [GO:0042981]; regulation of cell cycle [GO:0051726]; regulation of protein phosphorylation [GO:0001932]; regulation of RIG-I signaling pathway [GO:0039535]; regulation of signal transduction by p53 class mediator [GO:1901796]; ribosomal large subunit assembly [GO:0000027]; rRNA processing [GO:0006364]
Q8NBP0	TTC13	Tetratricopeptide repeat protein 13 OS	96.8	100.00	6.64	1.91E-16	15.72		
O00471	EXOC5	Exocyst complex component 5 OS	81.8	67.04	6.07	1.01E-12	12.00	epithelial cell apoptotic process [GO:1904019]; establishment of planar polarity [GO:0001736]; exocytosis [GO:0006887]; Golgi to plasma membrane transport [GO:0006893]; membrane fission [GO:0090148]; mitotic cytokinesis [GO:0000281]; non-motile cilium assembly [GO:1905515]; post-Golgi vesicle-mediated transport [GO:0006892]; protein localization to plasma membrane [GO:0072659]; protein transport [GO:0015031]; vesicle docking involved in exocytosis [GO:0006904]; vesicle tethering involved in exocytosis [GO:0090522]	cytoplasm [GO:0005737]; cytosol [GO:0005829]; exocyst [GO:0000145]; midbody [GO:0030496]; protein N-terminus binding [GO:0047485]; small GTPase binding [GO:0031267]; epithelial cell apoptotic process [GO:1904019]; establishment of planar polarity [GO:0001736]; exocytosis [GO:0006887]; Golgi to plasma membrane transport [GO:0006893]; membrane fission [GO:0090148]; mitotic cytokinesis [GO:0000281]; non-motile cilium assembly [GO:1905515]; post-Golgi vesicle-mediated transport [GO:0006892]; protein localization to plasma membrane [GO:0072659]; protein transport [GO:0015031]; vesicle docking involved in exocytosis

									[GO:0006904]; vesicle tethering involved in exocytosis [GO:0090522]
P02452	COL1A1	Collagen alpha-1(I) chain OS	138.9	60.89	5.93	1.50E-11	10.82	blood vessel development [GO:0001568]; bone trabecula formation [GO:0060346]; cartilage development involved in endochondral bone morphogenesis [GO:0060351]; cellular response to amino acid stimulus [GO:0071230]; cellular response to epidermal growth factor stimulus [GO:0071364]; cellular response to fibroblast growth factor stimulus [GO:0044344]; cellular response to fluoride [GO:1902618]; cellular response to mechanical stimulus [GO:0071260]; cellular response to retinoic acid [GO:0071300]; cellular response to transforming growth factor beta stimulus [GO:0071560]; cellular response to tumor necrosis factor [GO:0071356]; cellular response to vitamin E [GO:0071306]; collagen biosynthetic process [GO:0032964]; collagen fibril organization [GO:0030199]; collagen-activated tyrosine kinase receptor signaling pathway [GO:0038063]; embryonic skeletal system development [GO:0048706]; endochondral ossification [GO:0001958]; extracellular matrix organization [GO:0030198]; face morphogenesis [GO:0060325]; intramembranous ossification [GO:0001957]; negative regulation of cell-substrate adhesion [GO:0010812]; ossification [GO:0001503]; osteoblast differentiation [GO:0001649]; positive regulation of canonical Wnt signaling pathway [GO:0090263]; positive regulation of cell migration [GO:0030335]; positive regulation of epithelial to mesenchymal transition [GO:0010718]; positive regulation of transcription, DNA-templated [GO:0045893]; protein localization to nucleus	collagen type I trimer [GO:0005584]; collagen-containing extracellular matrix [GO:0062023]; cytoplasm [GO:0005737]; endoplasmic reticulum lumen [GO:0005788]; extracellular matrix [GO:0031012]; extracellular region [GO:0005576]; extracellular space [GO:0005615]; Golgi apparatus [GO:0005794]; secretory granule [GO:0030141]; extracellular matrix structural constituent [GO:0005201]; extracellular matrix structural constituent conferring tensile strength [GO:0030020]; identical protein binding [GO:0042802]; metal ion binding [GO:0046872]; platelet-derived growth factor binding [GO:0048407]; protease binding [GO:0002020]; blood vessel development [GO:0001568]; bone trabecula formation [GO:0060346]; cartilage development involved in endochondral bone morphogenesis [GO:0060351]; cellular response to amino acid stimulus [GO:0071230]; cellular response to epidermal growth factor stimulus [GO:0071364]; cellular response to fibroblast growth factor stimulus [GO:0044344]; cellular response to fluoride [GO:1902618]; cellular response to mechanical stimulus [GO:0071260]; cellular response to retinoic acid [GO:0071300]; cellular response to transforming growth factor beta stimulus [GO:0071560]; cellular response to tumor necrosis factor [GO:0071356]; cellular response to vitamin E [GO:0071306]; collagen biosynthetic process [GO:0032964]; collagen fibril organization [GO:0030199]; collagen-activated tyrosine kinase receptor signaling pathway [GO:0038063]; embryonic skeletal system development [GO:0048706]; endochondral ossification [GO:0001958]; extracellular matrix organization [GO:0030198]; face morphogenesis [GO:0060325]; intramembranous ossification [GO:0001957]; negative regulation of cell-substrate

								[GO:0034504]; protein transport [GO:0015031]; response to cAMP [GO:0051591]; response to corticosteroid [GO:0031960]; response to estradiol [GO:0032355]; response to hydrogen peroxide [GO:0042542]; response to hyperoxia [GO:0055093]; response to mechanical stimulus [GO:0009612]; response to peptide hormone [GO:0043434]; response to xenobiotic stimulus [GO:0009410]; sensory perception of sound [GO:0007605]; skeletal system development [GO:0001501]; skin development [GO:0043588]; skin morphogenesis [GO:0043589]; tooth eruption [GO:0044691]; tooth mineralization [GO:0034505]; visual perception [GO:0007601]; wound healing [GO:0042060]	adhesion [GO:0010812]; ossification [GO:0001503]; osteoblast differentiation [GO:0001649]; positive regulation of canonical Wnt signaling pathway [GO:0090263]; positive regulation of cell migration [GO:0030335]; positive regulation of epithelial to mesenchymal transition [GO:0010718]; positive regulation of transcription, DNA-templated [GO:0045893]; protein localization to nucleus [GO:0034504]; protein transport [GO:0015031]; response to cAMP [GO:0051591]; response to corticosteroid [GO:0031960]; response to estradiol [GO:0032355]; response to hydrogen peroxide [GO:0042542]; response to hyperoxia [GO:0055093]; response to mechanical stimulus [GO:0009612]; response to peptide hormone [GO:0043434]; response to xenobiotic stimulus [GO:0009410]; sensory perception of sound [GO:0007605]; skeletal system development [GO:0001501]; skin development [GO:0043588]; skin morphogenesis [GO:0043589]; tooth eruption [GO:0044691]; tooth mineralization [GO:0034505]; visual perception [GO:0007601]; wound healing [GO:0042060]
Q8WZ42	TTN	Titin OS	3813.7	31.82	4.99	3.09E-08	7.51	cardiac muscle cell development [GO:0055013]; cardiac muscle contraction [GO:0060048]; cardiac muscle hypertrophy [GO:0003300]; cardiac muscle tissue morphogenesis [GO:0055008]; cardiac myofibril assembly [GO:0055003]; detection of muscle stretch [GO:0035995]; mitotic chromosome condensation [GO:0007076]; muscle contraction [GO:0006936]; positive regulation of gene expression [GO:0010628]; positive regulation of protein secretion [GO:0050714]; protein kinase A signaling [GO:0010737]; regulation of catalytic activity [GO:0050790]; regulation of protein kinase activity [GO:0045859]; response to calcium ion [GO:0051592]; sarcomere organization [GO:0045214]; sarcomerogenesis [GO:0048769]; skeletal muscle myosin thick filament assembly [GO:0030241]; skeletal	condensed nuclear chromosome [GO:0000794]; cytosol [GO:0005829]; extracellular exosome [GO:0070062]; extracellular region [GO:0005576]; I band [GO:0031674]; M band [GO:0031430]; striated muscle thin filament [GO:0005865]; titin-telethonin complex [GO:1990733]; Z disc [GO:0030018]; actin filament binding [GO:0051015]; actinin binding [GO:0042805]; ATP binding [GO:0005524]; calcium ion binding [GO:0005509]; calmodulin binding [GO:0005516]; enzyme binding [GO:0019899]; identical protein binding [GO:0042802]; muscle alpha-actinin binding [GO:0051371]; protease binding [GO:0002020]; protein kinase binding [GO:0019901]; protein self-association [GO:0043621]; protein serine kinase activity [GO:0106310]; protein serine/threonine kinase activity [GO:0004674]; protein tyrosine kinase activity [GO:0004713]; structural constituent of muscle [GO:0008307]; structural molecule activity

								muscle thin filament assembly [GO:0030240]; striated muscle contraction [GO:0006941]	conferring elasticity [GO:0097493]; telethonin binding [GO:0031433]; cardiac muscle cell development [GO:0055013]; cardiac muscle contraction [GO:0060048]; cardiac muscle hypertrophy [GO:0003300]; cardiac muscle tissue morphogenesis [GO:0055008]; cardiac myofibril assembly [GO:0055003]; detection of muscle stretch [GO:0035995]; mitotic chromosome condensation [GO:0007076]; muscle contraction [GO:0006936]; positive regulation of gene expression [GO:0010628]; positive regulation of protein secretion [GO:0050714]; protein kinase A signaling [GO:0010737]; regulation of catalytic activity [GO:0050790]; regulation of protein kinase activity [GO:0045859]; response to calcium ion [GO:0051592]; sarcomere organization [GO:0045214]; sarcomerogenesis [GO:0048769]; skeletal muscle myosin thick filament assembly [GO:0030241]; skeletal muscle thin filament assembly [GO:0030240]; striated muscle contraction [GO:0006941]
O75879	GATB	Glutamyl-tRNA(Gln) amidotransferase subunit B, mitochondrial OS	61.8	26.94	4.75	6.37E-06	5.20	glutaminyl-tRNAGln biosynthesis via transamidation [GO:0070681]; mitochondrial translation [GO:0032543]	glutamyl-tRNA(Gln) amidotransferase complex [GO:0030956]; mitochondrion [GO:0005739]; ATP binding [GO:0005524]; glutaminyl-tRNA synthase (glutamine-hydrolyzing) activity [GO:0050567]; glutaminyl-tRNAGln biosynthesis via transamidation [GO:0070681]; mitochondrial translation [GO:0032543]
P29322	EPHA8	Ephrin type-A receptor 8 OS	110.9	25.24	4.66	2.39E-11	10.62	axon guidance [GO:0007411]; cell adhesion [GO:0007155]; cellular response to follicle-stimulating hormone stimulus [GO:0071372]; ephrin receptor signaling pathway [GO:0048013]; neuron projection development [GO:0031175]; neuron remodeling [GO:0016322]; positive regulation of kinase activity [GO:0033674]; positive regulation of MAPK cascade [GO:0043410]; positive regulation of phosphatidylinositol 3-kinase activity [GO:0043552]; protein autophosphorylation	early endosome membrane [GO:0031901]; integral component of plasma membrane [GO:0005887]; neuron projection [GO:0043005]; plasma membrane [GO:0005886]; receptor complex [GO:0043235]; ATP binding [GO:0005524]; GPI-linked ephrin receptor activity [GO:0005004]; growth factor binding [GO:0019838]; transmembrane receptor protein tyrosine kinase activity [GO:0004714]; transmembrane-ephrin receptor activity [GO:0005005]; axon guidance [GO:0007411]; cell adhesion [GO:0007155]; cellular response to follicle-stimulating hormone stimulus [GO:0071372]; ephrin

								[GO:0046777]; regulation of cell adhesion [GO:0030155]; regulation of cell adhesion mediated by integrin [GO:0033628]; substrate-dependent cell migration [GO:0006929]; transmembrane receptor protein tyrosine kinase signaling pathway [GO:0007169]	receptor signaling pathway [GO:0048013]; neuron projection development [GO:0031175]; neuron remodeling [GO:0016322]; positive regulation of kinase activity [GO:0033674]; positive regulation of MAPK cascade [GO:0043410]; positive regulation of phosphatidylinositol 3-kinase activity [GO:0043552]; protein autophosphorylation [GO:0046777]; regulation of cell adhesion [GO:0030155]; regulation of cell adhesion mediated by integrin [GO:0033628]; substrate-dependent cell migration [GO:0006929]; transmembrane receptor protein tyrosine kinase signaling pathway [GO:0007169]
O15127	SCAMP2	Secretory carrier-associated membrane protein 2 OS	36.6	24.23	4.60	1.36E-05	4.87	post-Golgi vesicle-mediated transport [GO:0006892]; protein transport [GO:0015031]	extracellular exosome [GO:0070062]; Golgi apparatus [GO:0005794]; integral component of membrane [GO:0016021]; intracellular membrane-bounded organelle [GO:0043231]; recycling endosome membrane [GO:0055038]; trans-Golgi network membrane [GO:0032588]; post-Golgi vesicle-mediated transport [GO:0006892]; protein transport [GO:0015031]
Q09327	MGAT3	Beta-1,4-mannosyl-glycoprotein 4-beta-N-acetylglucosaminyltransferase OS	61.3	23.89	4.58	6.92E-07	6.16	amyloid-beta metabolic process [GO:0050435]; cellular response to oxidative stress [GO:0034599]; cognition [GO:0050890]; N-acetylglucosamine metabolic process [GO:0006044]; negative regulation of lysosomal protein catabolic process [GO:1905166]; positive regulation of protein localization to early endosome [GO:1902966]; protein localization [GO:0008104]; protein N-linked glycosylation [GO:0006487]; regulation of cell migration [GO:0030334]	Golgi membrane [GO:0000139]; integral component of membrane [GO:0016021]; beta-1,4-mannosylglycoprotein 4-beta-N-acetylglucosaminyltransferase activity [GO:0003830]; glycosyltransferase activity [GO:0016757]; amyloid-beta metabolic process [GO:0050435]; cellular response to oxidative stress [GO:0034599]; cognition [GO:0050890]; N-acetylglucosamine metabolic process [GO:0006044]; negative regulation of lysosomal protein catabolic process [GO:1905166]; positive regulation of protein localization to early endosome [GO:1902966]; protein localization [GO:0008104]; protein N-linked glycosylation [GO:0006487]; regulation of cell migration [GO:0030334]

Q6IQ22	RAB12	Ras-related protein Rab-12 OS	27.2	23.07	4.53	1.24E-07	6.91	autophagy [GO:0006914]; cellular response to insulin stimulus [GO:0032869]; endosome to lysosome transport [GO:0008333]; protein catabolic process [GO:0030163]; protein localization to plasma membrane [GO:0072659]; protein secretion [GO:0009306]; Rab protein signal transduction [GO:0032482]; regulation of exocytosis [GO:0017157]; vesicle docking involved in exocytosis [GO:0006904]	autophagosome [GO:0005776]; cytosol [GO:0005829]; endosome [GO:0005768]; Golgi apparatus [GO:0005794]; Golgi membrane [GO:0000139]; insulin-responsive compartment [GO:0032593]; intracellular membrane-bounded organelle [GO:0043231]; lysosomal membrane [GO:0005765]; lysosome [GO:0005764]; plasma membrane [GO:0005886]; recycling endosome [GO:0055037]; recycling endosome membrane [GO:0055038]; synaptic vesicle [GO:0008021]; GDP binding [GO:0019003]; GTP binding [GO:0005525]; GTPase activity [GO:0003924]; autophagy [GO:0006914]; cellular response to insulin stimulus [GO:0032869]; endosome to lysosome transport [GO:0008333]; protein catabolic process [GO:0030163]; protein localization to plasma membrane [GO:0072659]; protein secretion [GO:0009306]; Rab protein signal transduction [GO:0032482]; regulation of exocytosis [GO:0017157]; vesicle docking involved in exocytosis [GO:0006904]
Q99996	AKAP9	A-kinase anchor protein 9 OS	452.7	21.38	4.42	2.45E-05	4.61	cellular response to cAMP [GO:0071320]; chemical synaptic transmission [GO:0007268]; maintenance of centrosome location [GO:0051661]; microtubule nucleation [GO:0007020]; negative regulation of adenylate cyclase activity [GO:0007194]; positive regulation of microtubule polymerization [GO:0031116]; positive regulation of peptidyl-serine phosphorylation [GO:0033138]; positive regulation of potassium ion transmembrane transporter activity [GO:1901018]; protein-containing complex localization [GO:0031503]; regulation of cardiac muscle cell action potential involved in regulation of contraction [GO:0098909]; regulation of Golgi organization [GO:1903358]; regulation of heart rate by cardiac conduction [GO:0086091]; regulation of membrane repolarization [GO:0060306]; regulation of	centrosome [GO:0005813]; cis-Golgi network [GO:0005801]; cytoskeleton [GO:0005856]; cytosol [GO:0005829]; dendritic branch [GO:0044307]; extrinsic component of postsynaptic density membrane [GO:0099147]; glutamatergic synapse [GO:0098978]; Golgi apparatus [GO:0005794]; Golgi stack [GO:0005795]; intracellular membrane-bounded organelle [GO:0043231]; neuronal cell body [GO:0043025]; potassium channel complex [GO:0034705]; synaptic membrane [GO:0097060]; voltage-gated potassium channel complex [GO:0008076]; DNA binding [GO:0003677]; molecular adaptor activity [GO:0060090]; potassium channel regulator activity [GO:0015459]; protein kinase A regulatory subunit binding [GO:0034237]; signaling receptor binding [GO:0005102]; transmembrane transporter binding [GO:0044325]; cellular response to cAMP [GO:0071320]; chemical synaptic transmission [GO:0007268]; maintenance of centrosome location [GO:0051661]; microtubule

								postsynaptic neurotransmitter receptor activity [GO:0098962]; regulation of ventricular cardiac muscle cell membrane repolarization [GO:0060307]; response to electrical stimulus [GO:0051602]; signal transduction [GO:0007165]	nucleation [GO:0007020]; negative regulation of adenylate cyclase activity [GO:0007194]; positive regulation of microtubule polymerization [GO:0031116]; positive regulation of peptidyl-serine phosphorylation [GO:0033138]; positive regulation of potassium ion transmembrane transporter activity [GO:1901018]; protein-containing complex localization [GO:0031503]; regulation of cardiac muscle cell action potential involved in regulation of contraction [GO:0098909]; regulation of Golgi organization [GO:1903358]; regulation of heart rate by cardiac conduction [GO:0086091]; regulation of membrane repolarization [GO:0060306]; regulation of postsynaptic neurotransmitter receptor activity [GO:0098962]; regulation of ventricular cardiac muscle cell membrane repolarization [GO:0060307]; response to electrical stimulus [GO:0051602]; signal transduction [GO:0007165]
P07197	NEFM	Neurofilament medium polypeptide OS	102.4	20.36	4.35	4.12E-10	9.38	intermediate filament organization [GO:0045109]; neurofilament bundle assembly [GO:0033693]	axon [GO:0030424]; cytoplasm [GO:0005737]; intermediate filament [GO:0005882]; intermediate filament cytoskeleton [GO:0045111]; neurofibrillary tangle [GO:0097418]; neurofilament [GO:0005883]; postsynaptic intermediate filament cytoskeleton [GO:0099160]; microtubule binding [GO:0008017]; structural constituent of cytoskeleton [GO:0005200]; intermediate filament organization [GO:0045109]; neurofilament bundle assembly [GO:0033693]
Q8IWY8	ZSCAN29	Zinc finger and SCAN domain-containing protein 29 OS	96.7	19.69	4.30	6.31E-10	9.20	regulation of transcription by RNA polymerase II [GO:0006357]	nucleus [GO:0005634]; DNA-binding transcription factor activity, RNA polymerase II-specific [GO:0000981]; metal ion binding [GO:0046872]; RNA polymerase II cis-regulatory region sequence-specific DNA binding [GO:0000978]; sequence-specific double-stranded DNA binding [GO:1990837]; regulation of transcription by RNA polymerase II [GO:0006357]
Q8IY47	KBTBD2	Kelch repeat and BTB domain-containing protein 2 OS	71.3	17.58	4.14	2.58E-09	8.59	gene expression [GO:0010467]; glucose metabolic process [GO:0006006]; lipid metabolic process [GO:0006629]; phosphatidylinositol 3-kinase signaling	gene expression [GO:0010467]; glucose metabolic process [GO:0006006]; lipid metabolic process [GO:0006629]; phosphatidylinositol 3-kinase

								[GO:0014065]; response to insulin [GO:0032868]	signaling [GO:0014065]; response to insulin [GO:0032868]
P35749	MYH11	Myosin-11 OS	227.2	16.94	4.08	4.07E-09	8.39	actomyosin structure organization [GO:0031032]; cardiac muscle cell development [GO:0055013]; elastic fiber assembly [GO:0048251]; skeletal muscle myosin thick filament assembly [GO:0030241]; smooth muscle contraction [GO:0006939]	cytosol [GO:0005829]; extracellular exosome [GO:0070062]; melanosome [GO:0042470]; muscle myosin complex [GO:0005859]; myosin filament [GO:0032982]; myosin II complex [GO:0016460]; actin filament binding [GO:0051015]; ATP binding [GO:0005524]; calmodulin binding [GO:0005516]; microfilament motor activity [GO:0000146]; structural constituent of muscle [GO:0008307]; actomyosin structure organization [GO:0031032]; cardiac muscle cell development [GO:0055013]; elastic fiber assembly [GO:0048251]; skeletal muscle myosin thick filament assembly [GO:0030241]; smooth muscle contraction [GO:0006939]
Q8IW75	SERPINA12	Serpin A12 OS	47.1	16.48	4.04	4.56E-06	5.34	gluconeogenesis [GO:0006094]; lipid biosynthetic process [GO:0008610]; negative regulation of endopeptidase activity [GO:0010951]; negative regulation of gluconeogenesis [GO:0045721]; negative regulation of lipid biosynthetic process [GO:0051055]; phosphatidylinositol 3-kinase signaling [GO:0014065]; positive regulation of insulin receptor signaling pathway [GO:0046628]; positive regulation of phosphatidylinositol 3-kinase signaling [GO:0014068]; regulation of cholesterol metabolic process [GO:0090181]; regulation of triglyceride metabolic process [GO:0090207]	extracellular space [GO:0005615]; plasma membrane [GO:0005886]; serine-type endopeptidase inhibitor activity [GO:0004867]; gluconeogenesis [GO:0006094]; lipid biosynthetic process [GO:0008610]; negative regulation of endopeptidase activity [GO:0010951]; negative regulation of gluconeogenesis [GO:0045721]; negative regulation of lipid biosynthetic process [GO:0051055]; phosphatidylinositol 3-kinase signaling [GO:0014065]; positive regulation of insulin receptor signaling pathway [GO:0046628]; positive regulation of phosphatidylinositol 3-kinase signaling [GO:0014068]; regulation of cholesterol metabolic process [GO:0090181]; regulation of triglyceride metabolic process [GO:0090207]
Q7Z6R9	TFAP2D	Transcription factor AP-2-delta OS	49.5	16.00	4.00	2.14E-04	3.67	anatomical structure development [GO:0048856]; inferior colliculus development [GO:0061379]; negative regulation of neuron apoptotic process [GO:0043524]; positive regulation of transcription by RNA polymerase II [GO:0045944]; regulation of cell population proliferation [GO:0042127]; regulation of transcription by RNA polymerase II	chromatin [GO:0000785]; nucleus [GO:0005634]; transcription regulator complex [GO:0005667]; DNA-binding transcription factor activity, RNA polymerase II-specific [GO:0000981]; RNA polymerase II transcription regulatory region sequence-specific DNA binding [GO:0000977]; anatomical structure development [GO:0048856]; inferior colliculus development [GO:0061379]; negative regulation of neuron apoptotic process [GO:0043524]; positive

								[GO:0006357]; transcription by RNA polymerase II [GO:0006366]	regulation of transcription by RNA polymerase II [GO:0045944]; regulation of cell population proliferation [GO:0042127]; regulation of transcription by RNA polymerase II [GO:0006357]; transcription by RNA polymerase II [GO:0006366]
Q15427	SF3B4	Splicing factor 3B subunit 4 OS	44.4	12.38	3.63	3.24E-05	4.49	mRNA processing [GO:0006397]; mRNA splicing, via spliceosome [GO:0000398]; positive regulation of mRNA splicing, via spliceosome [GO:0048026]; RNA splicing [GO:0008380]; RNA splicing, via transesterification reactions [GO:0000375]; U2-type prespliceosome assembly [GO:1903241]	nucleoplasm [GO:0005654]; nucleus [GO:0005634]; spliceosomal complex [GO:0005681]; U12-type spliceosomal complex [GO:0005689]; U2 snRNP [GO:0005686]; U2-type precatalytic spliceosome [GO:0071005]; U2-type spliceosomal complex [GO:0005684]; RNA binding [GO:0003723]; splicing factor binding [GO:1990935]; mRNA processing [GO:0006397]; mRNA splicing, via spliceosome [GO:0000398]; positive regulation of mRNA splicing, via spliceosome [GO:0048026]; RNA splicing [GO:0008380]; RNA splicing, via transesterification reactions [GO:0000375]; U2-type prespliceosome assembly [GO:1903241]
Q8NCH0	CHST14	Carbohydrate sulfotransferase 14 OS	43	12.22	3.61	1.09E-04	3.96	carbohydrate biosynthetic process [GO:0016051]; dermatan sulfate biosynthetic process [GO:0030208]; dermatan sulfate proteoglycan metabolic process [GO:0050655]	extracellular exosome [GO:0070062]; Golgi membrane [GO:0000139]; integral component of membrane [GO:0016021]; N-acetylgalactosamine 4-O-sulfotransferase activity [GO:0001537]; phosphate ion binding [GO:0042301]; sulfotransferase activity [GO:0008146]; carbohydrate biosynthetic process [GO:0016051]; dermatan sulfate biosynthetic process [GO:0030208]; dermatan sulfate proteoglycan metabolic process [GO:0050655]
Q6ZVM7	TOM1L2	TOM1-like protein 2 OS	55.5	10.98	3.46	1.41E-05	4.85	negative regulation of mitotic nuclear division [GO:0045839]; protein transport [GO:0015031]; signal transduction [GO:0007165]	endosome [GO:0005768]; extracellular exosome [GO:0070062]; membrane [GO:0016020]; clathrin binding [GO:0030276]; phosphatidylinositol binding [GO:0035091]; protein kinase binding [GO:0019901]; ubiquitin binding [GO:0043130]; negative regulation of mitotic nuclear division [GO:0045839]; protein transport [GO:0015031]; signal transduction [GO:0007165]

P27361	MAPK3	Mitogen-activated protein kinase 3 OS	43.1	10.56	3.40	6.69E-05	4.17	apoptotic process [GO:0006915]; Bergmann glial cell differentiation [GO:0060020]; BMP signaling pathway [GO:0030509]; cardiac neural crest cell development involved in heart development [GO:0061308]; cartilage development [GO:0051216]; caveolin-mediated endocytosis [GO:0072584]; cell cycle [GO:0007049]; cell surface receptor signaling pathway [GO:0007166]; cellular response to amino acid starvation [GO:0034198]; cellular response to cadmium ion [GO:0071276]; cellular response to dopamine [GO:1903351]; cellular response to mechanical stimulus [GO:0071260]; cellular response to reactive oxygen species [GO:0034614]; cellular response to tumor necrosis factor [GO:0071356]; DNA damage induced protein phosphorylation [GO:0006975]; ERK1 and ERK2 cascade [GO:0070371]; face development [GO:0060324]; interleukin-1-mediated signaling pathway [GO:0070498]; intracellular signal transduction [GO:0035556]; lipopolysaccharide-mediated signaling pathway [GO:0031663]; lung morphogenesis [GO:0060425]; MAPK cascade [GO:0000165]; negative regulation of apolipoprotein binding [GO:2000657]; outer ear morphogenesis [GO:0042473]; peptidyl-tyrosine autophosphorylation [GO:0038083]; phosphorylation [GO:0016310]; positive regulation of cyclase activity [GO:0031281]; positive regulation of ERK1 and ERK2 cascade [GO:0070374]; positive regulation of gene expression [GO:0010628]; positive regulation of histone acetylation [GO:0035066]; positive regulation of histone phosphorylation [GO:0033129]; positive regulation of macrophage chemotaxis [GO:0010759]; positive regulation of macrophage proliferation [GO:0120041]; positive	caveola [GO:0005901]; cytoplasm [GO:0005737]; cytoskeleton [GO:0005856]; cytosol [GO:0005829]; early endosome [GO:0005769]; endoplasmic reticulum lumen [GO:0005788]; focal adhesion [GO:0005925]; Golgi apparatus [GO:0005794]; late endosome [GO:0005770]; mitochondrion [GO:0005739]; nuclear envelope [GO:0005635]; nucleoplasm [GO:0005654]; nucleus [GO:0005634]; plasma membrane [GO:0005886]; pseudopodium [GO:0031143]; ATP binding [GO:0005524]; identical protein binding [GO:0042802]; MAP kinase activity [GO:0004707]; MAP kinase kinase activity [GO:0004708]; phosphatase binding [GO:0019902]; phosphotyrosine residue binding [GO:0001784]; protein serine kinase activity [GO:0106310]; protein serine/threonine kinase activity [GO:0004674]; apoptotic process [GO:0006915]; Bergmann glial cell differentiation [GO:0060020]; BMP signaling pathway [GO:0030509]; cardiac neural crest cell development involved in heart development [GO:0061308]; cartilage development [GO:0051216]; caveolin-mediated endocytosis [GO:0072584]; cell cycle [GO:0007049]; cell surface receptor signaling pathway [GO:0007166]; cellular response to amino acid starvation [GO:0034198]; cellular response to cadmium ion [GO:0071276]; cellular response to dopamine [GO:1903351]; cellular response to mechanical stimulus [GO:0071260]; cellular response to reactive oxygen species [GO:0034614]; cellular response to tumor necrosis factor [GO:0071356]; DNA damage induced protein phosphorylation [GO:0006975]; ERK1 and ERK2 cascade [GO:0070371]; face development [GO:0060324]; interleukin-1-mediated signaling pathway [GO:0070498]; intracellular signal transduction [GO:0035556]; lipopolysaccharide-mediated signaling pathway [GO:0031663]; lung morphogenesis [GO:0060425]; MAPK cascade [GO:0000165]; negative regulation of apolipoprotein binding [GO:2000657]; outer ear morphogenesis [GO:0042473]; peptidyl-tyrosine

								regulation of protein phosphorylation [GO:0001934]; positive regulation of telomerase activity [GO:0051973]; positive regulation of telomere capping [GO:1904355]; positive regulation of telomere maintenance via telomerase [GO:0032212]; positive regulation of transcription by RNA polymerase II [GO:0045944]; positive regulation of xenophagy [GO:1904417]; protein phosphorylation [GO:0006468]; regulation of cellular pH [GO:0030641]; regulation of cytoskeleton organization [GO:0051493]; regulation of DNA-binding transcription factor activity [GO:0051090]; regulation of early endosome to late endosome transport [GO:2000641]; regulation of Golgi inheritance [GO:0090170]; regulation of ossification [GO:0030278]; regulation of stress-activated MAPK cascade [GO:0032872]; response to epidermal growth factor [GO:0070849]; response to exogenous dsRNA [GO:0043330]; sensory perception of pain [GO:0019233]; stress-activated MAPK cascade [GO:0051403]; thymus development [GO:0048538]; thyroid gland development [GO:0030878]; trachea formation [GO:0060440]; transcription, DNA-templated [GO:0006351]; xenophagy [GO:0098792]	autophosphorylation [GO:0038083]; phosphorylation [GO:0016310]; positive regulation of cyclase activity [GO:0031281]; positive regulation of ERK1 and ERK2 cascade [GO:0070374]; positive regulation of gene expression [GO:0010628]; positive regulation of histone acetylation [GO:0035066]; positive regulation of histone phosphorylation [GO:0033129]; positive regulation of macrophage chemotaxis [GO:0010759]; positive regulation of macrophage proliferation [GO:0120041]; positive regulation of protein phosphorylation [GO:0001934]; positive regulation of telomerase activity [GO:0051973]; positive regulation of telomere capping [GO:1904355]; positive regulation of telomere maintenance via telomerase [GO:0032212]; positive regulation of transcription by RNA polymerase II [GO:0045944]; positive regulation of xenophagy [GO:1904417]; protein phosphorylation [GO:0006468]; regulation of cellular pH [GO:0030641]; regulation of cytoskeleton organization [GO:0051493]; regulation of DNA-binding transcription factor activity [GO:0051090]; regulation of early endosome to late endosome transport [GO:2000641]; regulation of Golgi inheritance [GO:0090170]; regulation of ossification [GO:0030278]; regulation of stress-activated MAPK cascade [GO:0032872]; response to epidermal growth factor [GO:0070849]; response to exogenous dsRNA [GO:0043330]; sensory perception of pain [GO:0019233]; stress-activated MAPK cascade [GO:0051403]; thymus development [GO:0048538]; thyroid gland development [GO:0030878]; trachea formation [GO:0060440]; transcription, DNA-templated [GO:0006351]; xenophagy [GO:0098792]
O75907	DGAT1	Diacylglycerol O-acyltransferase 1 OS	55.2	10.54	3.40	2.30E-05	4.64	diacylglycerol metabolic process [GO:0046339]; fatty acid homeostasis [GO:0055089]; lipid storage [GO:0019915]; long-chain fatty-acyl-CoA metabolic process [GO:0035336]; monoacylglycerol biosynthetic process [GO:0006640]; triglyceride biosynthetic process [GO:0019432];	endoplasmic reticulum membrane [GO:0005789]; integral component of membrane [GO:0016021]; plasma membrane [GO:0005886]; specific granule membrane [GO:0035579]; 2-acylglycerol O-acyltransferase activity [GO:0003846]; acyltransferase activity [GO:0016746]; diacylglycerol O-acyltransferase activity [GO:0004144]; identical

								triglyceride metabolic process [GO:0006641]; very-low-density lipoprotein particle assembly [GO:0034379]	protein binding [GO:0042802]; O-acyltransferase activity [GO:0008374]; retinol O-fatty-acyltransferase activity [GO:0050252]; diacylglycerol metabolic process [GO:0046339]; fatty acid homeostasis [GO:0055089]; lipid storage [GO:0019915]; long-chain fatty-acyl-CoA metabolic process [GO:0035336]; monoacylglycerol biosynthetic process [GO:0006640]; triglyceride biosynthetic process [GO:0019432]; triglyceride metabolic process [GO:0006641]; very-low-density lipoprotein particle assembly [GO:0034379]
O75874	IDH1	Isocitrate dehydrogenase [NADP] cytoplasmic OS	46.6	9.92	3.31	2.22E-04	3.65	2-oxoglutarate metabolic process [GO:0006103]; female gonad development [GO:0008585]; glutathione metabolic process [GO:0006749]; glyoxylate cycle [GO:0006097]; isocitrate metabolic process [GO:0006102]; NADP metabolic process [GO:0006739]; regulation of phospholipid biosynthetic process [GO:0071071]; regulation of phospholipid catabolic process [GO:0060696]; response to oxidative stress [GO:0006979]; response to steroid hormone [GO:0048545]; tricarboxylic acid cycle [GO:0006099]	cytoplasm [GO:0005737]; cytosol [GO:0005829]; extracellular exosome [GO:0070062]; extracellular region [GO:0005576]; ficolin-1-rich granule lumen [GO:1904813]; mitochondrion [GO:0005739]; peroxisomal matrix [GO:0005782]; peroxisome [GO:0005777]; secretory granule lumen [GO:0034774]; tertiary granule lumen [GO:1904724]; cadherin binding [GO:0045296]; identical protein binding [GO:0042802]; isocitrate dehydrogenase (NADP+) activity [GO:0004450]; magnesium ion binding [GO:0000287]; NAD binding [GO:0051287]; NADP binding [GO:0050661]; protein homodimerization activity [GO:0042803]; 2-oxoglutarate metabolic process [GO:0006103]; female gonad development [GO:0008585]; glutathione metabolic process [GO:0006749]; glyoxylate cycle [GO:0006097]; isocitrate metabolic process [GO:0006102]; NADP metabolic process [GO:0006739]; regulation of phospholipid biosynthetic process [GO:0071071]; regulation of phospholipid catabolic process [GO:0060696]; response to oxidative stress [GO:0006979]; response to steroid hormone [GO:0048545]; tricarboxylic acid cycle [GO:0006099]
Q96SL4	GPX7	Glutathione peroxidase 7 OS	21	9.86	3.30	2.15E-04	3.67	cellular response to oxidative stress [GO:0034599]	endoplasmic reticulum [GO:0005783]; endoplasmic reticulum lumen [GO:0005788]; extracellular region [GO:0005576]; catalase activity [GO:0004096]; glutathione peroxidase activity [GO:0004602];

									peroxidase activity [GO:0004601]; cellular response to oxidative stress [GO:0034599]
Q9Y2E5	MAN2B2	Epididymis-specific alpha-mannosidase OS	113.9	9.50	3.25	5.54E-05	4.26	mannose metabolic process [GO:0006013]	extracellular exosome [GO:0070062]; lysosomal lumen [GO:0043202]; lysosome [GO:0005764]; alpha-mannosidase activity [GO:0004559]; carbohydrate binding [GO:0030246]; metal ion binding [GO:0046872]; mannose metabolic process [GO:0006013]
P81277	PRLH	Prolactin-releasing peptide OS	9.6	9.33	3.22	2.77E-04	3.56	autonomic nervous system development [GO:0048483]; energy reserve metabolic process [GO:0006112]; fat cell differentiation [GO:0045444]; feeding behavior [GO:0007631]; G protein-coupled receptor signaling pathway [GO:0007186]; lipid metabolic process [GO:0006629]; reduction of food intake in response to dietary excess [GO:0002023]; regulation of multicellular organism growth [GO:0040014]; response to glucose [GO:0009749]; response to insulin [GO:0032868]; response to peptide hormone [GO:0043434]; tissue homeostasis [GO:0001894]	cytoplasm [GO:0005737]; extracellular region [GO:0005576]; hormone activity [GO:0005179]; neuropeptide hormone activity [GO:0005184]; prolactin-releasing peptide receptor binding [GO:0031861]; autonomic nervous system development [GO:0048483]; energy reserve metabolic process [GO:0006112]; fat cell differentiation [GO:0045444]; feeding behavior [GO:0007631]; G protein-coupled receptor signaling pathway [GO:0007186]; lipid metabolic process [GO:0006629]; reduction of food intake in response to dietary excess [GO:0002023]; regulation of multicellular organism growth [GO:0040014]; response to glucose [GO:0009749]; response to insulin [GO:0032868]; response to peptide hormone [GO:0043434]; tissue homeostasis [GO:0001894]
P0DML3	CSH2	Chorionic somatomammotropin hormone 2 OS	25	9.28	3.21	3.35E-06	5.48	animal organ development [GO:0048513]; growth hormone receptor signaling pathway [GO:0060396]; positive regulation of growth [GO:0045927]; positive regulation of receptor signaling pathway via JAK-STAT [GO:0046427]; positive regulation of tyrosine phosphorylation of STAT protein [GO:0042531]; response to nutrient levels [GO:0031667]	endoplasmic reticulum [GO:0005783]; extracellular space [GO:0005615]; vesicle [GO:0031982]; growth factor activity [GO:0008083]; growth hormone receptor binding [GO:0005131]; hormone activity [GO:0005179]; metal ion binding [GO:0046872]; animal organ development [GO:0048513]; growth hormone receptor signaling pathway [GO:0060396]; positive regulation of growth [GO:0045927]; positive regulation of receptor signaling pathway via JAK-STAT [GO:0046427]; positive regulation of tyrosine phosphorylation of STAT protein [GO:0042531]; response to nutrient levels [GO:0031667]

Q9BSH5	HDHD3	Haloacid dehalogenase-like hydrolase domain-containing protein 3 OS	28	9.26	3.21	4.76E-04	3.32		intracellular membrane-bounded organelle [GO:0043231]; nucleolus [GO:0005730]; nucleus [GO:0005634]
P23490	LORICRIN	Loricrin OS	25.7	9.22	3.20	4.72E-04	3.33	keratinization [GO:0031424]; keratinocyte differentiation [GO:0030216]; peptide cross-linking [GO:0018149]	cornified envelope [GO:0001533]; cytoplasm [GO:0005737]; cytosol [GO:0005829]; nucleoplasm [GO:0005654]; structural constituent of cytoskeleton [GO:0005200]; structural constituent of skin epidermis [GO:0030280]; keratinization [GO:0031424]; keratinocyte differentiation [GO:0030216]; peptide cross-linking [GO:0018149]
Q13835	PKP1	Plakophilin-1 OS	82.8	8.90	3.15	5.08E-04	3.29	cell adhesion [GO:0007155]; cell-cell adhesion [GO:0098609]; cell-cell junction assembly [GO:0007043]; intermediate filament bundle assembly [GO:0045110]; negative regulation of mRNA catabolic process [GO:1902373]; positive regulation of gene expression [GO:0010628]; signal transduction [GO:0007165]	adherens junction [GO:0005912]; cornified envelope [GO:0001533]; cytoplasm [GO:0005737]; desmosome [GO:0030057]; ficolin-1-rich granule membrane [GO:0101003]; intermediate filament [GO:0005882]; messenger ribonucleoprotein complex [GO:1990124]; nucleoplasm [GO:0005654]; nucleus [GO:0005634]; plasma membrane [GO:0005886]; cadherin binding [GO:0045296]; intermediate filament binding [GO:0019215]; lamin binding [GO:0005521]; structural constituent of skin epidermis [GO:0030280]; cell adhesion [GO:0007155]; cell-cell adhesion [GO:0098609]; cell-cell junction assembly [GO:0007043]; intermediate filament bundle assembly [GO:0045110]; negative regulation of mRNA catabolic process [GO:1902373]; positive regulation of gene expression [GO:0010628]; signal transduction [GO:0007165]
Q5BN46	C9orf116	UPF0691 protein C9orf116 OS	15.3	8.84	3.14	2.67E-04	3.57	axoneme assembly [GO:0035082]; cellular response to DNA damage stimulus [GO:0006974]; cellular response to UV-C [GO:0071494]; cilium movement [GO:0003341]; determination of left/right symmetry [GO:0007368]; establishment of left/right asymmetry [GO:0061966]; regulation of gene expression [GO:0010468]	axonemal microtubule [GO:0005879]; cytoplasm [GO:0005737]; nucleus [GO:0005634]; axoneme assembly [GO:0035082]; cellular response to DNA damage stimulus [GO:0006974]; cellular response to UV-C [GO:0071494]; cilium movement [GO:0003341]; determination of left/right symmetry [GO:0007368]; establishment of left/right asymmetry [GO:0061966]; regulation of gene expression [GO:0010468]

Q6ZMU5	TRIM72	Tripartite motif-containing protein 72 OS	52.7	7.41	2.89	2.64E-05	4.58	exocytosis [GO:0006887]; muscle organ development [GO:0007517]; muscle system process [GO:0003012]; negative regulation of insulin receptor signaling pathway [GO:0046627]; negative regulation of insulin-like growth factor receptor signaling pathway [GO:0043569]; negative regulation of myotube differentiation [GO:0010832]; plasma membrane repair [GO:0001778]; proteasome-mediated ubiquitin-dependent protein catabolic process [GO:0043161]; protein homooligomerization [GO:0051260]; protein ubiquitination [GO:0016567]	cytoplasm [GO:0005737]; cytoplasmic vesicle membrane [GO:0030659]; sarcolemma [GO:0042383]; identical protein binding [GO:0042802]; phosphatidylserine binding [GO:0001786]; ubiquitin conjugating enzyme binding [GO:0031624]; ubiquitin protein ligase activity [GO:0061630]; zinc ion binding [GO:0008270]; exocytosis [GO:0006887]; muscle organ development [GO:0007517]; muscle system process [GO:0003012]; negative regulation of insulin receptor signaling pathway [GO:0046627]; negative regulation of insulin-like growth factor receptor signaling pathway [GO:0043569]; negative regulation of myotube differentiation [GO:0010832]; plasma membrane repair [GO:0001778]; proteasome-mediated ubiquitin-dependent protein catabolic process [GO:0043161]; protein homooligomerization [GO:0051260]; protein ubiquitination [GO:0016567]
O43933	PEX1	Peroxisome biogenesis factor 1 OS	142.8	7.11	2.83	1.39E-02	1.86	microtubule-based peroxisome localization [GO:0060152]; peroxisome organization [GO:0007031]; protein import into peroxisome matrix [GO:0016558]; protein targeting to peroxisome [GO:0006625]	cytoplasm [GO:0005737]; cytosol [GO:0005829]; extracellular exosome [GO:0070062]; peroxisomal membrane [GO:0005778]; peroxisome [GO:0005777]; ATP binding [GO:0005524]; ATP hydrolysis activity [GO:0016887]; protein C-terminus binding [GO:0008022]; protein-containing complex binding [GO:0044877]; microtubule-based peroxisome localization [GO:0060152]; peroxisome organization [GO:0007031]; protein import into peroxisome matrix [GO:0016558]; protein targeting to peroxisome [GO:0006625]
O60678	PRMT3	Protein arginine N-methyltransferase 3 OS	59.9	6.74	2.75	6.56E-05	4.18	dendritic spine morphogenesis [GO:0060997]; negative regulation of protein ubiquitination [GO:0031397]; negative regulation of retinoic acid biosynthetic process [GO:1900053]; peptidyl-arginine methylation, to asymmetrical-dimethyl arginine [GO:0019919]; protein methylation [GO:0006479]	cytoplasm [GO:0005737]; cytosol [GO:0005829]; metal ion binding [GO:0046872]; methyltransferase activity [GO:0008168]; modified amino acid binding [GO:0072341]; protein-arginine N-methyltransferase activity [GO:0016274]; protein-arginine omega-N asymmetric methyltransferase activity [GO:0035242]; protein-arginine omega-N monomethyltransferase activity [GO:0035241]; ribosome binding [GO:0043022]; dendritic spine morphogenesis [GO:0060997]; negative regulation of protein ubiquitination [GO:0031397]; negative

									regulation of retinoic acid biosynthetic process [GO:1900053]; peptidyl-arginine methylation, to asymmetrical-dimethyl arginine [GO:0019919]; protein methylation [GO:0006479]
Q8TF72	SHROOM3	Protein Shroom3 OS	216.7	6.71	2.75	6.83E-05	4.17	actin filament organization [GO:0007015]; apical protein localization [GO:0045176]; cell morphogenesis [GO:0000902]; cellular pigment accumulation [GO:0043482]; epithelial cell development [GO:0002064]; neural tube closure [GO:0001843]; pattern specification process [GO:0007389]; regulation of cell shape [GO:0008360]	adherens junction [GO:0005912]; apical junction complex [GO:0043296]; apical plasma membrane [GO:0016324]; cortical actin cytoskeleton [GO:0030864]; cytoskeleton [GO:0005856]; microtubule [GO:0005874]; actin filament binding [GO:0051015]; actin filament organization [GO:0007015]; apical protein localization [GO:0045176]; cell morphogenesis [GO:0000902]; cellular pigment accumulation [GO:0043482]; epithelial cell development [GO:0002064]; neural tube closure [GO:0001843]; pattern specification process [GO:0007389]; regulation of cell shape [GO:0008360]
Q5T749	KPRP	Keratinocyte proline-rich protein OS	64.1	6.14	2.62	7.77E-04	3.11		cytoplasm [GO:0005737]; extracellular exosome [GO:0070062]
Q96P63	SERPINB12	Serpin B12 OS	46.2	6.12	2.61	1.69E-03	2.77	hematopoietic progenitor cell differentiation [GO:0002244]; negative regulation of endopeptidase activity [GO:0010951]; negative regulation of protein catabolic process [GO:0042177]	collagen-containing extracellular matrix [GO:0062023]; cornified envelope [GO:0001533]; cytoplasm [GO:0005737]; extracellular space [GO:0005615]; ficolin-1-rich granule membrane [GO:0101003]; plasma membrane [GO:0005886]; enzyme binding [GO:0019899]; serine-type endopeptidase inhibitor activity [GO:0004867]; hematopoietic progenitor cell differentiation [GO:0002244]; negative regulation of endopeptidase activity [GO:0010951]; negative regulation of protein catabolic process [GO:0042177]
O60524	NEMF	Nuclear export mediator factor NEMF OS	122.9	5.99	2.58	2.51E-03	2.60	CAT tailing [GO:0140708]; nuclear export [GO:0051168]; protein-containing complex assembly [GO:0065003]; rescue of stalled ribosome [GO:0072344]; ribosome-associated ubiquitin-dependent protein catabolic process [GO:1990116]	cytosol [GO:0005829]; nucleus [GO:0005634]; RQC complex [GO:1990112]; ribosomal large subunit binding [GO:0043023]; tRNA binding [GO:0000049]; CAT tailing [GO:0140708]; nuclear export [GO:0051168]; protein-containing complex assembly [GO:0065003]; rescue of stalled ribosome [GO:0072344]; ribosome-associated ubiquitin-dependent protein catabolic process [GO:1990116]

Q5T653	MRPL2	39S ribosomal protein L2, mitochondrial OS	33.3	5.73	2.52	5.71E-03	2.24	mitochondrial translation [GO:0032543]	mitochondrial inner membrane [GO:0005743]; mitochondrial large ribosomal subunit [GO:0005762]; mitochondrion [GO:0005739]; nucleoplasm [GO:0005654]; RNA binding [GO:0003723]; structural constituent of ribosome [GO:0003735]; mitochondrial translation [GO:0032543]
Q6ZUU3	FOXL2NB	FOXL2 neighbor protein OS	18.6	5.65	2.50	2.44E-02	1.61		fibrillar center [GO:0001650]
Q86YZ3	HRNR	Hornerin OS	282.2	5.59	2.48	3.15E-04	3.50	cell envelope organization [GO:0043163]; establishment of skin barrier [GO:0061436]; keratinization [GO:0031424]	azurophil granule lumen [GO:0035578]; collagen-containing extracellular matrix [GO:0062023]; cornified envelope [GO:0001533]; cytoplasm [GO:0005737]; extracellular exosome [GO:0070062]; extracellular region [GO:0005576]; keratohyalin granule [GO:0036457]; nucleus [GO:0005634]; perinuclear region of cytoplasm [GO:0048471]; calcium ion binding [GO:0005509]; transition metal ion binding [GO:0046914]; cell envelope organization [GO:0043163]; establishment of skin barrier [GO:0061436]; keratinization [GO:0031424]
Q9Y4Z0	LSM4	U6 snRNA-associated Sm-like protein LSm4 OS	15.3	5.58	2.48	2.87E-03	2.54	mRNA splicing, via spliceosome [GO:0000398]; nuclear-transcribed mRNA catabolic process [GO:0000956]; P-body assembly [GO:0033962]; RNA splicing [GO:0008380]; spliceosomal snRNP assembly [GO:0000387]	cytosol [GO:0005829]; Lsm2-8 complex [GO:0120115]; membrane [GO:0016020]; nucleoplasm [GO:0005654]; nucleus [GO:0005634]; P-body [GO:0000932]; protein-containing complex [GO:0032991]; spliceosomal tri-snRNP complex [GO:0097526]; U2-type precatalytic spliceosome [GO:0071005]; U4/U6 x U5 tri-snRNP complex [GO:0046540]; U6 snRNP [GO:0005688]; PH domain binding [GO:0042731]; RNA binding [GO:0003723]; U6 snRNA binding [GO:0017070]; mRNA splicing, via spliceosome [GO:0000398]; nuclear-transcribed mRNA catabolic process [GO:0000956]; P-body assembly [GO:0033962]; RNA splicing [GO:0008380]; spliceosomal snRNP assembly [GO:0000387]

P14923	JUP	Junction plakoglobin OS	81.7	5.57	2.48	3.22E-04	3.49	bundle of His cell-Purkinje myocyte adhesion involved in cell communication [GO:0086073]; canonical Wnt signaling pathway [GO:0060070]; cell migration [GO:0016477]; cell-cell adhesion [GO:0098609]; cellular response to indole-3-methanol [GO:0071681]; desmosome assembly [GO:0002159]; detection of mechanical stimulus [GO:0050982]; endothelial cell-cell adhesion [GO:0071603]; negative regulation of blood vessel endothelial cell migration [GO:0043537]; positive regulation of angiogenesis [GO:0045766]; positive regulation of canonical Wnt signaling pathway [GO:0090263]; positive regulation of cell-matrix adhesion [GO:0001954]; positive regulation of DNA-binding transcription factor activity [GO:0051091]; positive regulation of protein import into nucleus [GO:0042307]; positive regulation of transcription by RNA polymerase II [GO:0045944]; protein localization to plasma membrane [GO:0072659]; regulation of cell population proliferation [GO:0042127]; regulation of heart rate by cardiac conduction [GO:0086091]; regulation of ventricular cardiac muscle cell action potential [GO:0098911]; skin development [GO:0043588]	actin cytoskeleton [GO:0015629]; adherens junction [GO:0005912]; apicolateral plasma membrane [GO:0016327]; catenin complex [GO:0016342]; cell-cell junction [GO:0005911]; cornified envelope [GO:0001533]; cytoplasm [GO:0005737]; cytoplasmic side of plasma membrane [GO:0009898]; cytoskeleton [GO:0005856]; cytosol [GO:0005829]; desmosome [GO:0030057]; extracellular exosome [GO:0070062]; extracellular region [GO:0005576]; fascia adherens [GO:0005916]; ficolin-1-rich granule lumen [GO:1904813]; focal adhesion [GO:0005925]; gamma-catenin-TCF7L2 complex [GO:0071665]; intercalated disc [GO:0014704]; intermediate filament [GO:0005882]; lateral plasma membrane [GO:0016328]; nucleus [GO:0005634]; plasma membrane [GO:0005886]; protein-DNA complex [GO:0032993]; specific granule lumen [GO:0035580]; Z disc [GO:0030018]; zonula adherens [GO:0005915]; alpha-catenin binding [GO:0045294]; cadherin binding [GO:0045296]; cell adhesion molecule binding [GO:0050839]; cell adhesive protein binding involved in bundle of His cell-Purkinje myocyte communication [GO:0086083]; cytoskeletal protein-membrane anchor activity [GO:0106006]; DNA-binding transcription factor binding [GO:0140297]; protein homodimerization activity [GO:0042803]; protein phosphatase binding [GO:0019903]; protein tyrosine kinase binding [GO:1990782]; protein-containing complex binding [GO:0044877]; structural molecule activity [GO:0005198]; transcription coactivator activity [GO:0003713]; bundle of His cell-Purkinje myocyte adhesion involved in cell communication [GO:0086073]; canonical Wnt signaling pathway [GO:0060070]; cell migration [GO:0016477]; cell-cell adhesion [GO:0098609]; cellular response to indole-3-methanol [GO:0071681]; desmosome assembly [GO:0002159]; detection of mechanical stimulus [GO:0050982]; endothelial cell-cell adhesion [GO:0071603]; negative regulation of blood vessel endothelial cell migration [GO:0043537]; positive regulation of

										angiogenesis [GO:0045766]; positive regulation of canonical Wnt signaling pathway [GO:0090263]; positive regulation of cell-matrix adhesion [GO:0001954]; positive regulation of DNA-binding transcription factor activity [GO:0051091]; positive regulation of protein import into nucleus [GO:0042307]; positive regulation of transcription by RNA polymerase II [GO:0045944]; protein localization to plasma membrane [GO:0072659]; regulation of cell population proliferation [GO:0042127]; regulation of heart rate by cardiac conduction [GO:0086091]; regulation of ventricular cardiac muscle cell action potential [GO:0098911]; skin development [GO:0043588]
P31944	CASP14	Caspase-14 OS	27.7	5.55	2.47	1.69E-03	2.77	cornification [GO:0070268]; epidermis development [GO:0008544]; keratinization [GO:0031424]; proteolysis [GO:0006508]	cornified envelope [GO:0001533]; cytoplasm [GO:0005737]; cytosol [GO:0005829]; keratin filament [GO:0045095]; mitochondrion [GO:0005739]; nucleoplasm [GO:0005654]; nucleus [GO:0005634]; cysteine-type endopeptidase activity [GO:0004197]; cysteine-type endopeptidase activity involved in execution phase of apoptosis [GO:0097200]; cornification [GO:0070268]; epidermis development [GO:0008544]; keratinization [GO:0031424]; proteolysis [GO:0006508]
Q8NI35	PATJ	InaD-like protein OS	196.2	5.51	2.46	5.89E-03	2.23	establishment of apical/basal cell polarity [GO:0035089]; establishment or maintenance of epithelial cell apical/basal polarity [GO:0045197]; intracellular signal transduction [GO:0035556]; tight junction assembly [GO:0120192]	apical junction complex [GO:0043296]; apical plasma membrane [GO:0016324]; bicellular tight junction [GO:0005923]; cell junction [GO:0030054]; centriolar satellite [GO:0034451]; cytoplasm [GO:0005737]; cytosol [GO:0005829]; extracellular exosome [GO:0070062]; perinuclear region of cytoplasm [GO:0048471]; plasma membrane [GO:0005886]; establishment of apical/basal cell polarity [GO:0035089]; establishment or maintenance of epithelial cell apical/basal polarity [GO:0045197]; intracellular signal transduction [GO:0035556]; tight junction assembly [GO:0120192]

Q15517	CDSN	Corneodesmosin OS	51.5	5.36	2.42	8.40E-04	3.08	cell adhesion [GO:0007155]; cell-cell adhesion [GO:0098609]; corneocyte desquamation [GO:0003336]; epidermis development [GO:0008544]; keratinocyte differentiation [GO:0030216]; negative regulation of cornification [GO:1905716]; skin morphogenesis [GO:0043589]	cell-cell junction [GO:0005911]; cornified envelope [GO:0001533]; desmosome [GO:0030057]; extracellular region [GO:0005576]; plasma membrane [GO:0005886]; protein homodimerization activity [GO:0042803]; cell adhesion [GO:0007155]; cell-cell adhesion [GO:0098609]; corneocyte desquamation [GO:0003336]; epidermis development [GO:0008544]; keratinocyte differentiation [GO:0030216]; negative regulation of cornification [GO:1905716]; skin morphogenesis [GO:0043589]
O00754	MAN2B1	Lysosomal alpha-mannosidase OS	113.7	5.30	2.41	7.68E-03	2.11	learning or memory [GO:0007611]; mannose metabolic process [GO:0006013]; protein deglycosylation [GO:0006517]; protein modification process [GO:0036211]	azurophil granule lumen [GO:0035578]; extracellular exosome [GO:0070062]; extracellular region [GO:0005576]; extracellular space [GO:0005615]; intracellular membrane-bounded organelle [GO:0043231]; lysosomal lumen [GO:0043202]; lysosome [GO:0005764]; nucleoplasm [GO:0005654]; alpha-mannosidase activity [GO:0004559]; carbohydrate binding [GO:0030246]; metal ion binding [GO:0046872]; learning or memory [GO:0007611]; mannose metabolic process [GO:0006013]; protein deglycosylation [GO:0006517]; protein modification process [GO:0036211]
Q9H8W4	PLEKHF2	Pleckstrin homology domain-containing family F member 2 OS	27.8	5.18	2.37	3.64E-02	1.44	endosome organization [GO:0007032]; endosome to lysosome transport [GO:0008333]; protein transport [GO:0015031]	early endosome [GO:0005769]; early endosome membrane [GO:0031901]; endoplasmic reticulum [GO:0005783]; transport vesicle [GO:0030133]; metal ion binding [GO:0046872]; phosphatidylinositol binding [GO:0035091]; endosome organization [GO:0007032]; endosome to lysosome transport [GO:0008333]; protein transport [GO:0015031]
Q9P291	ARMCX1	Armadillo repeat-containing X-linked protein 1 OS	49.2	5.14	2.36	6.08E-04	3.22	hematopoietic stem cell homeostasis [GO:0061484]	integral component of membrane [GO:0016021]; mitochondrial outer membrane [GO:0005741]; mitochondrion [GO:0005739]; hematopoietic stem cell homeostasis [GO:0061484]
Q5T750	XP32	Skin-specific protein 32 OS	26.2	4.99	2.32	7.32E-03	2.14	epidermis development [GO:0008544]	epidermis development [GO:0008544]

P22735	TGM1	Protein-glutamine gamma-glutamyltransferase K OS	89.7	4.89	2.29	5.76E-02	1.24	cell envelope organization [GO:0043163]; keratinization [GO:0031424]; keratinocyte differentiation [GO:0030216]; peptide cross-linking [GO:0018149]; positive regulation of cell cycle [GO:0045787]; positive regulation of keratinocyte proliferation [GO:0010838]; protein modification process [GO:0036211]	cornified envelope [GO:0001533]; cytosol [GO:0005829]; extracellular exosome [GO:0070062]; intrinsic component of membrane [GO:0031224]; membrane [GO:0016020]; plasma membrane [GO:0005886]; identical protein binding [GO:0042802]; metal ion binding [GO:0046872]; protein-glutamine gamma-glutamyltransferase activity [GO:0003810]; cell envelope organization [GO:0043163]; keratinization [GO:0031424]; keratinocyte differentiation [GO:0030216]; peptide cross-linking [GO:0018149]; positive regulation of cell cycle [GO:0045787]; positive regulation of keratinocyte proliferation [GO:0010838]; protein modification process [GO:0036211]
O00151	PDLIM1	PDZ and LIM domain protein 1 OS	36	4.87	2.28	2.12E-02	1.67	actin cytoskeleton organization [GO:0030036]; establishment or maintenance of actin cytoskeleton polarity [GO:0030950]; fibroblast migration [GO:0010761]; heart development [GO:0007507]; maintenance of cell polarity [GO:0030011]; muscle structure development [GO:0061061]; regulation of transcription by RNA polymerase II [GO:0006357]; response to hypoxia [GO:0001666]; response to oxidative stress [GO:0006979]; stress fiber assembly [GO:0043149]	adherens junction [GO:0005912]; cytoplasm [GO:0005737]; cytoskeleton [GO:0005856]; filamentous actin [GO:0031941]; focal adhesion [GO:0005925]; stress fiber [GO:0001725]; transcription regulator complex [GO:0005667]; Z disc [GO:0030018]; actin binding [GO:0003779]; cadherin binding involved in cell-cell adhesion [GO:0098641]; metal ion binding [GO:0046872]; muscle alpha-actinin binding [GO:0051371]; transcription coactivator activity [GO:0003713]; actin cytoskeleton organization [GO:0030036]; establishment or maintenance of actin cytoskeleton polarity [GO:0030950]; fibroblast migration [GO:0010761]; heart development [GO:0007507]; maintenance of cell polarity [GO:0030011]; muscle structure development [GO:0061061]; regulation of transcription by RNA polymerase II [GO:0006357]; response to hypoxia [GO:0001666]; response to oxidative stress [GO:0006979]; stress fiber assembly [GO:0043149]
P49862	KLK7	Kallikrein-7 OS	27.5	4.84	2.28	5.03E-02	1.30	epidermis development [GO:0008544]; extracellular matrix disassembly [GO:0022617]; positive regulation of antibacterial peptide production [GO:0002803]; proteolysis [GO:0006508]	cornified envelope [GO:0001533]; epidermal lamellar body [GO:0097209]; extracellular region [GO:0005576]; extracellular space [GO:0005615]; secretory granule [GO:0030141]; metalloendopeptidase activity [GO:0004222]; peptidase activity [GO:0008233]; serine-type

									endopeptidase activity [GO:0004252]; serine-type peptidase activity [GO:0008236]; epidermis development [GO:0008544]; extracellular matrix disassembly [GO:0022617]; positive regulation of antibacterial peptide production [GO:0002803]; proteolysis [GO:0006508]
P05091	ALDH2	Aldehyde dehydrogenase, mitochondrial OS	56.3	4.78	2.26	2.19E-02	1.66	alcohol metabolic process [GO:0006066]; aldehyde catabolic process [GO:0046185]; carbohydrate metabolic process [GO:0005975]; ethanol catabolic process [GO:0006068]; regulation of dopamine biosynthetic process [GO:1903179]; regulation of serotonin biosynthetic process [GO:1905627]	extracellular exosome [GO:0070062]; mitochondrial matrix [GO:0005759]; mitochondrion [GO:0005739]; aldehyde dehydrogenase (NAD+) activity [GO:0004029]; aldehyde dehydrogenase [NAD(P)+] activity [GO:0004030]; carboxylic ester hydrolase activity [GO:0052689]; electron transfer activity [GO:0009055]; glyceraldehyde-3-phosphate dehydrogenase (NAD+) (non-phosphorylating) activity [GO:0043878]; NAD binding [GO:0051287]; nitroglycerin reductase activity [GO:0018547]; phenylacetaldehyde dehydrogenase activity [GO:0008957]; alcohol metabolic process [GO:0006066]; aldehyde catabolic process [GO:0046185]; carbohydrate metabolic process [GO:0005975]; ethanol catabolic process [GO:0006068]; regulation of dopamine biosynthetic process [GO:1903179]; regulation of serotonin biosynthetic process [GO:1905627]
Q86U86	PBRM1	Protein polybromo-1 OS	192.8	4.72	2.24	1.30E-02	1.89	chromatin remodeling [GO:0006338]; mitotic cell cycle [GO:0000278]; negative regulation of cell population proliferation [GO:0008285]; positive regulation of cell differentiation [GO:0045597]; positive regulation of double-strand break repair [GO:2000781]; positive regulation of myoblast differentiation [GO:0045663]; positive regulation of T cell differentiation [GO:0045582]; regulation of G0 to G1 transition [GO:0070316]; regulation of G1/S transition of mitotic cell cycle [GO:2000045]; regulation of mitotic metaphase/anaphase transition [GO:0030071]; regulation of nucleotide-excision repair [GO:2000819];	chromatin [GO:0000785]; kinetochore [GO:0000776]; nuclear chromosome [GO:0000228]; nuclear matrix [GO:0016363]; nucleoplasm [GO:0005654]; RSC-type complex [GO:0016586]; SWI/SNF complex [GO:0016514]; chromatin binding [GO:0003682]; DNA binding [GO:0003677]; chromatin remodeling [GO:0006338]; mitotic cell cycle [GO:0000278]; negative regulation of cell population proliferation [GO:0008285]; positive regulation of cell differentiation [GO:0045597]; positive regulation of double-strand break repair [GO:2000781]; positive regulation of myoblast differentiation [GO:0045663]; positive regulation of T cell differentiation [GO:0045582]; regulation of G0 to G1 transition [GO:0070316]; regulation of G1/S transition of mitotic cell cycle [GO:2000045];

								regulation of transcription by RNA polymerase II [GO:0006357]	regulation of mitotic metaphase/anaphase transition [GO:0030071]; regulation of nucleotide-excision repair [GO:2000819]; regulation of transcription by RNA polymerase II [GO:0006357]
Q9UPU7	TBC1D2B	TBC1 domain family member 2B OS	109.8	4.71	2.24	7.84E-02	1.11	activation of GTPase activity [GO:0090630]; endocytosis [GO:0006897]	cytosol [GO:0005829]; early endosome [GO:0005769]; GTPase activator activity [GO:0005096]; activation of GTPase activity [GO:0090630]; endocytosis [GO:0006897]
O95232	LUC7L3	Luc7-like protein 3 OS	51.4	4.70	2.23	1.99E-02	1.70	mRNA splice site selection [GO:0006376]; RNA splicing [GO:0008380]	nuclear speck [GO:0016607]; nucleoplasm [GO:0005654]; nucleus [GO:0005634]; U1 snRNP [GO:0005685]; U2-type prespliceosome [GO:0071004]; DNA binding [GO:0003677]; mRNA binding [GO:0003729]; RNA binding [GO:0003723]; mRNA splice site selection [GO:0006376]; RNA splicing [GO:0008380]
Q08188	TGM3	Protein-glutamine gamma-glutamyltransferase E OS	76.6	4.69	2.23	1.13E-02	1.95	cell envelope organization [GO:0043163]; hair follicle morphogenesis [GO:0031069]; keratinization [GO:0031424]; keratinocyte differentiation [GO:0030216]; peptide cross-linking [GO:0018149]; protein modification process [GO:0036211]	cytoplasm [GO:0005737]; extracellular exosome [GO:0070062]; extrinsic component of cytoplasmic side of plasma membrane [GO:0031234]; protein-containing complex [GO:0032991]; acyltransferase activity [GO:0016746]; calcium ion binding [GO:0005509]; catalytic activity [GO:0003824]; protein-glutamine gamma-glutamyltransferase activity [GO:0003810]; structural molecule activity [GO:0005198]; cell envelope organization [GO:0043163]; hair follicle morphogenesis [GO:0031069]; keratinization [GO:0031424]; keratinocyte differentiation [GO:0030216]; peptide cross-linking [GO:0018149]; protein modification process [GO:0036211]
Q01469	FABP5	Fatty acid-binding protein 5 OS	15.2	4.68	2.23	1.43E-03	2.85	epidermis development [GO:0008544]; fatty acid transport [GO:0015908]; glucose homeostasis [GO:0042593]; glucose metabolic process [GO:0006006]; lipid metabolic process [GO:0006629]; lipid transport across blood-brain barrier [GO:1990379]; long-chain fatty acid transport [GO:0015909]; negative regulation of glucose transmembrane transport [GO:0010829];	anchoring junction [GO:0070161]; azurophil granule lumen [GO:0035578]; cytoplasm [GO:0005737]; cytosol [GO:0005829]; extracellular exosome [GO:0070062]; extracellular region [GO:0005576]; nucleoplasm [GO:0005654]; nucleus [GO:0005634]; plasma membrane [GO:0005886]; postsynaptic density [GO:0014069]; secretory granule membrane [GO:0030667]; synapse [GO:0045202]; fatty acid binding [GO:0005504]; identical protein binding

								phosphatidylcholine biosynthetic process [GO:0006656]; positive regulation of cold-induced thermogenesis [GO:0120162]; positive regulation of peroxisome proliferator activated receptor signaling pathway [GO:0035360]; regulation of prostaglandin biosynthetic process [GO:0031392]; regulation of retrograde trans-synaptic signaling by endocanabinoid [GO:0099178]; regulation of sensory perception of pain [GO:0051930]	[GO:0042802]; lipid binding [GO:0008289]; long-chain fatty acid transporter activity [GO:0005324]; retinoic acid binding [GO:0001972]; epidermis development [GO:0008544]; fatty acid transport [GO:0015908]; glucose homeostasis [GO:0042593]; glucose metabolic process [GO:0006006]; lipid metabolic process [GO:0006629]; lipid transport across blood-brain barrier [GO:1990379]; long-chain fatty acid transport [GO:0015909]; negative regulation of glucose transmembrane transport [GO:0010829]; phosphatidylcholine biosynthetic process [GO:0006656]; positive regulation of cold-induced thermogenesis [GO:0120162]; positive regulation of peroxisome proliferator activated receptor signaling pathway [GO:0035360]; regulation of prostaglandin biosynthetic process [GO:0031392]; regulation of retrograde trans-synaptic signaling by endocanabinoid [GO:0099178]; regulation of sensory perception of pain [GO:0051930]
Q969Q0	RPL36AL	60S ribosomal protein L36a-like OS	12.5	4.67	2.22	7.07E-02	1.15	translation [GO:0006412]	cytosol [GO:0005829]; cytosolic large ribosomal subunit [GO:0022625]; endoplasmic reticulum [GO:0005783]; nucleus [GO:0005634]; plasma membrane [GO:0005886]; structural constituent of ribosome [GO:0003735]; translation [GO:0006412]
Q2TB90	HKDC1	Hexokinase HKDC1 OS	102.5	4.66	2.22	1.31E-02	1.88	carbohydrate phosphorylation [GO:0046835]; cellular glucose homeostasis [GO:0001678]; glucose 6-phosphate metabolic process [GO:0051156]; glucose metabolic process [GO:0006006]; glycolytic process [GO:0006096]	cytoplasm [GO:0005737]; cytosol [GO:0005829]; mitochondrial membrane [GO:0031966]; mitochondrion [GO:0005739]; ATP binding [GO:0005524]; fructokinase activity [GO:0008865]; glucokinase activity [GO:0004340]; glucose binding [GO:0005536]; carbohydrate phosphorylation [GO:0046835]; cellular glucose homeostasis [GO:0001678]; glucose 6-phosphate metabolic process [GO:0051156]; glucose metabolic process [GO:0006006]; glycolytic process [GO:0006096]
Q5D862	FLG2	Filaggrin-2 OS	247.9	4.60	2.20	1.42E-03	2.85	cell adhesion [GO:0007155]; epidermis morphogenesis [GO:0048730]; establishment of skin barrier [GO:0061436]	cornified envelope [GO:0001533]; cytoplasm [GO:0005737]; extracellular region [GO:0005576]; keratohyalin granule [GO:0036457]; nucleus [GO:0005634]; tertiary granule lumen [GO:1904724]; calcium ion binding [GO:0005509]; structural

									molecule activity [GO:0005198]; transition metal ion binding [GO:0046914]; cell adhesion [GO:0007155]; epidermis morphogenesis [GO:0048730]; establishment of skin barrier [GO:0061436]
P14550	AKR1A1	Aldo-keto reductase family 1 member A1 OS	36.6	4.55	2.18	1.76E-02	1.75	aldehyde catabolic process [GO:0046185]; cellular detoxification of aldehyde [GO:0110095]; D-glucuronate catabolic process [GO:0042840]; daunorubicin metabolic process [GO:0044597]; doxorubicin metabolic process [GO:0044598]; glucuronate catabolic process to xylulose 5-phosphate [GO:0019640]; glutathione derivative biosynthetic process [GO:1901687]; L-ascorbic acid biosynthetic process [GO:0019853]; lipid metabolic process [GO:0006629]	apical plasma membrane [GO:0016324]; cytosol [GO:0005829]; extracellular exosome [GO:0070062]; extracellular space [GO:0005615]; synapse [GO:0045202]; alditol:NADP+ 1-oxidoreductase activity [GO:0004032]; aldo-keto reductase (NADP) activity [GO:0004033]; allyl-alcohol dehydrogenase activity [GO:0047655]; glucuronolactone reductase activity [GO:0047941]; glycerol dehydrogenase [NADP+] activity [GO:0047956]; L-glucuronate reductase activity [GO:0047939]; methylglyoxal reductase (NADPH-dependent, acetol producing) [GO:1990002]; oxidoreductase activity, acting on NAD(P)H, nitrogenous group as acceptor [GO:0016657]; aldehyde catabolic process [GO:0046185]; cellular detoxification of aldehyde [GO:0110095]; D-glucuronate catabolic process [GO:0042840]; daunorubicin metabolic process [GO:0044597]; doxorubicin metabolic process [GO:0044598]; glucuronate catabolic process to xylulose 5-phosphate [GO:0019640]; glutathione derivative biosynthetic process [GO:1901687]; L-ascorbic acid biosynthetic process [GO:0019853]; lipid metabolic process [GO:0006629]
Q9NZT1	CALML5	Calmodulin-like protein 5 OS	15.9	4.46	2.16	5.32E-03	2.27	epidermis development [GO:0008544]; signal transduction [GO:0007165]	extracellular region [GO:0005576]; ficolin-1-rich granule lumen [GO:1904813]; calcium ion binding [GO:0005509]; enzyme regulator activity [GO:0030234]; epidermis development [GO:0008544]; signal transduction [GO:0007165]
Q9Y3Y2	CHTOP	Chromatin target of PRMT1 protein OS	26.4	4.46	2.16	1.22E-02	1.91	in utero embryonic development [GO:0001701]; mRNA export from nucleus [GO:0006406]; positive regulation of ATP-dependent activity [GO:0032781]; positive regulation of cell population proliferation [GO:0008284]; positive regulation of helicase	cytoplasmic ribonucleoprotein granule [GO:0036464]; nuclear speck [GO:0016607]; nucleolus [GO:0005730]; nucleoplasm [GO:0005654]; nucleus [GO:0005634]; transcription export complex [GO:0000346]; methyl-CpG binding [GO:0008327]; mRNA binding [GO:0003729]; RNA binding [GO:0003723]; in utero embryonic development

								activity [GO:0051096]; positive regulation of histone methylation [GO:0031062]	[GO:0001701]; mRNA export from nucleus [GO:0006406]; positive regulation of ATP-dependent activity [GO:0032781]; positive regulation of cell population proliferation [GO:0008284]; positive regulation of helicase activity [GO:0051096]; positive regulation of histone methylation [GO:0031062]
P32004	L1CAM	Neural cell adhesion molecule L1 OS	139.9	4.39	2.13	2.37E-02	1.63	axon development [GO:0061564]; axon guidance [GO:0007411]; cell adhesion [GO:0007155]; cell migration [GO:0016477]; cell-matrix adhesion [GO:0007160]; chemotaxis [GO:0006935]; homophilic cell adhesion via plasma membrane adhesion molecules [GO:0007156]; nervous system development [GO:0007399]; neuron projection development [GO:0031175]; positive regulation of axon extension [GO:0045773]; synapse organization [GO:0050808]	axon [GO:0030424]; axonal growth cone [GO:0044295]; cell surface [GO:0009986]; collagen-containing extracellular matrix [GO:0062023]; dendrite [GO:0030425]; focal adhesion [GO:0005925]; integral component of membrane [GO:0016021]; neuronal cell body [GO:0043025]; plasma membrane [GO:0005886]; axon guidance receptor activity [GO:0008046]; protein domain specific binding [GO:0019904]; axon development [GO:0061564]; axon guidance [GO:0007411]; cell adhesion [GO:0007155]; cell migration [GO:0016477]; cell-matrix adhesion [GO:0007160]; chemotaxis [GO:0006935]; homophilic cell adhesion via plasma membrane adhesion molecules [GO:0007156]; nervous system development [GO:0007399]; neuron projection development [GO:0031175]; positive regulation of axon extension [GO:0045773]; synapse organization [GO:0050808]
P18206	VCL	Vinculin OS	123.7	4.36	2.12	2.11E-03	2.68	adherens junction assembly [GO:0034333]; apical junction assembly [GO:0043297]; axon extension [GO:0048675]; cell adhesion [GO:0007155]; cell-matrix adhesion [GO:0007160]; epithelial cell-cell adhesion [GO:0090136]; lamellipodium assembly [GO:0030032]; maintenance of blood-brain barrier [GO:0035633]; morphogenesis of an epithelium [GO:0002009]; negative regulation of cell migration [GO:0030336]; platelet aggregation [GO:0070527]; protein localization to cell surface [GO:0034394]; regulation of establishment of endothelial barrier [GO:1903140]; regulation of focal adhesion assembly [GO:0051893]; regulation	adherens junction [GO:0005912]; brush border [GO:0005903]; cell-cell contact zone [GO:0044291]; cell-cell junction [GO:0005911]; cell-substrate junction [GO:0030055]; costamere [GO:0043034]; cytoplasm [GO:0005737]; cytoskeleton [GO:0005856]; cytosol [GO:0005829]; extracellular exosome [GO:0070062]; extracellular region [GO:0005576]; extracellular vesicle [GO:1903561]; fascia adherens [GO:0005916]; ficolin-1-rich granule lumen [GO:1904813]; focal adhesion [GO:0005925]; inner dense plaque of desmosome [GO:0090637]; membrane raft [GO:0045121]; outer dense plaque of desmosome [GO:0090636]; plasma membrane [GO:0005886]; podosome [GO:0002102]; protein-containing complex [GO:0032991]; sarcolemma [GO:0042383]; secretory granule lumen

								of protein localization to adherens junction [GO:1904702]	[GO:0034774]; specific granule lumen [GO:0035580]; terminal web [GO:1990357]; zonula adherens [GO:0005915]; actin binding [GO:0003779]; alpha-catenin binding [GO:0045294]; beta-catenin binding [GO:0008013]; cadherin binding [GO:0045296]; dystroglycan binding [GO:0002162]; structural molecule activity [GO:0005198]; ubiquitin protein ligase binding [GO:0031625]; adherens junction assembly [GO:0034333]; apical junction assembly [GO:0043297]; axon extension [GO:0048675]; cell adhesion [GO:0007155]; cell-matrix adhesion [GO:0007160]; epithelial cell-cell adhesion [GO:0090136]; lamellipodium assembly [GO:0030032]; maintenance of blood-brain barrier [GO:0035633]; morphogenesis of an epithelium [GO:0002009]; negative regulation of cell migration [GO:0030336]; platelet aggregation [GO:0070527]; protein localization to cell surface [GO:0034394]; regulation of establishment of endothelial barrier [GO:1903140]; regulation of focal adhesion assembly [GO:0051893]; regulation of protein localization to adherens junction [GO:1904702]
Q08554	DSC1	Desmocollin-1 OS	99.9	4.27	2.09	2.43E-03	2.61	cell-cell adhesion [GO:0098609]; homophilic cell adhesion via plasma membrane adhesion molecules [GO:0007156]	cell-cell junction [GO:0005911]; cornified envelope [GO:0001533]; desmosome [GO:0030057]; extracellular exosome [GO:0070062]; ficolin-1-rich granule membrane [GO:0101003]; gap junction [GO:0005921]; integral component of membrane [GO:0016021]; membrane [GO:0016020]; plasma membrane [GO:0005886]; calcium ion binding [GO:0005509]; cell-cell adhesion [GO:0098609]; homophilic cell adhesion via plasma membrane adhesion molecules [GO:0007156]
Q9Y5V3	MAGED1	Melanoma-associated antigen D1 OS	86.1	4.25	2.09	1.72E-02	1.76	circadian regulation of gene expression [GO:0032922]; negative regulation of epithelial cell proliferation [GO:0050680]; negative regulation of protein localization to nucleus [GO:1900181]; negative regulation of transcription by RNA polymerase II [GO:0000122]; positive regulation of apoptotic signaling pathway [GO:2001235];	chromatin [GO:0000785]; cytoplasm [GO:0005737]; nucleus [GO:0005634]; plasma membrane [GO:0005886]; protein-containing complex [GO:0032991]; identical protein binding [GO:0042802]; transcription coactivator activity [GO:0003713]; circadian regulation of gene expression [GO:0032922]; negative regulation of epithelial cell proliferation [GO:0050680]; negative

								positive regulation of branching involved in ureteric bud morphogenesis [GO:0090190]; positive regulation of MAP kinase activity [GO:0043406]; protein localization to nucleus [GO:0034504]; regulation of apoptotic process [GO:0042981]; regulation of circadian rhythm [GO:0042752]; regulation of transcription, DNA-templated [GO:0006355]	regulation of protein localization to nucleus [GO:1900181]; negative regulation of transcription by RNA polymerase II [GO:0000122]; positive regulation of apoptotic signaling pathway [GO:2001235]; positive regulation of branching involved in ureteric bud morphogenesis [GO:0090190]; positive regulation of MAP kinase activity [GO:0043406]; protein localization to nucleus [GO:0034504]; regulation of apoptotic process [GO:0042981]; regulation of circadian rhythm [GO:0042752]; regulation of transcription, DNA-templated [GO:0006355]
P48594	SERPINB4	Serpin B4 OS	44.8	4.15	2.05	3.04E-02	1.52	negative regulation of endopeptidase activity [GO:0010951]; negative regulation of peptidase activity [GO:0010466]; protection from natural killer cell mediated cytotoxicity [GO:0042270]; regulation of proteolysis [GO:0030162]	cytoplasm [GO:0005737]; extracellular space [GO:0005615]; enzyme binding [GO:0019899]; protease binding [GO:0002020]; serine-type endopeptidase inhibitor activity [GO:0004867]; negative regulation of endopeptidase activity [GO:0010951]; negative regulation of peptidase activity [GO:0010466]; protection from natural killer cell mediated cytotoxicity [GO:0042270]; regulation of proteolysis [GO:0030162]
Q14789	GOLGB1	Golgin subfamily B member 1 OS	375.8	4.14	2.05	7.19E-02	1.14	chondrocyte proliferation [GO:0035988]; Golgi organization [GO:0007030]; protein localization to pericentriolar material [GO:1905793]; regulation of transcription, DNA-templated [GO:0006355]	cis-Golgi network [GO:0005801]; cytosol [GO:0005829]; endoplasmic reticulum-Golgi intermediate compartment [GO:0005793]; Golgi apparatus [GO:0005794]; Golgi membrane [GO:0000139]; Golgi stack [GO:0005795]; integral component of membrane [GO:0016021]; membrane [GO:0016020]; RNA binding [GO:0003723]; sequence-specific DNA binding [GO:0043565]; chondrocyte proliferation [GO:0035988]; Golgi organization [GO:0007030]; protein localization to pericentriolar material [GO:1905793]; regulation of transcription, DNA-templated [GO:0006355]
Q96K49	TMEM87B	Transmembrane protein 87B OS	63.5	4.11	2.04	4.31E-02	1.37	retrograde transport, endosome to Golgi [GO:0042147]	cytosol [GO:0005829]; Golgi apparatus [GO:0005794]; Golgi membrane [GO:0000139]; integral component of membrane [GO:0016021]; membrane [GO:0016020]; retrograde transport, endosome to Golgi [GO:0042147]

P19971	TYMP	Thymidine phosphorylase OS	49.9	4.11	2.04	4.24E-02	1.37	angiogenesis [GO:0001525]; cell differentiation [GO:0030154]; chemotaxis [GO:0006935]; dTMP catabolic process [GO:0046074]; mitochondrial genome maintenance [GO:0000002]; pyrimidine nucleobase metabolic process [GO:0006206]; pyrimidine nucleoside metabolic process [GO:0006213]; regulation of gastric motility [GO:1905333]; regulation of myelination [GO:0031641]; regulation of transmission of nerve impulse [GO:0051969]	cytosol [GO:0005829]; 1,4-alpha-oligoglucan phosphorylase activity [GO:0004645]; growth factor activity [GO:0008083]; protein homodimerization activity [GO:0042803]; pyrimidine-nucleoside phosphorylase activity [GO:0016154]; thymidine phosphorylase activity [GO:0009032]; angiogenesis [GO:0001525]; cell differentiation [GO:0030154]; chemotaxis [GO:0006935]; dTMP catabolic process [GO:0046074]; mitochondrial genome maintenance [GO:0000002]; pyrimidine nucleobase metabolic process [GO:0006206]; pyrimidine nucleoside metabolic process [GO:0006213]; regulation of gastric motility [GO:1905333]; regulation of myelination [GO:0031641]; regulation of transmission of nerve impulse [GO:0051969]
Q9H1E3	NUCKS1	Nuclear ubiquitous casein and cyclin-dependent kinase substrate 1 OS	27.3	4.00	2.00	2.43E-02	1.61	cellular glucose homeostasis [GO:0001678]; cellular response to X-ray [GO:0071481]; chromatin organization [GO:0006325]; double-strand break repair via homologous recombination [GO:0000724]; gene conversion [GO:0035822]; interstrand cross-link repair [GO:0036297]; modulation by host of RNA binding by virus [GO:1990968]; modulation by host of viral genome replication [GO:0044827]; modulation by host of viral RNA-binding transcription factor activity [GO:1990969]; positive regulation by host of viral genome replication [GO:0044829]; positive regulation by host of viral transcription [GO:0043923]; positive regulation of insulin receptor signaling pathway [GO:0046628]; positive regulation of transcription by RNA polymerase II [GO:0045944]; regulation of DNA replication [GO:0006275]; regulation of DNA strand elongation [GO:0060382]; regulation of insulin receptor signaling pathway [GO:0046626]; replication fork processing [GO:0031297]	chromatin [GO:0000785]; cytoplasm [GO:0005737]; nucleolus [GO:0005730]; nucleoplasm [GO:0005654]; nucleus [GO:0005634]; chromatin binding [GO:0003682]; DNA-binding transcription factor binding [GO:0140297]; double-stranded DNA binding [GO:0003690]; RNA binding [GO:0003723]; single-stranded DNA binding [GO:0003697]; transcription coactivator activity [GO:0003713]; cellular glucose homeostasis [GO:0001678]; cellular response to X-ray [GO:0071481]; chromatin organization [GO:0006325]; double-strand break repair via homologous recombination [GO:0000724]; gene conversion [GO:0035822]; interstrand cross-link repair [GO:0036297]; modulation by host of RNA binding by virus [GO:1990968]; modulation by host of viral genome replication [GO:0044827]; modulation by host of viral RNA-binding transcription factor activity [GO:1990969]; positive regulation by host of viral genome replication [GO:0044829]; positive regulation by host of viral transcription [GO:0043923]; positive regulation of insulin receptor signaling pathway [GO:0046628]; positive regulation of transcription by RNA polymerase II [GO:0045944]; regulation of DNA replication [GO:0006275]; regulation of DNA strand elongation [GO:0060382]; regulation of insulin receptor signaling pathway

									[GO:0046626]; replication fork processing [GO:0031297]
Q15262	PTPRK	Receptor-type tyrosine-protein phosphatase kappa OS	162	3.97	1.99	3.71E-02	1.43	cell adhesion [GO:0007155]; cell migration [GO:0016477]; cellular response to reactive oxygen species [GO:0034614]; cellular response to UV [GO:0034644]; focal adhesion assembly [GO:0048041]; negative regulation of cell cycle [GO:0045786]; negative regulation of cell migration [GO:0030336]; negative regulation of cell population proliferation [GO:0008285]; negative regulation of keratinocyte proliferation [GO:0010839]; negative regulation of transcription, DNA-templated [GO:0045892]; protein dephosphorylation [GO:0006470]; protein localization to cell surface [GO:0034394]; signal transduction [GO:0007165]; transforming growth factor beta receptor signaling pathway [GO:0007179]	adherens junction [GO:0005912]; cell junction [GO:0030054]; cell surface [GO:0009986]; cell-cell junction [GO:0005911]; integral component of membrane [GO:0016021]; integral component of plasma membrane [GO:0005887]; intracellular membrane-bounded organelle [GO:0043231]; leading edge membrane [GO:0031256]; plasma membrane [GO:0005886]; beta-catenin binding [GO:0008013]; gamma-catenin binding [GO:0045295]; protein kinase binding [GO:0019901]; protein tyrosine phosphatase activity [GO:0004725]; transmembrane receptor protein tyrosine phosphatase activity [GO:0005001]; cell adhesion [GO:0007155]; cell migration [GO:0016477]; cellular response to reactive oxygen species [GO:0034614]; cellular response to UV [GO:0034644]; focal adhesion assembly [GO:0048041]; negative regulation of cell cycle [GO:0045786]; negative regulation of cell migration [GO:0030336]; negative regulation of cell population proliferation [GO:0008285]; negative regulation of keratinocyte proliferation [GO:0010839]; negative regulation of transcription, DNA-templated [GO:0045892]; protein dephosphorylation [GO:0006470]; protein localization to cell surface [GO:0034394]; signal transduction [GO:0007165]; transforming growth factor beta receptor signaling pathway [GO:0007179]
Q7Z2Z2	EFL1	Elongation factor-like GTPase 1 OS	125.4	3.95	1.98	3.38E-02	1.47	GTP metabolic process [GO:0046039]; mature ribosome assembly [GO:0042256]	ribonucleoprotein complex [GO:1990904]; GTP binding [GO:0005525]; GTPase activity [GO:0003924]; ribosome binding [GO:0043022]; translation elongation factor activity [GO:0003746];

									GTP metabolic process [GO:0046039]; mature ribosome assembly [GO:0042256]
P20930	FLG	Filaggrin OS	434.9	3.89	1.96	6.28E-03	2.20	establishment of skin barrier [GO:0061436]; keratinocyte differentiation [GO:0030216]; peptide cross-linking [GO:0018149]	collagen-containing extracellular matrix [GO:0062023]; cornified envelope [GO:0001533]; cytoplasmic ribonucleoprotein granule [GO:0036464]; cytosol [GO:0005829]; keratohyalin granule [GO:0036457]; nucleus [GO:0005634]; calcium ion binding [GO:0005509]; structural constituent of skin epidermis [GO:0030280]; transition metal ion binding [GO:0046914]; establishment of skin barrier [GO:0061436]; keratinocyte differentiation [GO:0030216]; peptide cross-linking [GO:0018149]
Q9UBS5	GABBR1	Gamma-aminobutyric acid type B receptor subunit 1 OS	108.3	3.83	1.94	3.46E-02	1.46	adenylate cyclase-inhibiting G protein-coupled receptor signaling pathway [GO:0007193]; gamma-aminobutyric acid signaling pathway [GO:0007214]; negative regulation of adenylate cyclase activity [GO:0007194]; negative regulation of cell population proliferation [GO:0008285]; negative regulation of dopamine secretion [GO:0033602]; negative regulation of epinephrine secretion [GO:0032811]; negative regulation of gamma-aminobutyric acid secretion [GO:0014053]; negative regulation of synaptic transmission [GO:0050805]; neuron-glial cell signaling [GO:0150099]; osteoblast differentiation [GO:0001649]; positive regulation of glutamate secretion [GO:0014049]; positive regulation of growth hormone secretion [GO:0060124]; response to ethanol [GO:0045471]; response to nicotine [GO:0035094]; synaptic transmission, GABAergic [GO:0051932]	anchoring junction [GO:0070161]; axolemma [GO:0030673]; dendritic shaft [GO:0043198]; dendritic spine [GO:0043197]; endoplasmic reticulum membrane [GO:0005789]; extracellular space [GO:0005615]; G protein-coupled GABA receptor complex [GO:1902712]; G protein-coupled receptor heterodimeric complex [GO:0038039]; GABA-ergic synapse [GO:0098982]; glutamatergic synapse [GO:0098978]; integral component of plasma membrane [GO:0005887]; integral component of postsynaptic membrane [GO:0099055]; integral component of presynaptic membrane [GO:0099056]; membrane raft [GO:0045121]; mitochondrial membrane [GO:0031966]; neuronal cell body [GO:0043025]; plasma membrane [GO:0005886]; Schaffer collateral - CA1 synapse [GO:0098685]; synaptic vesicle [GO:0008021]; extracellular matrix protein binding [GO:1990430]; G protein-coupled GABA receptor activity [GO:0004965]; G protein-coupled neurotransmitter receptor activity involved in regulation of postsynaptic membrane potential [GO:0099579]; protein heterodimerization activity [GO:0046982]; transmembrane signaling receptor activity [GO:0004888]; adenylate cyclase-inhibiting G protein-coupled receptor signaling pathway [GO:0007193]; gamma-aminobutyric acid signaling

									pathway [GO:0007214]; negative regulation of adenylate cyclase activity [GO:0007194]; negative regulation of cell population proliferation [GO:0008285]; negative regulation of dopamine secretion [GO:0033602]; negative regulation of epinephrine secretion [GO:0032811]; negative regulation of gamma-aminobutyric acid secretion [GO:0014053]; negative regulation of synaptic transmission [GO:0050805]; neuron-glial cell signaling [GO:0150099]; osteoblast differentiation [GO:0001649]; positive regulation of glutamate secretion [GO:0014049]; positive regulation of growth hormone secretion [GO:0060124]; response to ethanol [GO:0045471]; response to nicotine [GO:0035094]; synaptic transmission, GABAergic [GO:0051932]
Q8IV38	ANKMY2	Ankyrin repeat and MYND domain-containing protein 2 OS	49.3	3.74	1.90	5.11E-02	1.29		cilium [GO:0005929]; enzyme binding [GO:0019899]; metal ion binding [GO:0046872]
Q9ULE4	FAM184B	Protein FAM184B OS	121	3.71	1.89	6.13E-03	2.21		
P04003	C4BPA	C4b-binding protein alpha chain OS	67	3.68	1.88	5.87E-02	1.23	complement activation, classical pathway [GO:0006958]; innate immune response [GO:0045087]; negative regulation of complement activation, classical pathway [GO:0045959]; positive regulation of protein catabolic process [GO:0045732]; regulation of opsonization [GO:1903027]; response to symbiotic bacterium [GO:0009609]	blood microparticle [GO:0072562]; extracellular region [GO:0005576]; extracellular space [GO:0005615]; plasma membrane [GO:0005886]; RNA binding [GO:0003723]; complement activation, classical pathway [GO:0006958]; innate immune response [GO:0045087]; negative regulation of complement activation, classical pathway [GO:0045959]; positive regulation of protein catabolic process [GO:0045732]; regulation of opsonization [GO:1903027]; response to symbiotic bacterium [GO:0009609]
P08118	MSMB	Beta-microseminoprotein OS	12.9	3.60	1.85	6.90E-02	1.16		extracellular space [GO:0005615]; nucleus [GO:0005634]
Q6P589	TNFAIP8L2	Tumor necrosis factor alpha-induced protein 8-like protein 2 OS	20.5	3.49	1.80	3.43E-02	1.46	innate immune response [GO:0045087]; negative regulation of inflammatory response [GO:0050728]; negative regulation of T cell activation [GO:0050868]; regulation of	cytoplasm [GO:0005737]; innate immune response [GO:0045087]; negative regulation of inflammatory response [GO:0050728]; negative regulation of T cell activation [GO:0050868]; regulation of apoptotic

								apoptotic process [GO:0042981]; T cell activation [GO:0042110]	process [GO:0042981]; T cell activation [GO:0042110]
O75044	SRGAP2	SLIT-ROBO Rho GTPase-activating protein 2 OS	120.8	3.48	1.80	6.83E-02	1.17	actin filament severing [GO:0051014]; dendritic spine development [GO:0060996]; excitatory synapse assembly [GO:1904861]; extension of a leading process involved in cell motility in cerebral cortex radial glia guided migration [GO:0021816]; filopodium assembly [GO:0046847]; inhibitory synapse assembly [GO:1904862]; lamellipodium assembly involved in ameboidal cell migration [GO:0003363]; negative regulation of cell migration [GO:0030336]; negative regulation of neuron migration [GO:2001223]; neuron projection morphogenesis [GO:0048812]; positive regulation of GTPase activity [GO:0043547]; regulation of small GTPase mediated signal transduction [GO:0051056]; signal transduction [GO:0007165]; substrate adhesion-dependent cell spreading [GO:0034446]	anchoring junction [GO:0070161]; cytoplasm [GO:0005737]; cytosol [GO:0005829]; dendritic spine head [GO:0044327]; lamellipodium [GO:0030027]; nucleoplasm [GO:0005654]; phagocytic vesicle [GO:0045335]; plasma membrane [GO:0005886]; postsynaptic density [GO:0014069]; postsynaptic membrane [GO:0045211]; GTPase activator activity [GO:0005096]; identical protein binding [GO:0042802]; protein homodimerization activity [GO:0042803]; small GTPase binding [GO:0031267]; actin filament severing [GO:0051014]; dendritic spine development [GO:0060996]; excitatory synapse assembly [GO:1904861]; extension of a leading process involved in cell motility in cerebral cortex radial glia guided migration [GO:0021816]; filopodium assembly [GO:0046847]; inhibitory synapse assembly [GO:1904862]; lamellipodium assembly involved in ameboidal cell migration [GO:0003363]; negative regulation of cell migration [GO:0030336]; negative regulation of neuron migration [GO:2001223]; neuron projection morphogenesis [GO:0048812]; positive regulation of GTPase activity [GO:0043547]; regulation of small GTPase mediated signal transduction [GO:0051056]; signal transduction [GO:0007165]; substrate adhesion-dependent cell spreading [GO:0034446]
P19823	ITIH2	Inter-alpha-trypsin inhibitor heavy chain H2 OS	106.4	3.42	1.77	1.02E-02	1.99	hyaluronan metabolic process [GO:0030212]	blood microparticle [GO:0072562]; collagen-containing extracellular matrix [GO:0062023]; endoplasmic reticulum lumen [GO:0005788]; extracellular exosome [GO:0070062]; extracellular region [GO:0005576]; endopeptidase inhibitor activity [GO:0004866]; hyaluronic acid binding [GO:0005540]; serine-type endopeptidase inhibitor activity [GO:0004867]; hyaluronan metabolic process [GO:0030212]

Q96F07	CYFIP2	Cytoplasmic FMR1-interacting protein 2 OS	148.3	3.41	1.77	3.95E-02	1.40	activation of cysteine-type endopeptidase activity [GO:0097202]; apoptotic process [GO:0006915]; cell morphogenesis [GO:0000902]; cell-cell adhesion [GO:0098609]; dendrite extension [GO:0097484]; positive regulation of neurotrophin TRK receptor signaling pathway [GO:0051388]; positive regulation of proteolysis [GO:0045862]; regulation of actin filament polymerization [GO:0030833]	anchoring junction [GO:0070161]; cytoplasm [GO:0005737]; cytosol [GO:0005829]; extracellular exosome [GO:0070062]; membrane [GO:0016020]; neuron projection [GO:0043005]; nucleus [GO:0005634]; perinuclear region of cytoplasm [GO:0048471]; SCAR complex [GO:0031209]; synapse [GO:0045202]; small GTPase binding [GO:0031267]; activation of cysteine-type endopeptidase activity [GO:0097202]; apoptotic process [GO:0006915]; cell morphogenesis [GO:0000902]; cell-cell adhesion [GO:0098609]; dendrite extension [GO:0097484]; positive regulation of neurotrophin TRK receptor signaling pathway [GO:0051388]; positive regulation of proteolysis [GO:0045862]; regulation of actin filament polymerization [GO:0030833]
P15170	GSPT1	Eukaryotic peptide chain release factor GTP-binding subunit ERF3A OS	55.7	3.40	1.77	7.43E-02	1.13	G1/S transition of mitotic cell cycle [GO:0000082]; nuclear-transcribed mRNA catabolic process, nonsense-mediated decay [GO:0000184]; protein methylation [GO:0006479]; regulation of translational termination [GO:0006449]; translation [GO:0006412]; translational termination [GO:0006415]	cytoplasm [GO:0005737]; cytosol [GO:0005829]; translation release factor complex [GO:0018444]; GTP binding [GO:0005525]; GTPase activity [GO:0003924]; RNA binding [GO:0003723]; translation release factor activity [GO:0003747]; G1/S transition of mitotic cell cycle [GO:0000082]; nuclear-transcribed mRNA catabolic process, nonsense-mediated decay [GO:0000184]; protein methylation [GO:0006479]; regulation of translational termination [GO:0006449]; translation [GO:0006412]; translational termination [GO:0006415]
P25311	AZGP1	Zinc-alpha-2-glycoprotein OS	34.2	3.37	1.75	7.24E-02	1.14	antigen processing and presentation of endogenous peptide antigen via MHC class Ib [GO:0002476]; cell adhesion [GO:0007155]; detection of chemical stimulus involved in sensory perception of bitter taste [GO:0001580]; negative regulation of cell population proliferation [GO:0008285]; retina homeostasis [GO:0001895]	collagen-containing extracellular matrix [GO:0062023]; external side of plasma membrane [GO:0009897]; extracellular exosome [GO:0070062]; extracellular region [GO:0005576]; extracellular space [GO:0005615]; nucleus [GO:0005634]; protein transmembrane transporter activity [GO:0008320]; ribonuclease activity [GO:0004540]; antigen processing and presentation of endogenous peptide antigen via MHC class Ib [GO:0002476]; cell adhesion [GO:0007155]; detection of chemical stimulus involved in sensory perception of bitter taste [GO:0001580]; negative regulation of cell population

									proliferation [GO:0008285]; retina homeostasis [GO:0001895]
P02788	LTF	Lactotransferrin OS	78.1	3.36	1.75	1.14E-02	1.94	antibacterial humoral response [GO:0019731]; antifungal humoral response [GO:0019732]; antimicrobial humoral immune response mediated by antimicrobial peptide [GO:0061844]; bone morphogenesis [GO:0060349]; defense response to Gram-negative bacterium [GO:0050829]; defense response to Gram-positive bacterium [GO:0050830]; humoral immune response [GO:0006959]; innate immune response [GO:0045087]; innate immune response in mucosa [GO:0002227]; iron ion homeostasis [GO:0055072]; iron ion transport [GO:0006826]; killing of cells of another organism [GO:0031640]; membrane disruption in another organism [GO:0051673]; negative regulation by host of viral process [GO:0044793]; negative regulation of apoptotic process [GO:0043066]; negative regulation of ATP-dependent activity [GO:0032780]; negative regulation of cysteine-type endopeptidase activity [GO:2000117]; negative regulation of lipopolysaccharide-mediated signaling pathway [GO:0031665]; negative regulation of membrane potential [GO:0045837]; negative regulation of osteoclast development [GO:2001205]; negative regulation of single-species biofilm formation in or on host organism [GO:1900229]; negative regulation of tumor necrosis factor (ligand) superfamily member 11 production [GO:2000308]; negative regulation of viral genome replication [GO:0045071]; negative regulation of viral process [GO:0048525]; ossification [GO:0001503]; positive regulation of bone mineralization involved in bone	cell surface [GO:0009986]; cytoplasm [GO:0005737]; early endosome [GO:0005769]; extracellular exosome [GO:0070062]; extracellular region [GO:0005576]; extracellular space [GO:0005615]; nucleus [GO:0005634]; phagocytic vesicle lumen [GO:0097013]; plasma membrane [GO:0005886]; protein-containing complex [GO:0032991]; recycling endosome [GO:0055037]; secretory granule [GO:0030141]; specific granule [GO:0042581]; specific granule lumen [GO:0035580]; tertiary granule lumen [GO:1904724]; cysteine-type endopeptidase inhibitor activity [GO:0004869]; DNA binding [GO:0003677]; heparin binding [GO:0008201]; iron ion binding [GO:0005506]; lipopolysaccharide binding [GO:0001530]; protein serine/threonine kinase activator activity [GO:0043539]; serine-type endopeptidase activity [GO:0004252]; antibacterial humoral response [GO:0019731]; antifungal humoral response [GO:0019732]; antimicrobial humoral immune response mediated by antimicrobial peptide [GO:0061844]; bone morphogenesis [GO:0060349]; defense response to Gram-negative bacterium [GO:0050829]; defense response to Gram-positive bacterium [GO:0050830]; humoral immune response [GO:0006959]; innate immune response [GO:0045087]; innate immune response in mucosa [GO:0002227]; iron ion homeostasis [GO:0055072]; iron ion transport [GO:0006826]; killing of cells of another organism [GO:0031640]; membrane disruption in another organism [GO:0051673]; negative regulation by host of viral process [GO:0044793]; negative regulation of apoptotic process [GO:0043066]; negative regulation of ATP-dependent activity [GO:0032780]; negative regulation of cysteine-type endopeptidase activity [GO:2000117]; negative regulation of

								maturation [GO:1900159]; positive regulation of chondrocyte proliferation [GO:1902732]; positive regulation of I-kappaB kinase/NF-kappaB signaling [GO:0043123]; positive regulation of NF-kappaB transcription factor activity [GO:0051092]; positive regulation of osteoblast differentiation [GO:0045669]; positive regulation of osteoblast proliferation [GO:0033690]; positive regulation of protein serine/threonine kinase activity [GO:0071902]; positive regulation of toll-like receptor 4 signaling pathway [GO:0034145]; proteolysis [GO:0006508]; regulation of cytokine production [GO:0001817]; regulation of tumor necrosis factor production [GO:0032680]; retina homeostasis [GO:0001895]	lipopolysaccharide-mediated signaling pathway [GO:0031665]; negative regulation of membrane potential [GO:0045837]; negative regulation of osteoclast development [GO:2001205]; negative regulation of single-species biofilm formation in or on host organism [GO:1900229]; negative regulation of tumor necrosis factor (ligand) superfamily member 11 production [GO:2000308]; negative regulation of viral genome replication [GO:0045071]; negative regulation of viral process [GO:0048525]; ossification [GO:0001503]; positive regulation of bone mineralization involved in bone maturation [GO:1900159]; positive regulation of chondrocyte proliferation [GO:1902732]; positive regulation of I-kappaB kinase/NF-kappaB signaling [GO:0043123]; positive regulation of NF-kappaB transcription factor activity [GO:0051092]; positive regulation of osteoblast differentiation [GO:0045669]; positive regulation of osteoblast proliferation [GO:0033690]; positive regulation of protein serine/threonine kinase activity [GO:0071902]; positive regulation of toll-like receptor 4 signaling pathway [GO:0034145]; proteolysis [GO:0006508]; regulation of cytokine production [GO:0001817]; regulation of tumor necrosis factor production [GO:0032680]; retina homeostasis [GO:0001895]
Q6UWP8	SBSN	Suprabasin OS	60.5	3.32	1.73	8.54E-02	1.07		extracellular exosome [GO:0070062]

P02751	FN1	Fibronectin OS	272.2	3.30	1.72	6.13E-02	1.21	acute-phase response [GO:0006953]; angiogenesis [GO:0001525]; biological process involved in interaction with symbiont [GO:0051702]; blood coagulation, fibrin clot formation [GO:0072378]; calcium-independent cell-matrix adhesion [GO:0007161]; cell adhesion [GO:0007155]; cell-matrix adhesion [GO:0007160]; cell-substrate junction assembly [GO:0007044]; endodermal cell differentiation [GO:0035987]; heart development [GO:0007507]; integrin activation [GO:0033622]; integrin-mediated signaling pathway [GO:0007229]; negative regulation of monocyte activation [GO:0150102]; negative regulation of transforming growth factor beta production [GO:0071635]; nervous system development [GO:0007399]; neural crest cell migration involved in autonomic nervous system development [GO:1901166]; peptide cross-linking [GO:0018149]; platelet aggregation [GO:0070527]; positive regulation of axon extension [GO:0045773]; positive regulation of cell population proliferation [GO:0008284]; positive regulation of fibroblast proliferation [GO:0048146]; positive regulation of gene expression [GO:0010628]; positive regulation of phosphatidylinositol 3-kinase signaling [GO:0014068]; positive regulation of substrate-dependent cell migration, cell attachment to substrate [GO:1904237]; regulation of cell shape [GO:0008360]; regulation of ERK1 and ERK2 cascade [GO:0070372]; regulation of protein phosphorylation [GO:0001932]; response to wounding [GO:0009611]; substrate adhesion-dependent cell spreading [GO:0034446]	apical plasma membrane [GO:0016324]; basement membrane [GO:0005604]; blood microparticle [GO:0072562]; collagen-containing extracellular matrix [GO:0062023]; endoplasmic reticulum lumen [GO:0005788]; endoplasmic reticulum-Golgi intermediate compartment [GO:0005793]; extracellular exosome [GO:0070062]; extracellular matrix [GO:0031012]; extracellular region [GO:0005576]; extracellular space [GO:0005615]; fibrinogen complex [GO:0005577]; plasma membrane [GO:0005886]; platelet alpha granule lumen [GO:0031093]; chaperone binding [GO:0051087]; collagen binding [GO:0005518]; disordered domain specific binding [GO:0097718]; enzyme binding [GO:0019899]; extracellular matrix structural constituent [GO:0005201]; heparin binding [GO:0008201]; identical protein binding [GO:0042802]; integrin binding [GO:0005178]; peptidase activator activity [GO:0016504]; protease binding [GO:0002020]; protein C-terminus binding [GO:0008022]; proteoglycan binding [GO:0043394]; signaling receptor binding [GO:0005102]; acute-phase response [GO:0006953]; angiogenesis [GO:0001525]; biological process involved in interaction with symbiont [GO:0051702]; blood coagulation, fibrin clot formation [GO:0072378]; calcium-independent cell-matrix adhesion [GO:0007161]; cell adhesion [GO:0007155]; cell-matrix adhesion [GO:0007160]; cell-substrate junction assembly [GO:0007044]; endodermal cell differentiation [GO:0035987]; heart development [GO:0007507]; integrin activation [GO:0033622]; integrin-mediated signaling pathway [GO:0007229]; negative regulation of monocyte activation [GO:0150102]; negative regulation of transforming growth factor beta production [GO:0071635]; nervous system development [GO:0007399]; neural crest cell migration involved in autonomic nervous system development [GO:1901166]; peptide cross-linking [GO:0018149]; platelet aggregation [GO:0070527]; positive regulation of axon extension

									[GO:0045773]; positive regulation of cell population proliferation [GO:0008284]; positive regulation of fibroblast proliferation [GO:0048146]; positive regulation of gene expression [GO:0010628]; positive regulation of phosphatidylinositol 3-kinase signaling [GO:0014068]; positive regulation of substrate-dependent cell migration, cell attachment to substrate [GO:1904237]; regulation of cell shape [GO:0008360]; regulation of ERK1 and ERK2 cascade [GO:0070372]; regulation of protein phosphorylation [GO:0001932]; response to wounding [GO:0009611]; substrate adhesion-dependent cell spreading [GO:0034446]
P07195	LDHB	L-lactate dehydrogenase B chain OS	36.6	3.29	1.72	5.87E-02	1.23	lactate metabolic process [GO:0006089]; NAD metabolic process [GO:0019674]; pyruvate metabolic process [GO:0006090]	cytoplasm [GO:0005737]; cytosol [GO:0005829]; extracellular exosome [GO:0070062]; membrane [GO:0016020]; membrane raft [GO:0045121]; mitochondrial inner membrane [GO:0005743]; oxidoreductase complex [GO:1990204]; identical protein binding [GO:0042802]; kinase binding [GO:0019900]; L-lactate dehydrogenase activity [GO:0004459]; NAD binding [GO:0051287]; lactate metabolic process [GO:0006089]; NAD metabolic process [GO:0019674]; pyruvate metabolic process [GO:0006090]
Q9NPH3	IL1RAP	Interleukin-1 receptor accessory protein OS	65.4	3.26	1.71	7.43E-02	1.13	immune response [GO:0006955]; inflammatory response [GO:0006954]; innate immune response [GO:0045087]; positive regulation of interleukin-13 production [GO:0032736]; positive regulation of interleukin-4 production [GO:0032753]; positive regulation of interleukin-5 production [GO:0032754]; positive regulation of interleukin-6 production [GO:0032755]; positive regulation of NF-kappaB transcription factor activity [GO:0051092]; positive regulation of synapse assembly [GO:0051965]; protein-containing complex assembly [GO:0065003]; regulation of	extracellular region [GO:0005576]; glutamatergic synapse [GO:0098978]; integral component of plasma membrane [GO:0005887]; membrane [GO:0016020]; plasma membrane [GO:0005886]; interleukin-1 receptor activity [GO:0004908]; interleukin-1 receptor binding [GO:0005149]; interleukin-33 receptor activity [GO:0002114]; NAD(P)+ nucleosidase activity [GO:0050135]; NAD+ nucleotidase, cyclic ADP-ribose generating [GO:0061809]; immune response [GO:0006955]; inflammatory response [GO:0006954]; innate immune response [GO:0045087]; positive regulation of interleukin-13 production [GO:0032736]; positive regulation of interleukin-4 production [GO:0032753];

								postsynaptic density assembly [GO:0099151]; regulation of presynapse assembly [GO:1905606]; synaptic membrane adhesion [GO:0099560]; trans-synaptic signaling by trans-synaptic complex [GO:0099545]	positive regulation of interleukin-5 production [GO:0032754]; positive regulation of interleukin-6 production [GO:0032755]; positive regulation of NF-kappaB transcription factor activity [GO:0051092]; positive regulation of synapse assembly [GO:0051965]; protein-containing complex assembly [GO:0065003]; regulation of postsynaptic density assembly [GO:0099151]; regulation of presynapse assembly [GO:1905606]; synaptic membrane adhesion [GO:0099560]; trans-synaptic signaling by trans-synaptic complex [GO:0099545]
P15924	DSP	Desmoplakin OS	331.6	3.25	1.70	1.39E-02	1.86	adherens junction organization [GO:0034332]; bundle of His cell-Purkinje myocyte adhesion involved in cell communication [GO:0086073]; cell-cell adhesion [GO:0098609]; desmosome organization [GO:0002934]; epidermis development [GO:0008544]; epithelial cell-cell adhesion [GO:0090136]; intermediate filament cytoskeleton organization [GO:0045104]; intermediate filament organization [GO:0045109]; keratinocyte differentiation [GO:0030216]; peptide cross-linking [GO:0018149]; protein localization to cell-cell junction [GO:0150105]; regulation of heart rate by cardiac conduction [GO:0086091]; regulation of ventricular cardiac muscle cell action potential [GO:0098911]; skin development [GO:0043588]; ventricular compact myocardium morphogenesis [GO:0003223]; wound healing [GO:0042060]	basolateral plasma membrane [GO:0016323]; cornified envelope [GO:0001533]; cytoplasm [GO:0005737]; desmosome [GO:0030057]; extracellular exosome [GO:0070062]; fascia adherens [GO:0005916]; ficolin-1-rich granule membrane [GO:0101003]; intercalated disc [GO:0014704]; intermediate filament [GO:0005882]; membrane [GO:0016020]; nucleus [GO:0005634]; plasma membrane [GO:0005886]; cell adhesive protein binding involved in bundle of His cell-Purkinje myocyte communication [GO:0086083]; protein kinase C binding [GO:0005080]; RNA binding [GO:0003723]; scaffold protein binding [GO:0097110]; structural constituent of cytoskeleton [GO:0005200]; structural molecule activity [GO:0005198]; adherens junction organization [GO:0034332]; bundle of His cell-Purkinje myocyte adhesion involved in cell communication [GO:0086073]; cell-cell adhesion [GO:0098609]; desmosome organization [GO:0002934]; epidermis development [GO:0008544]; epithelial cell-cell adhesion [GO:0090136]; intermediate filament cytoskeleton organization [GO:0045104]; intermediate filament organization [GO:0045109]; keratinocyte differentiation [GO:0030216]; peptide cross-linking [GO:0018149]; protein localization to cell-cell junction [GO:0150105]; regulation of heart rate by cardiac conduction [GO:0086091]; regulation of ventricular cardiac muscle cell action potential [GO:0098911]; skin development [GO:0043588];

									ventricular compact myocardium morphogenesis [GO:0003223]; wound healing [GO:0042060]
Q8IY22	CMIP	C-Maf-inducing protein OS	86.3	3.20	1.68	1.53E-02	1.81	in utero embryonic development [GO:0001701]	cytosol [GO:0005829]; nucleoplasm [GO:0005654]; in utero embryonic development [GO:0001701]
P01040	CSTA	Cystatin-A OS	11	3.05	1.61	2.57E-02	1.59	cell-cell adhesion [GO:0098609]; keratinocyte differentiation [GO:0030216]; negative regulation of peptidase activity [GO:0010466]; negative regulation of proteolysis [GO:0045861]; peptide cross-linking [GO:0018149]	cornified envelope [GO:0001533]; cytoplasm [GO:0005737]; cytosol [GO:0005829]; extracellular space [GO:0005615]; nucleoplasm [GO:0005654]; peptidase inhibitor complex [GO:1904090]; cysteine-type endopeptidase inhibitor activity [GO:0004869]; protease binding [GO:0002020]; cell-cell adhesion [GO:0098609]; keratinocyte differentiation [GO:0030216]; negative regulation of peptidase activity [GO:0010466]; negative regulation of proteolysis [GO:0045861]; peptide cross-linking [GO:0018149]
Q8IYH5	ZZZ3	ZZ-type zinc finger-containing protein 3 OS	102	3.04	1.60	2.07E-02	1.68	histone H3 acetylation [GO:0043966]; histone H3-K14 acetylation [GO:0044154]; histone H4 acetylation [GO:0043967]; regulation of cell cycle [GO:0051726]; regulation of cell division [GO:0051302]; regulation of embryonic development [GO:0045995]; regulation of histone deacetylation [GO:0031063]; regulation of transcription by RNA polymerase II [GO:0006357]; regulation of transcription, DNA-templated [GO:0006355]; regulation of tubulin deacetylation [GO:0090043]	ATAC complex [GO:0140672]; mitotic spindle [GO:0072686]; nucleolus [GO:0005730]; nucleoplasm [GO:0005654]; DNA binding [GO:0003677]; lysine-acetylated histone binding [GO:0070577]; methylated histone binding [GO:0035064]; zinc ion binding [GO:0008270]; histone H3 acetylation [GO:0043966]; histone H3-K14 acetylation [GO:0044154]; histone H4 acetylation [GO:0043967]; regulation of cell cycle [GO:0051726]; regulation of cell division [GO:0051302]; regulation of embryonic development [GO:0045995]; regulation of histone deacetylation [GO:0031063]; regulation of transcription by RNA polymerase II [GO:0006357]; regulation of transcription, DNA-templated [GO:0006355]; regulation of tubulin deacetylation [GO:0090043]

Q16543	CDC37	Hsp90 co-chaperone Cdc37 OS	44.4	3.01	1.59	8.72E-02	1.06	positive regulation of mitophagy in response to mitochondrial depolarization [GO:0098779]; post-transcriptional regulation of gene expression [GO:0010608]; protein folding [GO:0006457]; protein stabilization [GO:0050821]; protein targeting [GO:0006605]; regulation of cyclin-dependent protein serine/threonine kinase activity [GO:0000079]; regulation of interferon-gamma-mediated signaling pathway [GO:0060334]; regulation of type I interferon-mediated signaling pathway [GO:0060338]	chaperone complex [GO:0101031]; cytoplasm [GO:0005737]; cytosol [GO:0005829]; extracellular exosome [GO:0070062]; HSP90-CDC37 chaperone complex [GO:1990565]; chaperone binding [GO:0051087]; heat shock protein binding [GO:0031072]; Hsp90 protein binding [GO:0051879]; kinase binding [GO:0019900]; protein kinase binding [GO:0019901]; protein kinase regulator activity [GO:0019887]; scaffold protein binding [GO:0097110]; unfolded protein binding [GO:0051082]; positive regulation of mitophagy in response to mitochondrial depolarization [GO:0098779]; post-transcriptional regulation of gene expression [GO:0010608]; protein folding [GO:0006457]; protein stabilization [GO:0050821]; protein targeting [GO:0006605]; regulation of cyclin-dependent protein serine/threonine kinase activity [GO:0000079]; regulation of interferon-gamma-mediated signaling pathway [GO:0060334]; regulation of type I interferon-mediated signaling pathway [GO:0060338]
Q02413	DSG1	Desmoglein-1 OS	113.7	2.98	1.57	2.34E-02	1.63	calcium-dependent cell-cell adhesion via plasma membrane cell adhesion molecules [GO:0016339]; cell-cell adhesion [GO:0098609]; cell-cell junction assembly [GO:0007043]; homophilic cell adhesion via plasma membrane adhesion molecules [GO:0007156]; maternal process involved in female pregnancy [GO:0060135]; protein stabilization [GO:0050821]; response to progesterone [GO:0032570]	apical plasma membrane [GO:0016324]; cornified envelope [GO:0001533]; cytoplasmic side of plasma membrane [GO:0009898]; cytosol [GO:0005829]; desmosome [GO:0030057]; ficolin-1-rich granule membrane [GO:0101003]; integral component of membrane [GO:0016021]; lateral plasma membrane [GO:0016328]; plasma membrane [GO:0005886]; calcium ion binding [GO:0005509]; gamma-catenin binding [GO:0045295]; toxic substance binding [GO:0015643]; calcium-dependent cell-cell adhesion via plasma membrane cell adhesion molecules [GO:0016339]; cell-cell adhesion [GO:0098609]; cell-cell junction assembly [GO:0007043]; homophilic cell adhesion via plasma membrane adhesion molecules [GO:0007156]; maternal process involved in female pregnancy [GO:0060135]; protein stabilization [GO:0050821]; response to progesterone [GO:0032570]

Q92542	NCSTN	Nicastrin OS	78.4	2.95	1.56	2.44E-02	1.61	adult behavior [GO:0030534]; amyloid precursor protein biosynthetic process [GO:0042983]; amyloid precursor protein catabolic process [GO:0042987]; amyloid precursor protein metabolic process [GO:0042982]; amyloid-beta formation [GO:0034205]; cellular response to calcium ion [GO:0071277]; central nervous system myelination [GO:0022010]; cerebellum development [GO:0021549]; dopamine receptor signaling pathway [GO:0007212]; epithelial cell proliferation [GO:0050673]; glutamate receptor signaling pathway [GO:0007215]; learning or memory [GO:0007611]; membrane protein ectodomain proteolysis [GO:0006509]; membrane protein intracellular domain proteolysis [GO:0031293]; myeloid cell homeostasis [GO:0002262]; neuron apoptotic process [GO:0051402]; Notch receptor processing [GO:0007220]; Notch signaling pathway [GO:0007219]; positive regulation of amyloid precursor protein biosynthetic process [GO:0042986]; positive regulation of catalytic activity [GO:0043085]; positive regulation of endopeptidase activity [GO:0010950]; protein processing [GO:0016485]; proteolysis [GO:0006508]; regulation of long-term synaptic potentiation [GO:1900271]; short-term synaptic potentiation [GO:1990926]; T cell proliferation [GO:0042098]	azurophil granule membrane [GO:0035577]; early endosome [GO:0005769]; endoplasmic reticulum [GO:0005783]; endoplasmic reticulum membrane [GO:0005789]; endosome membrane [GO:0010008]; extracellular exosome [GO:0070062]; focal adhesion [GO:0005925]; gamma-secretase complex [GO:0070765]; Golgi apparatus [GO:0005794]; Golgi membrane [GO:0000139]; integral component of membrane [GO:0016021]; integral component of plasma membrane [GO:0005887]; integral component of presynaptic membrane [GO:0099056]; lysosomal membrane [GO:0005765]; melanosome [GO:0042470]; membrane [GO:0016020]; mitochondrion [GO:0005739]; plasma membrane [GO:0005886]; sarcolemma [GO:0042383]; synaptic vesicle [GO:0008021]; aspartic endopeptidase activity, intramembrane cleaving [GO:0042500]; ATPase binding [GO:0051117]; growth factor receptor binding [GO:0070851]; protein-macromolecule adaptor activity [GO:0030674]; adult behavior [GO:0030534]; amyloid precursor protein biosynthetic process [GO:0042983]; amyloid precursor protein catabolic process [GO:0042987]; amyloid precursor protein metabolic process [GO:0042982]; amyloid-beta formation [GO:0034205]; cellular response to calcium ion [GO:0071277]; central nervous system myelination [GO:0022010]; cerebellum development [GO:0021549]; dopamine receptor signaling pathway [GO:0007212]; epithelial cell proliferation [GO:0050673]; glutamate receptor signaling pathway [GO:0007215]; learning or memory [GO:0007611]; membrane protein ectodomain proteolysis [GO:0006509]; membrane protein intracellular domain proteolysis [GO:0031293]; myeloid cell homeostasis [GO:0002262]; neuron apoptotic process [GO:0051402]; Notch receptor processing [GO:0007220]; Notch signaling pathway [GO:0007219]; positive regulation of amyloid precursor protein biosynthetic process [GO:0042986]; positive regulation of catalytic activity

									[GO:0043085]; positive regulation of endopeptidase activity [GO:0010950]; protein processing [GO:0016485]; proteolysis [GO:0006508]; regulation of long-term synaptic potentiation [GO:1900271]; short-term synaptic potentiation [GO:1990926]; T cell proliferation [GO:0042098]
P01023	A2M	Alpha-2-macroglobulin OS	163.2	2.86	1.52	2.88E-02	1.54	acute inflammatory response to antigenic stimulus [GO:0002438]; acute-phase response [GO:0006953]; embryonic liver development [GO:1990402]; luteinization [GO:0001553]; negative regulation of complement activation, lectin pathway [GO:0001869]; response to carbon dioxide [GO:0010037]; response to glucocorticoid [GO:0051384]; response to nutrient [GO:0007584]; response to prostaglandin E [GO:0034695]; stem cell differentiation [GO:0048863]	blood microparticle [GO:0072562]; collagen-containing extracellular matrix [GO:0062023]; extracellular exosome [GO:0070062]; extracellular region [GO:0005576]; extracellular space [GO:0005615]; platelet alpha granule lumen [GO:0031093]; brain-derived neurotrophic factor binding [GO:0048403]; calcium-dependent protein binding [GO:0048306]; endopeptidase inhibitor activity [GO:0004866]; enzyme binding [GO:0019899]; growth factor binding [GO:0019838]; identical protein binding [GO:0042802]; interleukin-1 binding [GO:0019966]; interleukin-8 binding [GO:0019959]; nerve growth factor binding [GO:0048406]; protease binding [GO:0002020]; serine-type endopeptidase inhibitor activity [GO:0004867]; signaling receptor binding [GO:0005102]; tumor necrosis factor binding [GO:0043120]; acute inflammatory response to antigenic stimulus [GO:0002438]; acute-phase response [GO:0006953]; embryonic liver development [GO:1990402]; luteinization [GO:0001553]; negative regulation of complement activation, lectin pathway [GO:0001869]; response to carbon dioxide [GO:0010037]; response to glucocorticoid [GO:0051384]; response to nutrient [GO:0007584]; response to prostaglandin E

									[GO:0034695]; stem cell differentiation [GO:0048863]
Q9NZW5	PALS2	Protein PALS2 OS	61.1	2.86	1.51	2.90E-02	1.54	protein-containing complex assembly [GO:0065003]	cell-cell junction [GO:0005911]; extracellular exosome [GO:0070062]; membrane [GO:0016020]; plasma membrane [GO:0005886]; protein-containing complex assembly [GO:0065003]
P05089	ARG1	Arginase-1 OS	34.7	2.84	1.51	2.99E-02	1.52	adaptive immune response [GO:0002250]; aging [GO:0007568]; arginine catabolic process [GO:0006527]; arginine catabolic process to ornithine [GO:0019547]; cellular response to dexamethasone stimulus [GO:0071549]; cellular response to glucagon stimulus [GO:0071377]; cellular response to hydrogen peroxide [GO:0070301]; cellular response to interleukin-4 [GO:0071353]; cellular response to lipopolysaccharide [GO:0071222]; cellular response to transforming growth factor beta stimulus [GO:0071560]; collagen biosynthetic process [GO:0032964]; defense response to protozoan [GO:0042832]; innate immune response [GO:0045087]; liver development [GO:0001889]; lung development [GO:0030324]; mammary gland involution [GO:0060056]; maternal process involved in female pregnancy [GO:0060135]; negative regulation of activated T cell proliferation [GO:0046007]; negative regulation of interferon-gamma-mediated signaling pathway [GO:0060336]; negative regulation of T cell proliferation [GO:0042130]; negative regulation of T-helper 2 cell cytokine production [GO:2000552]; positive regulation of endothelial cell proliferation [GO:0001938]; positive regulation of	azurophil granule lumen [GO:0035578]; cytoplasm [GO:0005737]; cytosol [GO:0005829]; extracellular region [GO:0005576]; extracellular space [GO:0005615]; mitochondrial outer membrane [GO:0005741]; neuron projection [GO:0043005]; neuronal cell body [GO:0043025]; nucleus [GO:0005634]; specific granule lumen [GO:0035580]; arginase activity [GO:0004053]; identical protein binding [GO:0042802]; manganese ion binding [GO:0030145]; adaptive immune response [GO:0002250]; aging [GO:0007568]; arginine catabolic process [GO:0006527]; arginine catabolic process to ornithine [GO:0019547]; cellular response to dexamethasone stimulus [GO:0071549]; cellular response to glucagon stimulus [GO:0071377]; cellular response to hydrogen peroxide [GO:0070301]; cellular response to interleukin-4 [GO:0071353]; cellular response to lipopolysaccharide [GO:0071222]; cellular response to transforming growth factor beta stimulus [GO:0071560]; collagen biosynthetic process [GO:0032964]; defense response to protozoan [GO:0042832]; innate immune response [GO:0045087]; liver development [GO:0001889]; lung development [GO:0030324]; mammary gland involution [GO:0060056]; maternal process involved in female pregnancy [GO:0060135]; negative regulation of activated T cell proliferation [GO:0046007]; negative regulation of interferon-

								neutrophil mediated killing of fungus [GO:0070965]; regulation of L-arginine import across plasma membrane [GO:1905541]; response to amine [GO:0014075]; response to amino acid [GO:0043200]; response to axon injury [GO:0048678]; response to cadmium ion [GO:0046686]; response to herbicide [GO:0009635]; response to manganese ion [GO:0010042]; response to methylmercury [GO:0051597]; response to selenium ion [GO:0010269]; response to vitamin A [GO:0033189]; response to vitamin E [GO:0033197]; response to xenobiotic stimulus [GO:0009410]; response to zinc ion [GO:0010043]; urea cycle [GO:0000050]	gamma-mediated signaling pathway [GO:0060336]; negative regulation of T cell proliferation [GO:0042130]; negative regulation of T-helper 2 cell cytokine production [GO:2000552]; positive regulation of endothelial cell proliferation [GO:0001938]; positive regulation of neutrophil mediated killing of fungus [GO:0070965]; regulation of L-arginine import across plasma membrane [GO:1905541]; response to amine [GO:0014075]; response to amino acid [GO:0043200]; response to axon injury [GO:0048678]; response to cadmium ion [GO:0046686]; response to herbicide [GO:0009635]; response to manganese ion [GO:0010042]; response to methylmercury [GO:0051597]; response to selenium ion [GO:0010269]; response to vitamin A [GO:0033189]; response to vitamin E [GO:0033197]; response to xenobiotic stimulus [GO:0009410]; response to zinc ion [GO:0010043]; urea cycle [GO:0000050]
P06756	ITGAV	Integrin alpha-V OS	116	2.81	1.49	3.04E-02	1.52	angiogenesis [GO:0001525]; apolipoprotein A-I-mediated signaling pathway [GO:0038027]; apoptotic cell clearance [GO:0043277]; calcium ion transmembrane transport [GO:0070588]; cell adhesion [GO:0007155]; cell adhesion mediated by integrin [GO:0033627]; cell migration [GO:0016477]; cell-cell adhesion [GO:0098609]; cell-matrix adhesion [GO:0007160]; cell-substrate adhesion [GO:0031589]; endodermal cell differentiation [GO:0035987]; entry into host cell by a symbiont-containing vacuole [GO:0085017]; ERK1 and ERK2 cascade [GO:0070371]; extrinsic apoptotic signaling pathway in absence of ligand [GO:0097192]; heterotypic cell-cell adhesion [GO:0034113]; integrin-mediated signaling pathway [GO:0007229]; negative chemotaxis [GO:0050919]; negative regulation of entry of bacterium into host cell [GO:2000536]; negative regulation of extrinsic apoptotic	alphav-beta3 integrin-HMGB1 complex [GO:0035868]; alphav-beta3 integrin-IGF-1-IGF1R complex [GO:0035867]; alphav-beta3 integrin-PKCalpha complex [GO:0035866]; cell surface [GO:0009986]; cytosol [GO:0005829]; external side of plasma membrane [GO:0009897]; extracellular exosome [GO:0070062]; filopodium membrane [GO:0031527]; focal adhesion [GO:0005925]; integral component of plasma membrane [GO:0005887]; integrin alphav-beta3 complex [GO:0034683]; integrin alphav-beta5 complex [GO:0034684]; integrin alphav-beta6 complex [GO:0034685]; integrin alphav-beta8 complex [GO:0034686]; integrin complex [GO:0008305]; lamellipodium membrane [GO:0031258]; membrane [GO:0016020]; microvillus membrane [GO:0031528]; phagocytic vesicle [GO:0045335]; plasma membrane [GO:0005886]; ruffle membrane [GO:0032587]; specific granule membrane [GO:0035579]; coreceptor activity [GO:0015026]; extracellular matrix binding [GO:0050840]; extracellular matrix protein binding [GO:1990430]; fibronectin binding

								signaling pathway [GO:2001237]; negative regulation of lipid storage [GO:0010888]; negative regulation of lipid transport [GO:0032369]; negative regulation of lipoprotein metabolic process [GO:0050748]; negative regulation of low-density lipoprotein receptor activity [GO:1905598]; negative regulation of macrophage derived foam cell differentiation [GO:0010745]; positive regulation of cell adhesion [GO:0045785]; positive regulation of cell migration [GO:0030335]; positive regulation of cell population proliferation [GO:0008284]; positive regulation of cytosolic calcium ion concentration [GO:0007204]; positive regulation of intracellular signal transduction [GO:1902533]; positive regulation of osteoblast proliferation [GO:0033690]; positive regulation of small GTPase mediated signal transduction [GO:0051057]; regulation of phagocytosis [GO:0050764]; regulation of transforming growth factor beta activation [GO:1901388]; substrate adhesion-dependent cell spreading [GO:0034446]; transforming growth factor beta production [GO:0071604]; vasculogenesis [GO:0001570]; viral entry into host cell [GO:0046718]	[GO:0001968]; integrin binding [GO:0005178]; metal ion binding [GO:0046872]; opsonin binding [GO:0001846]; protease binding [GO:0002020]; protein kinase C binding [GO:0005080]; transforming growth factor beta binding [GO:0050431]; virus receptor activity [GO:0001618]; voltage-gated calcium channel activity [GO:0005245]; angiogenesis [GO:0001525]; apolipoprotein A-I-mediated signaling pathway [GO:0038027]; apoptotic cell clearance [GO:0043277]; calcium ion transmembrane transport [GO:0070588]; cell adhesion [GO:0007155]; cell adhesion mediated by integrin [GO:0033627]; cell migration [GO:0016477]; cell-cell adhesion [GO:0098609]; cell-matrix adhesion [GO:0007160]; cell-substrate adhesion [GO:0031589]; endodermal cell differentiation [GO:0035987]; entry into host cell by a symbiont-containing vacuole [GO:0085017]; ERK1 and ERK2 cascade [GO:0070371]; extrinsic apoptotic signaling pathway in absence of ligand [GO:0097192]; heterotypic cell-cell adhesion [GO:0034113]; integrin-mediated signaling pathway [GO:0007229]; negative chemotaxis [GO:0050919]; negative regulation of entry of bacterium into host cell [GO:2000536]; negative regulation of extrinsic apoptotic signaling pathway [GO:2001237]; negative regulation of lipid storage [GO:0010888]; negative regulation of lipid transport [GO:0032369]; negative regulation of lipoprotein metabolic process [GO:0050748]; negative regulation of low-density lipoprotein receptor activity [GO:1905598]; negative regulation of macrophage derived foam cell differentiation [GO:0010745]; positive regulation of cell adhesion [GO:0045785]; positive regulation of cell migration [GO:0030335]; positive regulation of cell population proliferation [GO:0008284]; positive regulation of cytosolic calcium ion concentration [GO:0007204]; positive regulation of intracellular signal transduction [GO:1902533]; positive regulation of osteoblast proliferation [GO:0033690]; positive regulation of small GTPase mediated signal transduction [GO:0051057]; regulation of

									phagocytosis [GO:0050764]; regulation of transforming growth factor beta activation [GO:1901388]; substrate adhesion-dependent cell spreading [GO:0034446]; transforming growth factor beta production [GO:0071604]; vasculogenesis [GO:0001570]; viral entry into host cell [GO:0046718]
O75362	ZNF217	Zinc finger protein 217 OS	115.2	2.67	1.42	5.68E-02	1.25	negative regulation of transcription by RNA polymerase II [GO:0000122]; negative regulation of transcription, DNA-templated [GO:0045892]; regulation of transcription, DNA-templated [GO:0006355]	histone deacetylase complex [GO:0000118]; mitochondrion [GO:0005739]; nuclear speck [GO:0016607]; nucleoplasm [GO:0005654]; nucleus [GO:0005634]; DNA-binding transcription factor activity [GO:0003700]; DNA-binding transcription factor activity, RNA polymerase II-specific [GO:0000981]; DNA-binding transcription repressor activity, RNA polymerase II-specific [GO:0001227]; metal ion binding [GO:0046872]; RNA polymerase II cis-regulatory region sequence-specific DNA binding [GO:0000978]; transcription cis-regulatory region binding [GO:0000976]; negative regulation of transcription by RNA polymerase II [GO:0000122]; negative regulation of transcription, DNA-templated [GO:0045892]; regulation of transcription, DNA-templated [GO:0006355]

P15121	AKR1B1	Aldo-keto reductase family 1 member B1 OS	35.8	2.64	1.40	4.34E-02	1.36	C21-steroid hormone biosynthetic process [GO:0006700]; carbohydrate metabolic process [GO:0005975]; cellular hyperosmotic salinity response [GO:0071475]; daunorubicin metabolic process [GO:0044597]; doxorubicin metabolic process [GO:0044598]; epithelial cell maturation [GO:0002070]; fructose biosynthetic process [GO:0046370]; metanephric collecting duct development [GO:0072205]; negative regulation of apoptotic process [GO:0043066]; regulation of urine volume [GO:0035809]; renal water homeostasis [GO:0003091]; retinoid metabolic process [GO:0001523]	cytosol [GO:0005829]; extracellular exosome [GO:0070062]; extracellular space [GO:0005615]; nucleoplasm [GO:0005654]; alditol:NADP+ 1-oxidoreductase activity [GO:0004032]; allyl-alcohol dehydrogenase activity [GO:0047655]; electron transfer activity [GO:0009055]; glyceraldehyde oxidoreductase activity [GO:0043795]; glycerol dehydrogenase [NADP+] activity [GO:0047956]; NADP-retinol dehydrogenase activity [GO:0052650]; prostaglandin H2 endoperoxidase reductase activity [GO:0036130]; retinal dehydrogenase activity [GO:0001758]; C21-steroid hormone biosynthetic process [GO:0006700]; carbohydrate metabolic process [GO:0005975]; cellular hyperosmotic salinity response [GO:0071475]; daunorubicin metabolic process [GO:0044597]; doxorubicin metabolic process [GO:0044598]; epithelial cell maturation [GO:0002070]; fructose biosynthetic process [GO:0046370]; metanephric collecting duct development [GO:0072205]; negative regulation of apoptotic process [GO:0043066]; regulation of urine volume [GO:0035809]; renal water homeostasis [GO:0003091]; retinoid metabolic process [GO:0001523]
Q6ULP2	AFTPH	Aftiphilin OS	102.1	2.64	1.40	4.34E-02	1.36	intracellular transport [GO:0046907]; protein transport [GO:0015031]	AP-1 adaptor complex [GO:0030121]; cytosol [GO:0005829]; Golgi apparatus [GO:0005794]; intracellular membrane-bounded organelle [GO:0043231]; nucleoplasm [GO:0005654]; perinuclear region of cytoplasm [GO:0048471]; trans-Golgi network membrane [GO:0032588]; clathrin binding [GO:0030276]; intracellular transport [GO:0046907]; protein transport [GO:0015031]

P12814	ACTN1	Alpha-actinin-1 OS	103	2.63	1.39	4.43E-02	1.35	actin cytoskeleton organization [GO:0030036]; actin filament bundle assembly [GO:0051017]; actin filament network formation [GO:0051639]; actin filament organization [GO:0007015]; focal adhesion assembly [GO:0048041]; muscle cell development [GO:0055001]; platelet formation [GO:0030220]; platelet morphogenesis [GO:0036344]; regulation of apoptotic process [GO:0042981]	brush border [GO:0005903]; cell junction [GO:0030054]; cell projection [GO:0042995]; cell-cell junction [GO:0005911]; cortical actin cytoskeleton [GO:0030864]; cytoplasm [GO:0005737]; cytosol [GO:0005829]; dendritic spine [GO:0043197]; extracellular exosome [GO:0070062]; extracellular region [GO:0005576]; extracellular space [GO:0005615]; fascia adherens [GO:0005916]; focal adhesion [GO:0005925]; glutamatergic synapse [GO:0098978]; nucleus [GO:0005634]; plasma membrane [GO:0005886]; platelet alpha granule lumen [GO:0031093]; pseudopodium [GO:0031143]; ruffle [GO:0001726]; stress fiber [GO:0001725]; Z disc [GO:0030018]; actin filament binding [GO:0051015]; calcium ion binding [GO:0005509]; cytoskeletal regulatory protein binding [GO:0005519]; double-stranded RNA binding [GO:0003725]; integrin binding [GO:0005178]; nuclear receptor coactivator activity [GO:0030374]; protein domain specific binding [GO:0019904]; protein homodimerization activity [GO:0042803]; structural constituent of postsynapse [GO:0099186]; transmembrane transporter binding [GO:0044325]; vinculin binding [GO:0017166]; actin cytoskeleton organization [GO:0030036]; actin filament bundle assembly [GO:0051017]; actin filament network formation [GO:0051639]; actin filament organization [GO:0007015]; focal adhesion assembly [GO:0048041]; muscle cell development [GO:0055001]; platelet formation [GO:0030220]; platelet morphogenesis [GO:0036344]; regulation of apoptotic process [GO:0042981]
Q9NP66	HMG20A	High mobility group protein 20A OS	40.1	2.56	1.36	5.08E-02	1.29	chromatin organization [GO:0006325]; negative regulation of neuron differentiation [GO:0045665]; negative regulation of protein sumoylation [GO:0033234]; negative regulation of transcription by RNA polymerase II [GO:0000122]; regulation of gene expression [GO:0010468]; regulation of transcription, DNA-templated [GO:0006355]	nucleus [GO:0005634]; DNA binding [GO:0003677]; identical protein binding [GO:0042802]; chromatin organization [GO:0006325]; negative regulation of neuron differentiation [GO:0045665]; negative regulation of protein sumoylation [GO:0033234]; negative regulation of transcription by RNA polymerase II [GO:0000122]; regulation of gene

									expression [GO:0010468]; regulation of transcription, DNA-templated [GO:0006355]
Q8TEX9	IPO4	Importin-4 OS	118.6	2.53	1.34	5.37E-02	1.27	DNA replication-dependent chromatin assembly [GO:0006335]; DNA replication-independent chromatin assembly [GO:0006336]; protein import into nucleus [GO:0006606]	chromatin [GO:0000785]; cytoplasm [GO:0005737]; membrane [GO:0016020]; nucleus [GO:0005634]; protein-containing complex [GO:0032991]; nuclear import signal receptor activity [GO:0061608]; nuclear localization sequence binding [GO:0008139]; small GTPase binding [GO:0031267]; DNA replication-dependent chromatin assembly [GO:0006335]; DNA replication-independent chromatin assembly [GO:0006336]; protein import into nucleus [GO:0006606]
Q12797	ASPH	Aspartyl/asparaginyl beta-hydroxylase OS	85.8	2.50	1.32	5.68E-02	1.25	activation of cysteine-type endopeptidase activity [GO:0097202]; activation of store-operated calcium channel activity [GO:0032237]; calcium ion homeostasis [GO:0055074]; calcium ion transmembrane transport [GO:0070588]; cell population proliferation [GO:0008283]; cellular response to calcium ion [GO:0071277]; detection of calcium ion [GO:0005513]; face morphogenesis [GO:0060325]; limb morphogenesis [GO:0035108]; muscle contraction [GO:0006936]; negative regulation of cell population proliferation [GO:0008285]; pattern specification process [GO:0007389]; peptidyl-aspartic acid hydroxylation [GO:0042264]; positive regulation of calcium ion transport into cytosol [GO:0010524]; positive regulation of intracellular protein transport [GO:0090316]; positive regulation of proteolysis [GO:0045862]; positive regulation of ryanodine-sensitive calcium-release channel activity [GO:0060316]; positive regulation of transcription, DNA-templated [GO:0045893]; regulation of cardiac muscle contraction by regulation of the release of sequestered calcium ion [GO:0010881]; regulation of cell	calcium channel complex [GO:0034704]; cortical endoplasmic reticulum [GO:0032541]; endoplasmic reticulum [GO:0005783]; endoplasmic reticulum membrane [GO:0005789]; integral component of endoplasmic reticulum membrane [GO:0030176]; integral component of membrane [GO:0016021]; junctional sarcoplasmic reticulum membrane [GO:0014701]; plasma membrane [GO:0005886]; sarcoplasmic reticulum lumen [GO:0033018]; sarcoplasmic reticulum membrane [GO:0033017]; calcium ion binding [GO:0005509]; electron transfer activity [GO:0009055]; peptidyl-aspartic acid 3-dioxygenase activity [GO:0062101]; structural constituent of muscle [GO:0008307]; structural molecule activity [GO:0005198]; transmembrane transporter binding [GO:0044325]; activation of cysteine-type endopeptidase activity [GO:0097202]; activation of store-operated calcium channel activity [GO:0032237]; calcium ion homeostasis [GO:0055074]; calcium ion transmembrane transport [GO:0070588]; cell population proliferation [GO:0008283]; cellular response to calcium ion [GO:0071277]; detection of calcium ion [GO:0005513]; face morphogenesis [GO:0060325]; limb morphogenesis [GO:0035108]; muscle contraction [GO:0006936]; negative regulation of cell population proliferation [GO:0008285]; pattern

								communication by electrical coupling [GO:0010649]; regulation of inositol 1,4,5-trisphosphate-sensitive calcium-release channel activity [GO:0031585]; regulation of protein depolymerization [GO:1901879]; regulation of protein stability [GO:0031647]; regulation of release of sequestered calcium ion into cytosol by sarcoplasmic reticulum [GO:0010880]; regulation of ryanodine-sensitive calcium-release channel activity [GO:0060314]; response to ATP [GO:0033198]; roof of mouth development [GO:0060021]	specification process [GO:0007389]; peptidyl-aspartic acid hydroxylation [GO:0042264]; positive regulation of calcium ion transport into cytosol [GO:0010524]; positive regulation of intracellular protein transport [GO:0090316]; positive regulation of proteolysis [GO:0045862]; positive regulation of ryanodine-sensitive calcium-release channel activity [GO:0060316]; positive regulation of transcription, DNA-templated [GO:0045893]; regulation of cardiac muscle contraction by regulation of the release of sequestered calcium ion [GO:0010881]; regulation of cell communication by electrical coupling [GO:0010649]; regulation of inositol 1,4,5-trisphosphate-sensitive calcium-release channel activity [GO:0031585]; regulation of protein depolymerization [GO:1901879]; regulation of protein stability [GO:0031647]; regulation of release of sequestered calcium ion into cytosol by sarcoplasmic reticulum [GO:0010880]; regulation of ryanodine-sensitive calcium-release channel activity [GO:0060314]; response to ATP [GO:0033198]; roof of mouth development [GO:0060021]
P01782	IGHV3-9	Immunoglobulin heavy variable 3-9 OS	12.9	2.47	1.30	6.05E-02	1.22	B cell receptor signaling pathway [GO:0050853]; complement activation, classical pathway [GO:0006958]; defense response to bacterium [GO:0042742]; immune response [GO:0006955]; innate immune response [GO:0045087]; phagocytosis, engulfment [GO:0006911]; phagocytosis, recognition [GO:0006910]; positive regulation of B cell activation [GO:0050871]	external side of plasma membrane [GO:0009897]; extracellular exosome [GO:0070062]; extracellular region [GO:0005576]; immunoglobulin complex, circulating [GO:0042571]; plasma membrane [GO:0005886]; antigen binding [GO:0003823]; immunoglobulin receptor binding [GO:0034987]; B cell receptor signaling pathway [GO:0050853]; complement activation, classical pathway [GO:0006958]; defense response to bacterium [GO:0042742]; immune response [GO:0006955]; innate immune response [GO:0045087]; phagocytosis, engulfment [GO:0006911]; phagocytosis, recognition [GO:0006910]; positive regulation of B cell activation [GO:0050871]

Q13813	SPTAN1	Spectrin alpha chain, non-erythrocytic 1 OS	284.4	2.41	1.27	8.65E-02	1.06	actin cytoskeleton organization [GO:0030036]; actin filament capping [GO:0051693]	cell junction [GO:0030054]; cell projection [GO:0042995]; cortical actin cytoskeleton [GO:0030864]; cytosol [GO:0005829]; extracellular exosome [GO:0070062]; extracellular region [GO:0005576]; extracellular vesicle [GO:1903561]; intracellular membrane-bounded organelle [GO:0043231]; membrane [GO:0016020]; microtubule cytoskeleton [GO:0015630]; plasma membrane [GO:0005886]; specific granule lumen [GO:0035580]; spectrin [GO:0008091]; tertiary granule lumen [GO:1904724]; actin binding [GO:0003779]; actin filament binding [GO:0051015]; cadherin binding [GO:0045296]; calcium ion binding [GO:0005509]; calmodulin binding [GO:0005516]; structural constituent of cytoskeleton [GO:0005200]; actin cytoskeleton organization [GO:0030036]; actin filament capping [GO:0051693]
P14625	HSP90B1	Endoplasmin OS	92.4	2.40	1.26	6.94E-02	1.16	actin rod assembly [GO:0031247]; cellular response to ATP [GO:0071318]; negative regulation of apoptotic process [GO:0043066]; protein folding [GO:0006457]; protein folding in endoplasmic reticulum [GO:0034975]; protein transport [GO:0015031]; regulation of phosphoprotein phosphatase activity [GO:0043666]; response to endoplasmic reticulum stress [GO:0034976]; response to hypoxia [GO:0001666]; retrograde protein transport, ER to cytosol [GO:0030970]; sequestering of calcium ion [GO:0051208]; ubiquitin-dependent ERAD pathway [GO:0030433]	collagen-containing extracellular matrix [GO:0062023]; cytosol [GO:0005829]; endocytic vesicle lumen [GO:0071682]; endoplasmic reticulum [GO:0005783]; endoplasmic reticulum chaperone complex [GO:0034663]; endoplasmic reticulum lumen [GO:0005788]; endoplasmic reticulum membrane [GO:0005789]; extracellular exosome [GO:0070062]; extracellular region [GO:0005576]; focal adhesion [GO:0005925]; melanosome [GO:0042470]; membrane [GO:0016020]; midbody [GO:0030496]; nucleus [GO:0005634]; perinuclear region of cytoplasm [GO:0048471]; protein-containing complex [GO:0032991]; sarcoplasmic reticulum lumen [GO:0033018]; sperm plasma membrane [GO:0097524]; ATP binding [GO:0005524]; ATP hydrolysis activity [GO:0016887]; calcium ion binding [GO:0005509]; low-density lipoprotein particle receptor binding [GO:0050750]; protein phosphatase binding [GO:0019903]; RNA binding [GO:0003723]; unfolded protein binding [GO:0051082]; actin rod assembly [GO:0031247]; cellular response to ATP [GO:0071318]; negative regulation of apoptotic process [GO:0043066]; protein folding [GO:0006457]; protein folding in

									endoplasmic reticulum [GO:0034975]; protein transport [GO:0015031]; regulation of phosphoprotein phosphatase activity [GO:0043666]; response to endoplasmic reticulum stress [GO:0034976]; response to hypoxia [GO:0001666]; retrograde protein transport, ER to cytosol [GO:0030970]; sequestering of calcium ion [GO:0051208]; ubiquitin-dependent ERAD pathway [GO:0030433]
O00746	NME4	Nucleoside diphosphate kinase, mitochondrial OS	20.6	2.28	1.19	9.00E-02	1.05	CTP biosynthetic process [GO:0006241]; GTP biosynthetic process [GO:0006183]; lipid transport [GO:0006869]; nucleoside diphosphate phosphorylation [GO:0006165]; nucleoside metabolic process [GO:0009116]; UTP biosynthetic process [GO:0006228]	mitochondrial inner membrane [GO:0005743]; mitochondrial intermembrane space [GO:0005758]; mitochondrial matrix [GO:0005759]; mitochondrion [GO:0005739]; ATP binding [GO:0005524]; cardiolipin binding [GO:1901612]; metal ion binding [GO:0046872]; nucleoside diphosphate kinase activity [GO:0004550]; CTP biosynthetic process [GO:0006241]; GTP biosynthetic process [GO:0006183]; lipid transport [GO:0006869]; nucleoside diphosphate phosphorylation [GO:0006165]; nucleoside metabolic process [GO:0009116]; UTP biosynthetic process [GO:0006228]

P07900	HSP90AA1	Heat shock protein HSP 90-alpha OS	84.6	2.27	1.18	9.00E-02	1.05	activation of innate immune response [GO:0002218]; axon extension [GO:0048675]; cardiac muscle cell apoptotic process [GO:0010659]; cellular response to heat [GO:0034605]; cellular response to virus [GO:0098586]; central nervous system neuron axonogenesis [GO:0021955]; chaperone-mediated autophagy [GO:0061684]; chaperone-mediated protein complex assembly [GO:0051131]; establishment of cell polarity [GO:0030010]; mitochondrial transport [GO:0006839]; neuron migration [GO:0001764]; positive regulation of cardiac muscle contraction [GO:0060452]; positive regulation of cell size [GO:0045793]; positive regulation of defense response to virus by host [GO:0002230]; positive regulation of interferon-beta production [GO:0032728]; positive regulation of lamellipodium assembly [GO:0010592]; positive regulation of nitric oxide biosynthetic process [GO:0045429]; positive regulation of peptidyl-serine phosphorylation [GO:0033138]; positive regulation of protein catabolic process [GO:0045732]; positive regulation of protein import into nucleus [GO:0042307]; positive regulation of protein kinase B signaling [GO:0051897]; positive regulation of protein phosphorylation [GO:0001934]; positive regulation of protein polymerization [GO:0032273]; positive regulation of tau-protein kinase activity [GO:1902949]; positive regulation of telomerase activity [GO:0051973]; protein folding [GO:0006457]; protein insertion into mitochondrial outer membrane [GO:0045040]; protein refolding [GO:0042026]; protein stabilization [GO:0050821]; protein unfolding [GO:0043335]; regulation of apoptotic process [GO:0042981]; regulation of protein	apical plasma membrane [GO:0016324]; axonal growth cone [GO:0044295]; basolateral plasma membrane [GO:0016323]; brush border membrane [GO:0031526]; cell surface [GO:0009986]; collagen-containing extracellular matrix [GO:0062023]; cytoplasm [GO:0005737]; cytosol [GO:0005829]; dendritic growth cone [GO:0044294]; endocytic vesicle lumen [GO:0071682]; extracellular exosome [GO:0070062]; extracellular region [GO:0005576]; ficolin-1-rich granule lumen [GO:1904813]; lysosomal lumen [GO:0043202]; melanosome [GO:0042470]; membrane [GO:0016020]; mitochondrion [GO:0005739]; myelin sheath [GO:0043209]; neuronal cell body [GO:0043025]; nucleoplasm [GO:0005654]; nucleus [GO:0005634]; perinuclear region of cytoplasm [GO:0048471]; plasma membrane [GO:0005886]; protein-containing complex [GO:0032991]; secretory granule lumen [GO:0034774]; sperm mitochondrial sheath [GO:0097226]; sperm plasma membrane [GO:0097524]; ATP binding [GO:0005524]; ATP hydrolysis activity [GO:0016887]; CTP binding [GO:0002135]; dATP binding [GO:0032564]; disordered domain specific binding [GO:0097718]; DNA polymerase binding [GO:0070182]; GTP binding [GO:0005525]; GTPase binding [GO:0051020]; histone deacetylase binding [GO:0042826]; identical protein binding [GO:0042802]; MHC class II protein complex binding [GO:0023026]; mRNA binding [GO:0003729]; nitric-oxide synthase regulator activity [GO:0030235]; protein folding chaperone [GO:0044183]; protein homodimerization activity [GO:0042803]; protein phosphatase binding [GO:0019903]; protein tyrosine kinase binding [GO:1990782]; Rho GDP-dissociation inhibitor binding [GO:0051022]; RNA binding [GO:0003723]; scaffold protein binding [GO:0097110]; sulfonylurea receptor binding [GO:0017098]; tau protein binding [GO:0048156]; TPR domain binding [GO:0030911]; transmembrane transporter binding [GO:0044325]; ubiquitin protein ligase binding [GO:0031625];

								localization [GO:0032880]; regulation of protein ubiquitination [GO:0031396]; regulation of protein-containing complex assembly [GO:0043254]; response to antibiotic [GO:0046677]; response to cocaine [GO:0042220]; response to cold [GO:0009409]; response to estrogen [GO:0043627]; response to heat [GO:0009408]; response to salt stress [GO:0009651]; response to unfolded protein [GO:0006986]; response to xenobiotic stimulus [GO:0009410]; skeletal muscle contraction [GO:0003009]; telomerase holoenzyme complex assembly [GO:1905323]; telomere maintenance via telomerase [GO:0007004]	unfolded protein binding [GO:0051082]; UTP binding [GO:0002134]; activation of innate immune response [GO:0002218]; axon extension [GO:0048675]; cardiac muscle cell apoptotic process [GO:0010659]; cellular response to heat [GO:0034605]; cellular response to virus [GO:0098586]; central nervous system neuron axonogenesis [GO:0021955]; chaperone-mediated autophagy [GO:0061684]; chaperone-mediated protein complex assembly [GO:0051131]; establishment of cell polarity [GO:0030010]; mitochondrial transport [GO:0006839]; neuron migration [GO:0001764]; positive regulation of cardiac muscle contraction [GO:0060452]; positive regulation of cell size [GO:0045793]; positive regulation of defense response to virus by host [GO:0002230]; positive regulation of interferon-beta production [GO:0032728]; positive regulation of lamellipodium assembly [GO:0010592]; positive regulation of nitric oxide biosynthetic process [GO:0045429]; positive regulation of peptidyl-serine phosphorylation [GO:0033138]; positive regulation of protein catabolic process [GO:0045732]; positive regulation of protein import into nucleus [GO:0042307]; positive regulation of protein kinase B signaling [GO:0051897]; positive regulation of protein phosphorylation [GO:0001934]; positive regulation of protein polymerization [GO:0032273]; positive regulation of tau-protein kinase activity [GO:1902949]; positive regulation of telomerase activity [GO:0051973]; protein folding [GO:0006457]; protein insertion into mitochondrial outer membrane [GO:0045040]; protein refolding [GO:0042026]; protein stabilization [GO:0050821]; protein unfolding [GO:0043335]; regulation of apoptotic process [GO:0042981]; regulation of protein localization [GO:0032880]; regulation of protein ubiquitination [GO:0031396]; regulation of protein-containing complex assembly [GO:0043254]; response to antibiotic [GO:0046677]; response to cocaine [GO:0042220]; response to cold [GO:0009409]; response to estrogen [GO:0043627];

									response to heat [GO:0009408]; response to salt stress [GO:0009651]; response to unfolded protein [GO:0006986]; response to xenobiotic stimulus [GO:0009410]; skeletal muscle contraction [GO:0003009]; telomerase holoenzyme complex assembly [GO:1905323]; telomere maintenance via telomerase [GO:0007004]
P42892	ECE1	Endothelin-converting enzyme 1 OS	87.1	2.24	1.17	9.61E-02	1.02	axonogenesis involved in innervation [GO:0060385]; bradykinin catabolic process [GO:0010815]; calcitonin catabolic process [GO:0010816]; ear development [GO:0043583]; embryonic digit morphogenesis [GO:0042733]; embryonic heart tube development [GO:0035050]; endothelin maturation [GO:0034959]; G protein-coupled receptor signaling pathway [GO:0007186]; heart development [GO:0007507]; hormone catabolic process [GO:0042447]; peptide hormone processing [GO:0016486]; pharyngeal system development [GO:0060037]; positive regulation of receptor recycling [GO:0001921]; protein processing	early endosome [GO:0005769]; endosome [GO:0005768]; external side of plasma membrane [GO:0009897]; extracellular exosome [GO:0070062]; integral component of membrane [GO:0016021]; intrinsic component of endosome membrane [GO:0031302]; lysosomal membrane [GO:0005765]; membrane [GO:0016020]; perinuclear region of cytoplasm [GO:0048471]; plasma membrane [GO:0005886]; vesicle [GO:0031982]; Weibel-Palade body [GO:0033093]; endopeptidase activity [GO:0004175]; metalloendopeptidase activity [GO:0004222]; peptide hormone binding [GO:0017046]; protein homodimerization activity [GO:0042803]; zinc ion binding [GO:0008270]; axonogenesis involved in innervation [GO:0060385]; bradykinin catabolic process [GO:0010815];

								[GO:0016485]; regulation of systemic arterial blood pressure by endothelin [GO:0003100]; regulation of vasoconstriction [GO:0019229]; semaphorin-plexin signaling pathway involved in axon guidance [GO:1902287]; substance P catabolic process [GO:0010814]; sympathetic neuron axon guidance [GO:0097492]	calcitonin catabolic process [GO:0010816]; ear development [GO:0043583]; embryonic digit morphogenesis [GO:0042733]; embryonic heart tube development [GO:0035050]; endothelin maturation [GO:0034959]; G protein-coupled receptor signaling pathway [GO:0007186]; heart development [GO:0007507]; hormone catabolic process [GO:0042447]; peptide hormone processing [GO:0016486]; pharyngeal system development [GO:0060037]; positive regulation of receptor recycling [GO:0001921]; protein processing [GO:0016485]; regulation of systemic arterial blood pressure by endothelin [GO:0003100]; regulation of vasoconstriction [GO:0019229]; semaphorin-plexin signaling pathway involved in axon guidance [GO:1902287]; substance P catabolic process [GO:0010814]; sympathetic neuron axon guidance [GO:0097492]
P01009	SERPINA1	Alpha-1-antitrypsin OS	46.7	2.24	1.16	9.74E-02	1.01	acute-phase response [GO:0006953]; blood coagulation [GO:0007596]; negative regulation of endopeptidase activity [GO:0010951]	collagen-containing extracellular matrix [GO:0062023]; COPII-coated ER to Golgi transport vesicle [GO:0030134]; endoplasmic reticulum [GO:0005783]; endoplasmic reticulum lumen [GO:0005788]; endoplasmic reticulum-Golgi intermediate compartment membrane [GO:0033116]; extracellular exosome [GO:0070062]; extracellular region [GO:0005576]; extracellular space [GO:0005615]; ficolin-1-rich granule lumen [GO:1904813]; Golgi apparatus [GO:0005794]; intracellular membrane-bounded organelle [GO:0043231]; platelet alpha granule lumen [GO:0031093]; identical protein binding [GO:0042802]; protease binding [GO:0002020]; serine-type endopeptidase inhibitor activity [GO:0004867]; acute-phase response [GO:0006953]; blood coagulation [GO:0007596]; negative regulation of endopeptidase activity [GO:0010951]
Q9Y3U8	RPL36	60S ribosomal protein L36 OS	12.2	0.15	-2.72	9.00E-02	1.05	cytoplasmic translation [GO:0002181]; translation [GO:0006412]	cytoplasm [GO:0005737]; cytosol [GO:0005829]; cytosolic large ribosomal subunit [GO:0022625]; cytosolic ribosome [GO:0022626]; endoplasmic reticulum [GO:0005783]; membrane [GO:0016020];

									nucleolus [GO:0005730]; polysomal ribosome [GO:0042788]; RNA binding [GO:0003723]; structural constituent of ribosome [GO:0003735]; cytoplasmic translation [GO:0002181]; translation [GO:0006412]
P01011	SERPINA3	Alpha-1-antichymotrypsin OS	47.6	0.15	-2.75	8.20E-02	1.09	acute-phase response [GO:0006953]; inflammatory response [GO:0006954]; maintenance of gastrointestinal epithelium [GO:0030277]; negative regulation of endopeptidase activity [GO:0010951]; regulation of lipid metabolic process [GO:0019216]	azurophil granule lumen [GO:0035578]; blood microparticle [GO:0072562]; collagen-containing extracellular matrix [GO:0062023]; extracellular exosome [GO:0070062]; extracellular region [GO:0005576]; extracellular space [GO:0005615]; nucleus [GO:0005634]; platelet alpha granule lumen [GO:0031093]; secretory granule lumen [GO:0034774]; DNA binding [GO:0003677]; serine-type endopeptidase inhibitor activity [GO:0004867]; acute-phase response [GO:0006953]; inflammatory response [GO:0006954]; maintenance of gastrointestinal epithelium [GO:0030277]; negative regulation of endopeptidase activity [GO:0010951]; regulation of lipid metabolic process [GO:0019216]
P26583	HMGB2	High mobility group protein B2 OS	24	0.14	-2.83	6.24E-02	1.21	cell chemotaxis [GO:0060326]; cellular response to lipopolysaccharide [GO:0071222]; chromatin organization [GO:0006325]; defense response to Gram-negative bacterium [GO:0050829]; defense response to Gram-positive bacterium [GO:0050830]; DNA geometric change [GO:0032392]; DNA topological change [GO:0006265]; double-strand break repair via nonhomologous end joining [GO:0006303]; extrinsic apoptotic signaling pathway via death domain receptors [GO:0008625]; inflammatory response to antigenic stimulus [GO:0002437]; innate immune response [GO:0045087]; male gonad development [GO:0008584]; negative regulation of extrinsic apoptotic signaling pathway via death domain receptors [GO:1902042]; negative regulation of gene expression [GO:0010629]; negative regulation of transcription by RNA polymerase II [GO:0000122]; negative regulation of	chromatin [GO:0000785]; condensed chromosome [GO:0000793]; cytoplasm [GO:0005737]; extracellular space [GO:0005615]; nucleolus [GO:0005730]; nucleoplasm [GO:0005654]; nucleus [GO:0005634]; perinuclear region of cytoplasm [GO:0048471]; protein-containing complex [GO:0032991]; chemoattractant activity [GO:0042056]; cis-regulatory region sequence-specific DNA binding [GO:0000987]; damaged DNA binding [GO:0003684]; DNA binding [GO:0003677]; DNA binding, bending [GO:0008301]; DNA-binding transcription factor binding [GO:0140297]; double-stranded DNA binding [GO:0003690]; four-way junction DNA binding [GO:0000400]; non-sequence-specific DNA binding, bending [GO:0044378]; protein domain specific binding [GO:0019904]; RAGE receptor binding [GO:0050786]; RNA binding [GO:0003723]; single-stranded DNA binding [GO:0003697]; supercoiled DNA binding [GO:0097100]; transcription cis-regulatory region binding [GO:0000976]; transcription coactivator activity [GO:0003713]; transcription factor binding

								transcription, DNA-templated [GO:0045892]; nucleosome assembly [GO:0006334]; positive regulation of DNA binding [GO:0043388]; positive regulation of endothelial cell proliferation [GO:0001938]; positive regulation of erythrocyte differentiation [GO:0045648]; positive regulation of innate immune response [GO:0045089]; positive regulation of interferon-beta production [GO:0032728]; positive regulation of megakaryocyte differentiation [GO:0045654]; positive regulation of nuclease activity [GO:0032075]; positive regulation of transcription by RNA polymerase II [GO:0045944]; positive regulation of transcription, DNA-templated [GO:0045893]; regulation of neurogenesis [GO:0050767]; regulation of stem cell proliferation [GO:0072091]; regulation of transcription by RNA polymerase II [GO:0006357]; response to lipopolysaccharide [GO:0032496]; response to steroid hormone [GO:0048545]; spermatid nucleus differentiation [GO:0007289]; V(D)J recombination [GO:0033151]	[GO:0008134]; cell chemotaxis [GO:0060326]; cellular response to lipopolysaccharide [GO:0071222]; chromatin organization [GO:0006325]; defense response to Gram-negative bacterium [GO:0050829]; defense response to Gram-positive bacterium [GO:0050830]; DNA geometric change [GO:0032392]; DNA topological change [GO:0006265]; double-strand break repair via nonhomologous end joining [GO:0006303]; extrinsic apoptotic signaling pathway via death domain receptors [GO:0008625]; inflammatory response to antigenic stimulus [GO:0002437]; innate immune response [GO:0045087]; male gonad development [GO:0008584]; negative regulation of extrinsic apoptotic signaling pathway via death domain receptors [GO:1902042]; negative regulation of gene expression [GO:0010629]; negative regulation of transcription by RNA polymerase II [GO:0000122]; negative regulation of transcription, DNA-templated [GO:0045892]; nucleosome assembly [GO:0006334]; positive regulation of DNA binding [GO:0043388]; positive regulation of endothelial cell proliferation [GO:0001938]; positive regulation of erythrocyte differentiation [GO:0045648]; positive regulation of innate immune response [GO:0045089]; positive regulation of interferon-beta production [GO:0032728]; positive regulation of megakaryocyte differentiation [GO:0045654]; positive regulation of nuclease activity [GO:0032075]; positive regulation of transcription by RNA polymerase II [GO:0045944]; positive regulation of transcription, DNA-templated [GO:0045893]; regulation of neurogenesis [GO:0050767]; regulation of stem cell proliferation [GO:0072091]; regulation of transcription by RNA polymerase II [GO:0006357]; response to lipopolysaccharide [GO:0032496]; response to steroid hormone [GO:0048545]; spermatid nucleus differentiation [GO:0007289]; V(D)J recombination [GO:0033151]

O00194	RAB27B	Ras-related protein Rab-27B OS	24.6	0.13	-2.90	4.97E-02	1.30	anterograde axonal protein transport [GO:0099641]; exocytosis [GO:0006887]; multivesicular body sorting pathway [GO:0071985]; positive regulation of exocytosis [GO:0045921]; synaptic vesicle endocytosis [GO:0048488]; vesicle-mediated transport [GO:0016192]	anchored component of synaptic vesicle membrane [GO:0098993]; apical plasma membrane [GO:0016324]; axon cytoplasm [GO:1904115]; exocytic vesicle [GO:0070382]; extracellular exosome [GO:0070062]; Golgi apparatus [GO:0005794]; Golgi stack [GO:0005795]; late endosome [GO:0005770]; melanosome [GO:0042470]; multivesicular body membrane [GO:0032585]; plasma membrane [GO:0005886]; platelet dense granule membrane [GO:0031088]; secretory granule [GO:0030141]; trans-Golgi network transport vesicle [GO:0030140]; zymogen granule membrane [GO:0042589]; G protein activity [GO:0003925]; GDP binding [GO:0019003]; GTP binding [GO:0005525]; GTPase activity [GO:0003924]; myosin V binding [GO:0031489]; protein domain specific binding [GO:0019904]; anterograde axonal protein transport [GO:0099641]; exocytosis [GO:0006887]; multivesicular body sorting pathway [GO:0071985]; positive regulation of exocytosis [GO:0045921]; synaptic vesicle endocytosis [GO:0048488]; vesicle-mediated transport [GO:0016192]
P46778	RPL21	60S ribosomal protein L21 OS	18.6	0.13	-2.92	4.59E-02	1.34	cytoplasmic translation [GO:0002181]; translation [GO:0006412]	cytoplasm [GO:0005737]; cytosol [GO:0005829]; cytosolic large ribosomal subunit [GO:0022625]; cytosolic ribosome [GO:0022626]; endoplasmic reticulum [GO:0005783]; membrane [GO:0016020]; RNA binding [GO:0003723]; structural constituent of ribosome [GO:0003735]; cytoplasmic translation [GO:0002181]; translation [GO:0006412]
O95197	RTN3	Reticulon-3 OS	112.5	0.13	-2.94	4.25E-02	1.37	apoptotic process [GO:0006915]; brain development [GO:0007420]; endoplasmic reticulum tubular network formation [GO:0071787]; endoplasmic reticulum tubular network organization [GO:0071786]; negative regulation of amyloid-beta formation [GO:1902430]; neuron differentiation [GO:0030182]; vesicle-mediated transport [GO:0016192]	endoplasmic reticulum [GO:0005783]; endoplasmic reticulum membrane [GO:0005789]; extracellular space [GO:0005615]; Golgi apparatus [GO:0005794]; Golgi membrane [GO:0000139]; integral component of membrane [GO:0016021]; neuron projection [GO:0043005]; plasma membrane [GO:0005886]; postsynaptic density [GO:0014069]; apoptotic process [GO:0006915]; brain development [GO:0007420]; endoplasmic reticulum tubular network formation [GO:0071787]; endoplasmic reticulum tubular network organization

									[GO:0071786]; negative regulation of amyloid-beta formation [GO:1902430]; neuron differentiation [GO:0030182]; vesicle-mediated transport [GO:0016192]
Q71U36	TUBA1A	Tubulin alpha-1A chain OS	50.1	0.12	-3.05	2.88E-02	1.54	cell division [GO:0051301]; cytoskeleton-dependent intracellular transport [GO:0030705]; microtubule cytoskeleton organization [GO:0000226]; microtubule-based process [GO:0007017]; mitotic cell cycle [GO:0000278]; regulation of synapse organization [GO:0050807]	cytoplasm [GO:0005737]; cytoplasmic microtubule [GO:0005881]; cytoplasmic ribonucleoprotein granule [GO:0036464]; cytosol [GO:0005829]; extracellular exosome [GO:0070062]; microtubule [GO:0005874]; microtubule cytoskeleton [GO:0015630]; neuromuscular junction [GO:0031594]; nucleus [GO:0005634]; recycling endosome [GO:0055037]; GTP binding [GO:0005525]; identical protein binding [GO:0042802]; structural constituent of cytoskeleton [GO:0005200]; structural molecule activity [GO:0005198]; cell division [GO:0051301]; cytoskeleton-dependent intracellular transport [GO:0030705]; microtubule cytoskeleton organization [GO:0000226]; microtubule-based process [GO:0007017]; mitotic cell cycle [GO:0000278]; regulation of synapse organization [GO:0050807]
P62847	RPS24	40S ribosomal protein S24 OS	15.4	0.12	-3.10	2.43E-02	1.61	cytoplasmic translation [GO:0002181]; erythrocyte homeostasis [GO:0034101]; ribosomal small subunit biogenesis [GO:0042274]; rRNA processing [GO:0006364]; translation [GO:0006412]	cytoplasm [GO:0005737]; cytosol [GO:0005829]; cytosolic ribosome [GO:0022626]; cytosolic small ribosomal subunit [GO:0022627]; endoplasmic reticulum [GO:0005783]; membrane [GO:0016020]; nucleoplasm [GO:0005654]; nucleus [GO:0005634]; small ribosomal subunit [GO:0015935]; RNA binding [GO:0003723]; structural constituent of ribosome [GO:0003735]; translation initiation factor binding [GO:0031369]; cytoplasmic translation [GO:0002181]; erythrocyte homeostasis [GO:0034101]; ribosomal small subunit biogenesis [GO:0042274]; rRNA processing [GO:0006364]; translation [GO:0006412]
P62910	RPL32	60S ribosomal protein L32 OS	15.9	0.10	-3.35	8.21E-03	2.09	cytoplasmic translation [GO:0002181]; translation [GO:0006412]	cytoplasm [GO:0005737]; cytosol [GO:0005829]; cytosolic large ribosomal subunit [GO:0022625]; cytosolic ribosome [GO:0022626]; membrane [GO:0016020]; polysomal ribosome [GO:0042788];

									RNA binding [GO:0003723]; structural constituent of ribosome [GO:0003735]; cytoplasmic translation [GO:0002181]; translation [GO:0006412]
Q9NX47	MARCHF5	E3 ubiquitin-protein ligase MARCHF5 OS	31.2	0.10	-3.35	3.95E-02	1.40	positive regulation of mitochondrial fission [GO:0090141]; protein autoubiquitination [GO:0051865]; protein localization to mitochondrion [GO:0070585]; protein polyubiquitination [GO:0000209]; regulation of mitochondrial fission [GO:0090140]	endoplasmic reticulum [GO:0005783]; endoplasmic reticulum membrane [GO:0005789]; integral component of membrane [GO:0016021]; membrane [GO:0016020]; mitochondrial outer membrane [GO:0005741]; mitochondrion [GO:0005739]; GTPase binding [GO:0051020]; ubiquitin protein ligase activity [GO:0061630]; zinc ion binding [GO:0008270]; positive regulation of mitochondrial fission [GO:0090141]; protein autoubiquitination [GO:0051865]; protein localization to mitochondrion [GO:0070585]; protein polyubiquitination [GO:0000209]; regulation of mitochondrial fission [GO:0090140]
P08571	CD14	Monocyte differentiation antigen CD14 OS	40.1	0.10	-3.38	1.43E-02	1.85	apoptotic process [GO:0006915]; cell surface receptor signaling pathway [GO:0007166]; cellular response to diacyl bacterial lipopeptide [GO:0071726]; cellular response to lipopolysaccharide [GO:0071222]; cellular response to lipoteichoic acid [GO:0071223]; cellular response to molecule of bacterial origin [GO:0071219]; cellular response to triacyl bacterial lipopeptide [GO:0071727]; inflammatory response [GO:0006954]; innate immune response [GO:0045087]; lipopolysaccharide-mediated signaling pathway [GO:0031663]; phagocytosis [GO:0006909]; positive regulation of endocytosis [GO:0045807]; positive regulation of interferon-gamma production [GO:0032729]; positive regulation of interleukin-8 production [GO:0032757]; positive regulation of lipopolysaccharide-mediated signaling pathway [GO:0031666]; positive regulation of NIK/NF-kappaB signaling [GO:1901224]; positive regulation of toll-like receptor 4 signaling pathway [GO:0034145]; positive regulation of tumor	anchored component of external side of plasma membrane [GO:0031362]; endosome membrane [GO:0010008]; external side of plasma membrane [GO:0009897]; extracellular exosome [GO:0070062]; extracellular region [GO:0005576]; extracellular space [GO:0005615]; Golgi apparatus [GO:0005794]; lipopolysaccharide receptor complex [GO:0046696]; membrane raft [GO:0045121]; plasma membrane [GO:0005886]; secretory granule membrane [GO:0030667]; lipopeptide binding [GO:0071723]; lipopolysaccharide binding [GO:0001530]; lipoteichoic acid binding [GO:0070891]; opsonin receptor activity [GO:0001847]; peptidoglycan immune receptor activity [GO:0016019]; apoptotic process [GO:0006915]; cell surface receptor signaling pathway [GO:0007166]; cellular response to diacyl bacterial lipopeptide [GO:0071726]; cellular response to lipopolysaccharide [GO:0071222]; cellular response to lipoteichoic acid [GO:0071223]; cellular response to molecule of bacterial origin [GO:0071219]; cellular response to triacyl bacterial lipopeptide [GO:0071727]; inflammatory response [GO:0006954]; innate immune response [GO:0045087]; lipopolysaccharide-mediated

								necrosis factor production [GO:0032760]; positive regulation of type I interferon production [GO:0032481]; receptor-mediated endocytosis [GO:0006898]; response to electrical stimulus [GO:0051602]; response to ethanol [GO:0045471]; response to heat [GO:0009408]; response to magnesium ion [GO:0032026]; response to tumor necrosis factor [GO:0034612]; toll-like receptor 4 signaling pathway [GO:0034142]	signaling pathway [GO:0031663]; phagocytosis [GO:0006909]; positive regulation of endocytosis [GO:0045807]; positive regulation of interferon-gamma production [GO:0032729]; positive regulation of interleukin-8 production [GO:0032757]; positive regulation of lipopolysaccharide-mediated signaling pathway [GO:0031666]; positive regulation of NIK/NF-kappaB signaling [GO:1901224]; positive regulation of toll-like receptor 4 signaling pathway [GO:0034145]; positive regulation of tumor necrosis factor production [GO:0032760]; positive regulation of type I interferon production [GO:0032481]; receptor-mediated endocytosis [GO:0006898]; response to electrical stimulus [GO:0051602]; response to ethanol [GO:0045471]; response to heat [GO:0009408]; response to magnesium ion [GO:0032026]; response to tumor necrosis factor [GO:0034612]; toll-like receptor 4 signaling pathway [GO:0034142]
P42766	RPL35	60S ribosomal protein L35 OS	14.5	0.09	-3.47	4.89E-03	2.31	cytoplasmic translation [GO:0002181]; maturation of LSU-rRNA from tricistronic rRNA transcript (SSU-rRNA, 5.8S rRNA, LSU-rRNA) [GO:0000463]; translation [GO:0006412]	cytoplasm [GO:0005737]; cytosol [GO:0005829]; cytosolic large ribosomal subunit [GO:0022625]; cytosolic ribosome [GO:0022626]; membrane [GO:0016020]; nucleolus [GO:0005730]; mRNA binding [GO:0003729]; RNA binding [GO:0003723]; structural constituent of ribosome [GO:0003735]; cytoplasmic translation [GO:0002181]; maturation of LSU-rRNA from tricistronic rRNA transcript (SSU-rRNA, 5.8S rRNA, LSU-rRNA) [GO:0000463]; translation [GO:0006412]
P11169	SLC2A3	Solute carrier family 2, facilitated glucose transporter member 3 OS	53.9	0.09	-3.52	5.71E-03	2.24	carbohydrate metabolic process [GO:0005975]; dehydroascorbic acid transport [GO:0070837]; galactose transmembrane transport [GO:0015757]; glucose import [GO:0046323]; glucose import across plasma membrane [GO:0098708]; glucose transmembrane transport [GO:1904659]; L-ascorbic acid metabolic process [GO:0019852]; monosaccharide transmembrane transport [GO:0015749];	cell projection [GO:0042995]; extracellular exosome [GO:0070062]; ficolin-1-rich granule membrane [GO:0101003]; integral component of membrane [GO:0016021]; integral component of plasma membrane [GO:0005887]; membrane [GO:0016020]; perikaryon [GO:0043204]; plasma membrane [GO:0005886]; secretory granule membrane [GO:0030667]; specific granule membrane [GO:0035579]; tertiary granule membrane [GO:0070821]; D-glucose transmembrane transporter activity [GO:0055056]; dehydroascorbic

								transport across blood-brain barrier [GO:0150104]	acid transmembrane transporter activity [GO:0033300]; galactose transmembrane transporter activity [GO:0005354]; glucose binding [GO:0005536]; glucose transmembrane transporter activity [GO:0005355]; hexose transmembrane transporter activity [GO:0015149]; carbohydrate metabolic process [GO:0005975]; dehydroascorbic acid transport [GO:0070837]; galactose transmembrane transport [GO:0015757]; glucose import [GO:0046323]; glucose import across plasma membrane [GO:0098708]; glucose transmembrane transport [GO:1904659]; L-ascorbic acid metabolic process [GO:0019852]; monosaccharide transmembrane transport [GO:0015749]; transport across blood-brain barrier [GO:0150104]
Q9NPD3	EXOSC4	Exosome complex component RRP41 OS	26.4	0.07	-3.78	7.77E-03	2.11	defense response to virus [GO:0051607]; DNA deamination [GO:0045006]; histone mRNA catabolic process [GO:0071044]; maturation of 5.8S rRNA [GO:0000460]; nuclear mRNA surveillance [GO:0071028]; nuclear-transcribed mRNA catabolic process [GO:0000956]; nuclear-transcribed mRNA catabolic process, exonucleolytic, 3'-5' [GO:0034427]; polyadenylation-dependent snoRNA 3'-end processing [GO:0071051]; positive regulation of cell growth [GO:0030307]; RNA catabolic process [GO:0006401]; RNA processing [GO:0006396]; rRNA catabolic process [GO:0016075]; rRNA processing [GO:0006364]; U4 snRNA 3'-end processing [GO:0034475]	cytoplasm [GO:0005737]; cytoplasmic exosome (RNase complex) [GO:0000177]; cytosol [GO:0005829]; euchromatin [GO:0000791]; exosome (RNase complex) [GO:0000178]; intracellular membrane-bounded organelle [GO:0043231]; nuclear exosome (RNase complex) [GO:0000176]; nucleolar exosome (RNase complex) [GO:0101019]; nucleolus [GO:0005730]; nucleoplasm [GO:0005654]; nucleus [GO:0005634]; 3'-5'-exoribonuclease activity [GO:0000175]; mRNA 3'-UTR AU-rich region binding [GO:0035925]; defense response to virus [GO:0051607]; DNA deamination [GO:0045006]; histone mRNA catabolic process [GO:0071044]; maturation of 5.8S rRNA [GO:0000460]; nuclear mRNA surveillance [GO:0071028]; nuclear-transcribed mRNA catabolic process [GO:0000956]; nuclear-transcribed mRNA catabolic process, exonucleolytic, 3'-5' [GO:0034427]; polyadenylation-dependent snoRNA 3'-end processing [GO:0071051]; positive regulation of cell growth [GO:0030307]; RNA catabolic process [GO:0006401]; RNA processing [GO:0006396]; rRNA catabolic process [GO:0016075]; rRNA processing [GO:0006364]; U4 snRNA 3'-end processing [GO:0034475]

P04083	ANXA1	Annexin A1 OS	38.7	0.05	-4.47	1.96E-05	4.71	actin cytoskeleton reorganization [GO:0031532]; adaptive immune response [GO:0002250]; alpha-beta T cell differentiation [GO:0046632]; arachidonic acid secretion [GO:0050482]; cell surface receptor signaling pathway [GO:0007166]; cellular response to glucocorticoid stimulus [GO:0071385]; cellular response to hydrogen peroxide [GO:0070301]; cellular response to vascular endothelial growth factor stimulus [GO:0035924]; DNA duplex unwinding [GO:0032508]; endocrine pancreas development [GO:0031018]; G protein-coupled receptor signaling pathway, coupled to cyclic nucleotide second messenger [GO:0007187]; gliogenesis [GO:0042063]; granulocyte chemotaxis [GO:0071621]; hepatocyte differentiation [GO:0070365]; inflammatory response [GO:0006954]; innate immune response [GO:0045087]; insulin secretion [GO:0030073]; keratinocyte differentiation [GO:0030216]; monocyte chemotaxis [GO:0002548]; myoblast migration involved in skeletal muscle regeneration [GO:0014839]; negative regulation of apoptotic process [GO:0043066]; negative regulation of exocytosis [GO:0045920]; negative regulation of interleukin-8 production [GO:0032717]; negative regulation of phospholipase A2 activity [GO:1900138]; negative regulation of T-helper 2 cell differentiation [GO:0045629]; neutrophil activation [GO:0042119]; neutrophil clearance [GO:0097350]; neutrophil homeostasis [GO:0001780]; peptide cross-linking [GO:0018149]; phagocytosis [GO:0006909]; positive regulation of cell migration involved in sprouting angiogenesis [GO:0090050]; positive regulation of G1/S transition of mitotic cell cycle [GO:1900087]; positive	adherens junction [GO:0005912]; apical plasma membrane [GO:0016324]; basolateral plasma membrane [GO:0016323]; cell surface [GO:0009986]; collagen-containing extracellular matrix [GO:0062023]; cornified envelope [GO:0001533]; cytoplasm [GO:0005737]; cytosol [GO:0005829]; early endosome membrane [GO:0031901]; endosome [GO:0005768]; extracellular exosome [GO:0070062]; extracellular region [GO:0005576]; extracellular space [GO:0005615]; extrinsic component of endosome membrane [GO:0031313]; extrinsic component of external side of plasma membrane [GO:0031232]; extrinsic component of membrane [GO:0019898]; focal adhesion [GO:0005925]; lateral plasma membrane [GO:0016328]; motile cilium [GO:0031514]; nucleoplasm [GO:0005654]; nucleus [GO:0005634]; phagocytic cup [GO:0001891]; plasma membrane [GO:0005886]; sarcolemma [GO:0042383]; vesicle [GO:0031982]; cadherin binding involved in cell-cell adhesion [GO:0098641]; calcium ion binding [GO:0005509]; calcium-dependent phospholipid binding [GO:0005544]; calcium-dependent protein binding [GO:0048306]; phospholipase A2 inhibitor activity [GO:0019834]; phospholipid binding [GO:0005543]; signaling receptor binding [GO:0005102]; actin cytoskeleton reorganization [GO:0031532]; adaptive immune response [GO:0002250]; alpha-beta T cell differentiation [GO:0046632]; arachidonic acid secretion [GO:0050482]; cell surface receptor signaling pathway [GO:0007166]; cellular response to glucocorticoid stimulus [GO:0071385]; cellular response to hydrogen peroxide [GO:0070301]; cellular response to vascular endothelial growth factor stimulus [GO:0035924]; DNA duplex unwinding [GO:0032508]; endocrine pancreas development [GO:0031018]; G protein-coupled receptor signaling pathway, coupled to cyclic nucleotide second messenger [GO:0007187]; gliogenesis [GO:0042063]; granulocyte chemotaxis

								regulation of interleukin-2 production [GO:0032743]; positive regulation of neutrophil apoptotic process [GO:0033031]; positive regulation of prostaglandin biosynthetic process [GO:0031394]; positive regulation of T cell proliferation [GO:0042102]; positive regulation of T-helper 1 cell differentiation [GO:0045627]; positive regulation of vesicle fusion [GO:0031340]; positive regulation of wound healing [GO:0090303]; prolactin secretion [GO:0070459]; prostate gland development [GO:0030850]; regulation of cell shape [GO:0008360]; regulation of hormone secretion [GO:0046883]; regulation of inflammatory response [GO:0050727]; regulation of interleukin-1 production [GO:0032652]; regulation of leukocyte migration [GO:0002685]; response to estradiol [GO:0032355]; response to interleukin-1 [GO:0070555]; response to peptide hormone [GO:0043434]; response to X-ray [GO:0010165]; signal transduction [GO:0007165]	[GO:0071621]; hepatocyte differentiation [GO:0070365]; inflammatory response [GO:0006954]; innate immune response [GO:0045087]; insulin secretion [GO:0030073]; keratinocyte differentiation [GO:0030216]; monocyte chemotaxis [GO:0002548]; myoblast migration involved in skeletal muscle regeneration [GO:0014839]; negative regulation of apoptotic process [GO:0043066]; negative regulation of exocytosis [GO:0045920]; negative regulation of interleukin-8 production [GO:0032717]; negative regulation of phospholipase A2 activity [GO:1900138]; negative regulation of T-helper 2 cell differentiation [GO:0045629]; neutrophil activation [GO:0042119]; neutrophil clearance [GO:0097350]; neutrophil homeostasis [GO:0001780]; peptide cross-linking [GO:0018149]; phagocytosis [GO:0006909]; positive regulation of cell migration involved in sprouting angiogenesis [GO:0090050]; positive regulation of G1/S transition of mitotic cell cycle [GO:1900087]; positive regulation of interleukin-2 production [GO:0032743]; positive regulation of neutrophil apoptotic process [GO:0033031]; positive regulation of prostaglandin biosynthetic process [GO:0031394]; positive regulation of T cell proliferation [GO:0042102]; positive regulation of T-helper 1 cell differentiation [GO:0045627]; positive regulation of vesicle fusion [GO:0031340]; positive regulation of wound healing [GO:0090303]; prolactin secretion [GO:0070459]; prostate gland development [GO:0030850]; regulation of cell shape [GO:0008360]; regulation of hormone secretion [GO:0046883]; regulation of inflammatory response [GO:0050727]; regulation of interleukin-1 production [GO:0032652]; regulation of leukocyte migration [GO:0002685]; response to estradiol [GO:0032355]; response to interleukin-1 [GO:0070555]; response to peptide hormone [GO:0043434]; response to X-ray [GO:0010165]; signal transduction [GO:0007165]

P68036	UBE2L3	Ubiquitin-conjugating enzyme E2 L3 OS	17.9	0.04	-4.84	2.10E-05	4.68	cell cycle phase transition [GO:0044770]; cell population proliferation [GO:0008283]; cellular response to glucocorticoid stimulus [GO:0071385]; cellular response to steroid hormone stimulus [GO:0071383]; positive regulation of protein targeting to mitochondrion [GO:1903955]; positive regulation of protein ubiquitination [GO:0031398]; positive regulation of ubiquitin-protein transferase activity [GO:0051443]; protein K11-linked ubiquitination [GO:0070979]; protein modification process [GO:0036211]; protein polyubiquitination [GO:0000209]; protein ubiquitination [GO:0016567]; regulation of transcription, DNA-templated [GO:0006355]; ubiquitin-dependent protein catabolic process [GO:0006511]	cytoplasm [GO:0005737]; cytosol [GO:0005829]; nucleoplasm [GO:0005654]; nucleus [GO:0005634]; ubiquitin ligase complex [GO:0000151]; ATP binding [GO:0005524]; enzyme binding [GO:0019899]; RNA binding [GO:0003723]; transcription coactivator activity [GO:0003713]; ubiquitin conjugating enzyme activity [GO:0061631]; ubiquitin protein ligase binding [GO:0031625]; ubiquitin-protein transferase activator activity [GO:0097027]; ubiquitin-protein transferase activity [GO:0004842]; cell cycle phase transition [GO:0044770]; cell population proliferation [GO:0008283]; cellular response to glucocorticoid stimulus [GO:0071385]; cellular response to steroid hormone stimulus [GO:0071383]; positive regulation of protein targeting to mitochondrion [GO:1903955]; positive regulation of protein ubiquitination [GO:0031398]; positive regulation of ubiquitin-protein transferase activity [GO:0051443]; protein K11-linked ubiquitination [GO:0070979]; protein modification process [GO:0036211]; protein polyubiquitination [GO:0000209]; protein ubiquitination [GO:0016567]; regulation of transcription, DNA-templated [GO:0006355]; ubiquitin-dependent protein catabolic process [GO:0006511]
Q13769	THOC5	THO complex subunit 5 homolog OS	78.5	0.01	-6.64	1.91E-16	15.72	monocyte differentiation [GO:0030224]; mRNA export from nucleus [GO:0006406]; mRNA processing [GO:0006397]; negative regulation of DNA damage checkpoint [GO:2000002]; positive regulation of DNA-templated transcription, elongation [GO:0032786]; primitive hemopoiesis [GO:0060215]; RNA splicing [GO:0008380]; viral mRNA export from host cell nucleus [GO:0046784]	cytoplasm [GO:0005737]; nucleoplasm [GO:0005654]; nucleus [GO:0005634]; THO complex [GO:0000347]; THO complex part of transcription export complex [GO:0000445]; transcription export complex [GO:0000346]; mRNA binding [GO:0003729]; monocyte differentiation [GO:0030224]; mRNA export from nucleus [GO:0006406]; mRNA processing [GO:0006397]; negative regulation of DNA damage checkpoint [GO:2000002]; positive regulation of DNA-templated transcription, elongation [GO:0032786]; primitive hemopoiesis [GO:0060215]; RNA splicing [GO:0008380]; viral mRNA export from host cell nucleus [GO:0046784]

Q96QD9	FYTTD1	UAP56-interacting factor OS	35.8	0.01	-6.64	1.91E-16	15.72	mRNA export from nucleus [GO:0006406]	nuclear speck [GO:0016607]; nucleoplasm [GO:0005654]; mRNA binding [GO:0003729]; RNA binding [GO:0003723]; mRNA export from nucleus [GO:0006406]
Q9UBK9	UXT	Protein UXT OS	18.2	0.01	-6.64	1.91E-16	15.72	apoptotic process [GO:0006915]; centrosome cycle [GO:0007098]; microtubule cytoskeleton organization [GO:0000226]; mitochondrion transport along microtubule [GO:0047497]; negative regulation of transcription by RNA polymerase II [GO:0000122]; protein stabilization [GO:0050821]; regulation of transcription by RNA polymerase II [GO:0006357]	centrosome [GO:0005813]; chaperone complex [GO:0101031]; chromatin [GO:0000785]; cytoplasm [GO:0005737]; cytoskeleton [GO:0005856]; nucleoplasm [GO:0005654]; nucleus [GO:0005634]; RPAP3/R2TP/prefoldin-like complex [GO:1990062]; spindle pole [GO:0000922]; beta-tubulin binding [GO:0048487]; chromatin binding [GO:0003682]; microtubule binding [GO:0008017]; transcription coregulator activity [GO:0003712]; transcription corepressor activity [GO:0003714]; apoptotic process [GO:0006915]; centrosome cycle [GO:0007098]; microtubule cytoskeleton organization [GO:0000226]; mitochondrion transport along microtubule [GO:0047497]; negative regulation of transcription by RNA polymerase II [GO:0000122]; protein stabilization [GO:0050821]; regulation of transcription by RNA polymerase II [GO:0006357]
P14635	CCNB1	G2/mitotic-specific cyclin-B1 OS	48.3	0.01	-6.64	1.91E-16	15.72	cell division [GO:0051301]; G2/M transition of mitotic cell cycle [GO:0000086]; in utero embryonic development [GO:0001701]; mitotic cell cycle phase transition [GO:0044772]; mitotic metaphase plate congression [GO:0007080]; mitotic spindle organization [GO:0007052]; positive regulation of attachment of spindle microtubules to kinetochore [GO:0051987]; positive regulation of fibroblast proliferation [GO:0048146]; positive regulation of G2/M transition of mitotic cell cycle [GO:0010971]; positive regulation of mitochondrial ATP synthesis coupled electron transport [GO:1905448]; positive regulation of mitotic cell cycle [GO:0045931]; protein phosphorylation [GO:0006468]; regulation of cyclin-dependent protein serine/threonine kinase activity [GO:0000079]; regulation of	centrosome [GO:0005813]; cyclin B1-CDK1 complex [GO:0097125]; cyclin-dependent protein kinase holoenzyme complex [GO:0000307]; cytoplasm [GO:0005737]; cytosol [GO:0005829]; membrane [GO:0016020]; mitochondrial matrix [GO:0005759]; nucleoplasm [GO:0005654]; nucleus [GO:0005634]; outer kinetochore [GO:0000940]; spindle pole [GO:0000922]; cyclin-dependent protein serine/threonine kinase activator activity [GO:0061575]; cyclin-dependent protein serine/threonine kinase regulator activity [GO:0016538]; patched binding [GO:0005113]; protein kinase binding [GO:0019901]; ubiquitin-like protein ligase binding [GO:0044389]; cell division [GO:0051301]; G2/M transition of mitotic cell cycle [GO:0000086]; in utero embryonic development [GO:0001701]; mitotic cell cycle phase transition [GO:0044772]; mitotic metaphase plate congression [GO:0007080]; mitotic spindle organization

								mitotic cell cycle spindle assembly checkpoint [GO:0090266]	[GO:0007052]; positive regulation of attachment of spindle microtubules to kinetochore [GO:0051987]; positive regulation of fibroblast proliferation [GO:0048146]; positive regulation of G2/M transition of mitotic cell cycle [GO:0010971]; positive regulation of mitochondrial ATP synthesis coupled electron transport [GO:1905448]; positive regulation of mitotic cell cycle [GO:0045931]; protein phosphorylation [GO:0006468]; regulation of cyclin-dependent protein serine/threonine kinase activity [GO:0000079]; regulation of mitotic cell cycle spindle assembly checkpoint [GO:0090266]
Q9Y6A9	SPCS1	Signal peptidase complex subunit 1 OS	18.3	0.01	-6.64	1.91E-16	15.72	protein targeting to ER [GO:0045047]; proteolysis [GO:0006508]; signal peptide processing [GO:0006465]; viral protein processing [GO:0019082]; virion assembly [GO:0019068]	endoplasmic reticulum membrane [GO:0005789]; integral component of endoplasmic reticulum membrane [GO:0030176]; signal peptidase complex [GO:0005787]; protein targeting to ER [GO:0045047]; proteolysis [GO:0006508]; signal peptide processing [GO:0006465]; viral protein processing [GO:0019082]; virion assembly [GO:0019068]
O15270	SPTLC2	Serine palmitoyltransferase 2 OS	62.9	0.01	-6.64	1.91E-16	15.72	adipose tissue development [GO:0060612]; ceramide biosynthetic process [GO:0046513]; positive regulation of lipophagy [GO:1904504]; sphinganine biosynthetic process [GO:0046511]; sphingolipid biosynthetic process [GO:0030148]; sphingomyelin biosynthetic process [GO:0006686]; sphingosine biosynthetic process [GO:0046512]	endoplasmic reticulum membrane [GO:0005789]; integral component of membrane [GO:0016021]; serine C-palmitoyltransferase complex [GO:0017059]; pyridoxal phosphate binding [GO:0030170]; serine C-palmitoyltransferase activity [GO:0004758]; adipose tissue development [GO:0060612]; ceramide biosynthetic process [GO:0046513]; positive regulation of lipophagy [GO:1904504]; sphinganine biosynthetic process [GO:0046511]; sphingolipid biosynthetic process [GO:0030148]; sphingomyelin biosynthetic process [GO:0006686]; sphingosine biosynthetic process [GO:0046512]
Q05682	CALD1	Caldesmon OS	93.2	0.01	-6.64	1.91E-16	15.72	actin filament bundle assembly [GO:0051017]; angiogenesis [GO:0001525]; muscle contraction [GO:0006936]	actin cap [GO:0030478]; actin cytoskeleton [GO:0015629]; cytoskeleton [GO:0005856]; cytosol [GO:0005829]; myofibril [GO:0030016]; plasma membrane [GO:0005886]; actin binding [GO:0003779]; cadherin binding [GO:0045296]; calmodulin binding [GO:0005516]; myosin binding [GO:0017022]; tropomyosin binding [GO:0005523];

									actin filament bundle assembly [GO:0051017]; angiogenesis [GO:0001525]; muscle contraction [GO:0006936]
P14649	MYL6B	Myosin light chain 6B OS	22.8	0.01	-6.64	1.91E-16	15.72	muscle contraction [GO:0006936]; muscle filament sliding [GO:0030049]; skeletal muscle tissue development [GO:0007519]	cytosol [GO:0005829]; extracellular exosome [GO:0070062]; muscle myosin complex [GO:0005859]; myosin complex [GO:0016459]; myosin II complex [GO:0016460]; unconventional myosin complex [GO:0016461]; calcium ion binding [GO:0005509]; cytoskeletal motor activity [GO:0003774]; structural constituent of muscle [GO:0008307]; muscle contraction [GO:0006936]; muscle filament sliding [GO:0030049]; skeletal muscle tissue development [GO:0007519]
Q9NQS7	INCENP	Inner centromere protein OS	105.4	0.01	-6.64	1.91E-16	15.72	chromosome segregation [GO:0007059]; histone phosphorylation [GO:0016572]; meiotic spindle midzone assembly [GO:0051257]; metaphase plate congression [GO:0051310]; mitotic cell cycle [GO:0000278]; mitotic cytokinesis [GO:0000281]; mitotic spindle midzone assembly [GO:0051256]; mitotic spindle organization [GO:0007052]; positive regulation of attachment of mitotic spindle microtubules to kinetochore [GO:1902425]; positive regulation of mitotic cell cycle spindle assembly checkpoint [GO:0090267]; positive regulation of mitotic cytokinesis [GO:1903490]; positive regulation of mitotic sister chromatid separation [GO:1901970]; positive regulation of protein phosphorylation [GO:0001934]; positive regulation of protein serine/threonine kinase activity [GO:0071902]; protein phosphorylation [GO:0006468]	central element [GO:0000801]; chromocenter [GO:0010369]; chromosome passenger complex [GO:0032133]; chromosome, centromeric region [GO:0000775]; cytosol [GO:0005829]; kinetochore [GO:0000776]; lateral element [GO:0000800]; meiotic spindle midzone [GO:1990385]; microtubule [GO:0005874]; microtubule cytoskeleton [GO:0015630]; midbody [GO:0030496]; nuclear body [GO:0016604]; nucleoplasm [GO:0005654]; nucleus [GO:0005634]; pericentric heterochromatin [GO:0005721]; protein-containing complex [GO:0032991]; spindle [GO:0005819]; protein serine/threonine kinase activator activity [GO:0043539]; chromosome segregation [GO:0007059]; histone phosphorylation [GO:0016572]; meiotic spindle midzone assembly [GO:0051257]; metaphase plate congression [GO:0051310]; mitotic cell cycle [GO:0000278]; mitotic cytokinesis [GO:0000281]; mitotic spindle midzone assembly [GO:0051256]; mitotic spindle organization [GO:0007052]; positive regulation of attachment of mitotic spindle microtubules to kinetochore [GO:1902425]; positive regulation of mitotic cell cycle spindle assembly checkpoint [GO:0090267]; positive regulation of mitotic cytokinesis [GO:1903490]; positive regulation of mitotic sister chromatid separation [GO:1901970];

									positive regulation of protein phosphorylation [GO:0001934]; positive regulation of protein serine/threonine kinase activity [GO:0071902]; protein phosphorylation [GO:0006468]
Q9BW27	NUP85	Nuclear pore complex protein Nup85 OS	75	0.01	-6.64	1.91E-16	15.72	lamellipodium assembly [GO:0030032]; macrophage chemotaxis [GO:0048246]; mRNA export from nucleus [GO:0006406]; nephron development [GO:0072006]; nucleocytoplasmic transport [GO:0006913]; positive regulation of transcription, DNA-templated [GO:0045893]; protein import into nucleus [GO:0006606]	cytosol [GO:0005829]; kinetochore [GO:0000776]; membrane [GO:0016020]; nuclear envelope [GO:0005635]; nuclear membrane [GO:0031965]; nuclear pore [GO:0005643]; nuclear pore outer ring [GO:0031080]; nucleoplasm [GO:0005654]; spindle [GO:0005819]; structural constituent of nuclear pore [GO:0017056]; lamellipodium assembly [GO:0030032]; macrophage chemotaxis [GO:0048246]; mRNA export from nucleus [GO:0006406]; nephron development [GO:0072006]; nucleocytoplasmic transport [GO:0006913]; positive regulation of transcription, DNA-templated [GO:0045893]; protein import into nucleus [GO:0006606]
Q99661	KIF2C	Kinesin-like protein KIF2C OS	81.3	0.01	-6.64	1.91E-16	15.72	attachment of mitotic spindle microtubules to kinetochore [GO:0051315]; cell division [GO:0051301]; establishment or maintenance of microtubule cytoskeleton polarity [GO:0030951]; metaphase plate congression [GO:0051310]; microtubule depolymerization [GO:0007019]; microtubule-based movement [GO:0007018]; mitotic metaphase plate congression [GO:0007080]; regulation of chromosome segregation [GO:0051983]	centrosome [GO:0005813]; chromosome, centromeric region [GO:0000775]; cytosol [GO:0005829]; kinesin complex [GO:0005871]; kinetochore [GO:0000776]; membrane [GO:0016020]; microtubule [GO:0005874]; microtubule cytoskeleton [GO:0015630]; microtubule plus-end [GO:0035371]; nucleus [GO:0005634]; spindle [GO:0005819]; ATP binding [GO:0005524]; ATP hydrolysis activity [GO:0016887]; centromeric DNA binding [GO:0019237]; microtubule binding [GO:0008017]; microtubule motor activity [GO:0003777]; microtubule plus-end binding [GO:0051010]; attachment of mitotic spindle microtubules to kinetochore [GO:0051315]; cell division [GO:0051301]; establishment or maintenance of microtubule cytoskeleton polarity [GO:0030951]; metaphase plate congression [GO:0051310]; microtubule depolymerization

									[GO:0007019]; microtubule-based movement [GO:0007018]; mitotic metaphase plate congression [GO:0007080]; regulation of chromosome segregation [GO:0051983]
Q96CB9	NSUN4	5-methylcytosine rRNA methyltransferase NSUN4 OS	43.1	0.01	-6.64	1.91E-16	15.72	RNA methylation [GO:0001510]; rRNA methylation [GO:0031167]	mitochondrial large ribosomal subunit [GO:0005762]; mitochondrial matrix [GO:0005759]; methyltransferase activity [GO:0008168]; rRNA (cytosine-C5-)-methyltransferase activity [GO:0009383]; rRNA binding [GO:0019843]; RNA methylation [GO:0001510]; rRNA methylation [GO:0031167]
Q9NVM9	INTS13	Integrator complex subunit 13 OS	80.2	0.01	-6.64	1.91E-16	15.72	cell division [GO:0051301]; centrosome localization [GO:0051642]; flagellated sperm motility [GO:0030317]; mitotic spindle organization [GO:0007052]; protein localization to nuclear envelope [GO:0090435]; regulation of fertilization [GO:0080154]; regulation of mitotic cell cycle [GO:0007346]; regulation of transcription elongation from RNA polymerase II promoter [GO:0034243]; snRNA processing [GO:0016180]	cytoplasm [GO:0005737]; integrator complex [GO:0032039]; intracellular membrane-bounded organelle [GO:0043231]; nuclear body [GO:0016604]; nucleoplasm [GO:0005654]; nucleus [GO:0005634]; cell division [GO:0051301]; centrosome localization [GO:0051642]; flagellated sperm motility [GO:0030317]; mitotic spindle organization [GO:0007052]; protein localization to nuclear envelope [GO:0090435]; regulation of fertilization [GO:0080154]; regulation of mitotic cell cycle [GO:0007346]; regulation of transcription elongation from RNA polymerase II promoter [GO:0034243]; snRNA processing [GO:0016180]
P49848	TAF6	Transcription initiation factor TFIID subunit 6 OS	72.6	0.01	-6.64	1.91E-16	15.72	apoptotic process [GO:0006915]; DNA-templated transcription, initiation [GO:0006352]; histone H3 acetylation [GO:0043966]; monoubiquitinated histone deubiquitination [GO:0035521]; monoubiquitinated histone H2A deubiquitination [GO:0035522]; mRNA transcription by RNA polymerase II [GO:0042789]; negative regulation of cell cycle [GO:0045786]; negative regulation of cell population proliferation [GO:0008285]; positive regulation of apoptotic process [GO:0043065]; positive regulation of intrinsic	cytosol [GO:0005829]; MLL1 complex [GO:0071339]; nucleoplasm [GO:0005654]; nucleus [GO:0005634]; protein-containing complex [GO:0032991]; SAGA complex [GO:0000124]; SLIK (SAGA-like) complex [GO:0046695]; transcription factor TFIID complex [GO:0005669]; transcription factor TFTC complex [GO:0033276]; aryl hydrocarbon receptor binding [GO:0017162]; DNA binding [GO:0003677]; protein heterodimerization activity [GO:0046982]; RNA polymerase II general transcription initiation factor activity [GO:0016251]; transcription coactivator activity [GO:0003713]; apoptotic process [GO:0006915]; DNA-templated transcription,

								apoptotic signaling pathway [GO:2001244]; positive regulation of transcription initiation from RNA polymerase II promoter [GO:0060261]; positive regulation of transcription, DNA-templated [GO:0045893]; protein phosphorylation [GO:0006468]; regulation of DNA repair [GO:0006282]; regulation of transcription by RNA polymerase II [GO:0006357]; RNA polymerase II preinitiation complex assembly [GO:0051123]; transcription by RNA polymerase II [GO:0006366]; transcription initiation from RNA polymerase II promoter [GO:0006367]	initiation [GO:0006352]; histone H3 acetylation [GO:0043966]; monoubiquitinated histone deubiquitination [GO:0035521]; monoubiquitinated histone H2A deubiquitination [GO:0035522]; mRNA transcription by RNA polymerase II [GO:0042789]; negative regulation of cell cycle [GO:0045786]; negative regulation of cell population proliferation [GO:0008285]; positive regulation of apoptotic process [GO:0043065]; positive regulation of intrinsic apoptotic signaling pathway [GO:2001244]; positive regulation of transcription initiation from RNA polymerase II promoter [GO:0060261]; positive regulation of transcription, DNA-templated [GO:0045893]; protein phosphorylation [GO:0006468]; regulation of DNA repair [GO:0006282]; regulation of transcription by RNA polymerase II [GO:0006357]; RNA polymerase II preinitiation complex assembly [GO:0051123]; transcription by RNA polymerase II [GO:0006366]; transcription initiation from RNA polymerase II promoter [GO:0006367]
Q9BTC8	MTA3	Metastasis-associated protein MTA3 OS	67.5	0.01	-6.64	1.91E-16	15.72	chromatin remodeling [GO:0006338]; G2/M transition of mitotic cell cycle [GO:0000086]; granulosa cell proliferation [GO:1990739]; histone deacetylation [GO:0016575]; negative regulation of transcription by RNA polymerase II [GO:0000122]; negative regulation of transcription, DNA-templated [GO:0045892]; positive regulation of G2/M transition of mitotic cell cycle [GO:0010971]; positive regulation of granulosa cell proliferation [GO:1904197]; positive regulation of transcription, DNA-templated [GO:0045893]; regulation of cell fate specification [GO:0042659]; regulation of stem cell differentiation [GO:2000736]	cytoplasm [GO:0005737]; intracellular membrane-bounded organelle [GO:0043231]; nucleoplasm [GO:0005654]; NuRD complex [GO:0016581]; chromatin binding [GO:0003682]; histone deacetylase binding [GO:0042826]; metal ion binding [GO:0046872]; NuRD complex binding [GO:0120325]; sequence-specific DNA binding [GO:0043565]; transcription coactivator activity [GO:0003713]; transcription corepressor activity [GO:0003714]; chromatin remodeling [GO:0006338]; G2/M transition of mitotic cell cycle [GO:0000086]; granulosa cell proliferation [GO:1990739]; histone deacetylation [GO:0016575]; negative regulation of transcription by RNA polymerase II [GO:0000122]; negative regulation of transcription, DNA-templated [GO:0045892]; positive regulation of G2/M transition of mitotic cell cycle [GO:0010971]; positive regulation of granulosa cell proliferation [GO:1904197]; positive regulation of transcription, DNA-templated [GO:0045893]; regulation of cell fate

									specification [GO:0042659]; regulation of stem cell differentiation [GO:2000736]
Q6S8J3	POTEE	POTE ankyrin domain family member E OS	121.3	0.01	-6.64	1.91E-16	15.72	retina homeostasis [GO:0001895]; substantia nigra development [GO:0021762]	blood microparticle [GO:0072562]; extracellular exosome [GO:0070062]; extracellular space [GO:0005615]; retina homeostasis [GO:0001895]; substantia nigra development [GO:0021762]
P53365	ARFIP2	Arfaptin-2 OS	37.8	0.01	-6.64	1.91E-16	15.72	actin cytoskeleton organization [GO:0030036]; intracellular protein transport [GO:0006886]; lamellipodium assembly [GO:0030032]; mitophagy [GO:0000423]; protein localization to phagophore assembly site [GO:0034497]; regulation of Arp2/3 complex-mediated actin nucleation [GO:0034315]; ruffle organization [GO:0031529]; small GTPase mediated signal transduction [GO:0007264]	cell cortex [GO:0005938]; cytoplasm [GO:0005737]; cytosol [GO:0005829]; plasma membrane [GO:0005886]; ruffle [GO:0001726]; trans-Golgi network membrane [GO:0032588]; cadherin binding [GO:0045296]; GTP binding [GO:0005525]; GTP-dependent protein binding [GO:0030742]; identical protein binding [GO:0042802]; membrane curvature sensor activity [GO:0140090]; phosphatidylinositol-4-phosphate binding [GO:0070273]; phospholipid binding [GO:0005543]; protein domain specific binding [GO:0019904]; small GTPase binding [GO:0031267]; actin cytoskeleton organization [GO:0030036]; intracellular protein transport [GO:0006886]; lamellipodium assembly [GO:0030032]; mitophagy [GO:0000423]; protein localization to phagophore assembly site [GO:0034497]; regulation of Arp2/3 complex-mediated actin nucleation [GO:0034315]; ruffle organization [GO:0031529]; small GTPase mediated signal transduction [GO:0007264]
Q9NXR7	BABAM2	BRISC and BRCA1-A complex member 2 OS	43.5	0.01	-6.64	1.91E-16	15.72	apoptotic process [GO:0006915]; cell division [GO:0051301]; cellular response to DNA damage stimulus [GO:0006974]; cellular response to ionizing radiation [GO:0071479]; chromatin organization [GO:0006325]; double-strand break repair [GO:0006302]; histone H2A K63-linked deubiquitination [GO:0070537]; histone H2A monoubiquitination [GO:0035518]; mitotic	BRCA1-A complex [GO:0070531]; BRISC complex [GO:0070552]; cytoplasm [GO:0005737]; cytosol [GO:0005829]; nuclear ubiquitin ligase complex [GO:0000152]; nucleoplasm [GO:0005654]; nucleus [GO:0005634]; peroxisome targeting sequence binding [GO:0000268]; polyubiquitin modification-dependent protein binding [GO:0031593]; tumor necrosis factor receptor binding [GO:0005164]; apoptotic process [GO:0006915]; cell division

								G2 DNA damage checkpoint signaling [GO:0007095]; mitotic G2/M transition checkpoint [GO:0044818]; negative regulation of apoptotic process [GO:0043066]; positive regulation of DNA repair [GO:0045739]; protein autoubiquitination [GO:0051865]; protein K63-linked deubiquitination [GO:0070536]; regulation of DNA damage checkpoint [GO:2000001]; regulation of DNA repair [GO:0006282]; response to ionizing radiation [GO:0010212]; signal transduction [GO:0007165]	[GO:0051301]; cellular response to DNA damage stimulus [GO:0006974]; cellular response to ionizing radiation [GO:0071479]; chromatin organization [GO:0006325]; double-strand break repair [GO:0006302]; histone H2A K63-linked deubiquitination [GO:0070537]; histone H2A monoubiquitination [GO:0035518]; mitotic G2 DNA damage checkpoint signaling [GO:0007095]; mitotic G2/M transition checkpoint [GO:0044818]; negative regulation of apoptotic process [GO:0043066]; positive regulation of DNA repair [GO:0045739]; protein autoubiquitination [GO:0051865]; protein K63-linked deubiquitination [GO:0070536]; regulation of DNA damage checkpoint [GO:2000001]; regulation of DNA repair [GO:0006282]; response to ionizing radiation [GO:0010212]; signal transduction [GO:0007165]

P09038	FGF2	Fibroblast growth factor 2 OS	30.8	0.01	-6.64	1.91E-16	15.72	aging [GO:0007568]; angiogenesis involved in coronary vascular morphogenesis [GO:0060978]; animal organ morphogenesis [GO:0009887]; branching involved in ureteric bud morphogenesis [GO:0001658]; canonical Wnt signaling pathway [GO:0060070]; cell differentiation [GO:0030154]; cell migration involved in sprouting angiogenesis [GO:0002042]; cellular response to mechanical stimulus [GO:0071260]; cerebellar granule cell precursor proliferation [GO:0021930]; chemotaxis [GO:0006935]; chondroblast differentiation [GO:0060591]; corticotropin hormone secreting cell differentiation [GO:0060128]; embryo development ending in birth or egg hatching [GO:0009792]; embryonic morphogenesis [GO:0048598]; endothelial cell proliferation [GO:0001935]; ERK1 and ERK2 cascade [GO:0070371]; fibroblast growth factor receptor signaling pathway [GO:0008543]; glial cell differentiation [GO:0010001]; growth factor dependent regulation of skeletal muscle satellite cell proliferation [GO:0014843]; hyaluronan catabolic process [GO:0030214]; inner ear auditory receptor cell differentiation [GO:0042491]; inositol phosphate biosynthetic process [GO:0032958]; lung development [GO:0030324]; lymphatic endothelial cell migration [GO:1904977]; mammary gland epithelial cell differentiation [GO:0060644]; negative regulation of blood vessel endothelial cell migration [GO:0043537]; negative regulation of cell death [GO:0060548]; negative regulation of fibroblast growth factor receptor signaling pathway [GO:0040037]; negative regulation of fibroblast migration [GO:0010764]; negative regulation of gene expression [GO:0010629]; negative regulation of stem	cytoplasm [GO:0005737]; extracellular region [GO:0005576]; extracellular space [GO:0005615]; nucleus [GO:0005634]; chemoattractant activity [GO:0042056]; chemokine binding [GO:0019956]; cytokine activity [GO:0005125]; fibroblast growth factor receptor binding [GO:0005104]; growth factor activity [GO:0008083]; heparin binding [GO:0008201]; identical protein binding [GO:0042802]; integrin binding [GO:0005178]; nuclear receptor coactivator activity [GO:0030374]; receptor-receptor interaction [GO:0090722]; aging [GO:0007568]; angiogenesis involved in coronary vascular morphogenesis [GO:0060978]; animal organ morphogenesis [GO:0009887]; branching involved in ureteric bud morphogenesis [GO:0001658]; canonical Wnt signaling pathway [GO:0060070]; cell differentiation [GO:0030154]; cell migration involved in sprouting angiogenesis [GO:0002042]; cellular response to mechanical stimulus [GO:0071260]; cerebellar granule cell precursor proliferation [GO:0021930]; chemotaxis [GO:0006935]; chondroblast differentiation [GO:0060591]; corticotropin hormone secreting cell differentiation [GO:0060128]; embryo development ending in birth or egg hatching [GO:0009792]; embryonic morphogenesis [GO:0048598]; endothelial cell proliferation [GO:0001935]; ERK1 and ERK2 cascade [GO:0070371]; fibroblast growth factor receptor signaling pathway [GO:0008543]; glial cell differentiation [GO:0010001]; growth factor dependent regulation of skeletal muscle satellite cell proliferation [GO:0014843]; hyaluronan catabolic process [GO:0030214]; inner ear auditory receptor cell differentiation [GO:0042491]; inositol phosphate biosynthetic process [GO:0032958]; lung development [GO:0030324]; lymphatic endothelial cell migration [GO:1904977]; mammary gland epithelial cell differentiation [GO:0060644]; negative regulation of blood vessel endothelial cell migration [GO:0043537]; negative regulation of cell death [GO:0060548]; negative regulation of fibroblast

								cell proliferation [GO:2000647]; negative regulation of wound healing [GO:0061045]; nervous system development [GO:0007399]; neuroblast proliferation [GO:0007405]; organ induction [GO:0001759]; osteoblast differentiation [GO:0001649]; paracrine signaling [GO:0038001]; phosphatidylinositol biosynthetic process [GO:0006661]; positive regulation of angiogenesis [GO:0045766]; positive regulation of blood vessel endothelial cell migration [GO:0043536]; positive regulation of canonical Wnt signaling pathway [GO:0090263]; positive regulation of cardiac muscle cell proliferation [GO:0060045]; positive regulation of cell division [GO:0051781]; positive regulation of cell fate specification [GO:0042660]; positive regulation of cell migration involved in sprouting angiogenesis [GO:0090050]; positive regulation of cell population proliferation [GO:0008284]; positive regulation of cerebellar granule cell precursor proliferation [GO:0021940]; positive regulation of DNA biosynthetic process [GO:2000573]; positive regulation of endothelial cell chemotaxis [GO:2001028]; positive regulation of endothelial cell chemotaxis to fibroblast growth factor [GO:2000546]; positive regulation of endothelial cell migration [GO:0010595]; positive regulation of endothelial cell proliferation [GO:0001938]; positive regulation of epithelial cell proliferation [GO:0050679]; positive regulation of epithelial tube formation [GO:1905278]; positive regulation of ERK1 and ERK2 cascade [GO:0070374]; positive regulation of gene expression [GO:0010628]; positive regulation of inner ear auditory receptor cell differentiation [GO:0045609]; positive regulation of lens fiber cell differentiation	growth factor receptor signaling pathway [GO:0040037]; negative regulation of fibroblast migration [GO:0010764]; negative regulation of gene expression [GO:0010629]; negative regulation of stem cell proliferation [GO:2000647]; negative regulation of wound healing [GO:0061045]; nervous system development [GO:0007399]; neuroblast proliferation [GO:0007405]; organ induction [GO:0001759]; osteoblast differentiation [GO:0001649]; paracrine signaling [GO:0038001]; phosphatidylinositol biosynthetic process [GO:0006661]; positive regulation of angiogenesis [GO:0045766]; positive regulation of blood vessel endothelial cell migration [GO:0043536]; positive regulation of canonical Wnt signaling pathway [GO:0090263]; positive regulation of cardiac muscle cell proliferation [GO:0060045]; positive regulation of cell division [GO:0051781]; positive regulation of cell fate specification [GO:0042660]; positive regulation of cell migration involved in sprouting angiogenesis [GO:0090050]; positive regulation of cell population proliferation [GO:0008284]; positive regulation of cerebellar granule cell precursor proliferation [GO:0021940]; positive regulation of DNA biosynthetic process [GO:2000573]; positive regulation of endothelial cell chemotaxis [GO:2001028]; positive regulation of endothelial cell chemotaxis to fibroblast growth factor [GO:2000546]; positive regulation of endothelial cell migration [GO:0010595]; positive regulation of endothelial cell proliferation [GO:0001938]; positive regulation of epithelial cell proliferation [GO:0050679]; positive regulation of epithelial tube formation [GO:1905278]; positive regulation of ERK1 and ERK2 cascade [GO:0070374]; positive regulation of gene expression [GO:0010628]; positive regulation of inner ear auditory receptor cell differentiation [GO:0045609]; positive regulation of lens fiber cell differentiation [GO:1902748]; positive regulation of MAP kinase activity [GO:0043406]; positive regulation of MAPK cascade [GO:0043410]; positive

								[GO:1902748]; positive regulation of MAP kinase activity [GO:0043406]; positive regulation of MAPK cascade [GO:0043410]; positive regulation of miRNA transcription [GO:1902895]; positive regulation of neuroblast proliferation [GO:0002052]; positive regulation of neuroepithelial cell differentiation [GO:1902913]; positive regulation of osteoblast differentiation [GO:0045669]; positive regulation of phosphatidylinositol 3-kinase activity [GO:0043552]; positive regulation of phosphatidylinositol 3-kinase signaling [GO:0014068]; positive regulation of phospholipase C activity [GO:0010863]; positive regulation of protein kinase B signaling [GO:0051897]; positive regulation of protein phosphorylation [GO:0001934]; positive regulation of sprouting angiogenesis [GO:1903672]; positive regulation of stem cell differentiation [GO:2000738]; positive regulation of stem cell proliferation [GO:2000648]; positive regulation of transcription by RNA polymerase II [GO:0045944]; positive regulation of transcription, DNA-templated [GO:0045893]; positive regulation of vascular associated smooth muscle cell proliferation [GO:1904707]; positive regulation of vascular endothelial cell proliferation [GO:1905564]; protein kinase B signaling [GO:0043491]; Ras protein signal transduction [GO:0007265]; regulation of angiogenesis [GO:0045765]; regulation of blood vessel endothelial cell proliferation involved in sprouting angiogenesis [GO:1903587]; regulation of cell cycle [GO:0051726]; regulation of cell migration [GO:0030334]; regulation of cell migration involved in sprouting angiogenesis [GO:0090049]; regulation of endothelial cell chemotaxis to fibroblast growth factor	regulation of miRNA transcription [GO:1902895]; positive regulation of neuroblast proliferation [GO:0002052]; positive regulation of neuroepithelial cell differentiation [GO:1902913]; positive regulation of osteoblast differentiation [GO:0045669]; positive regulation of phosphatidylinositol 3-kinase activity [GO:0043552]; positive regulation of phosphatidylinositol 3-kinase signaling [GO:0014068]; positive regulation of phospholipase C activity [GO:0010863]; positive regulation of protein kinase B signaling [GO:0051897]; positive regulation of protein phosphorylation [GO:0001934]; positive regulation of sprouting angiogenesis [GO:1903672]; positive regulation of stem cell differentiation [GO:2000738]; positive regulation of stem cell proliferation [GO:2000648]; positive regulation of transcription by RNA polymerase II [GO:0045944]; positive regulation of transcription, DNA-templated [GO:0045893]; positive regulation of vascular associated smooth muscle cell proliferation [GO:1904707]; positive regulation of vascular endothelial cell proliferation [GO:1905564]; protein kinase B signaling [GO:0043491]; Ras protein signal transduction [GO:0007265]; regulation of angiogenesis [GO:0045765]; regulation of blood vessel endothelial cell proliferation involved in sprouting angiogenesis [GO:1903587]; regulation of cell cycle [GO:0051726]; regulation of cell migration [GO:0030334]; regulation of cell migration involved in sprouting angiogenesis [GO:0090049]; regulation of endothelial cell chemotaxis to fibroblast growth factor [GO:2000544]; regulation of retinal cell programmed cell death [GO:0046668]; release of sequestered calcium ion into cytosol [GO:0051209]; response to axon injury [GO:0048678]; signal transduction [GO:0007165]; stem cell development [GO:0048864]; stem cell proliferation [GO:0072089]; substantia nigra development [GO:0021762]; thyroid-stimulating hormone-secreting cell differentiation [GO:0060129]; transcription by RNA

								[GO:2000544]; regulation of retinal cell programmed cell death [GO:0046668]; release of sequestered calcium ion into cytosol [GO:0051209]; response to axon injury [GO:0048678]; signal transduction [GO:0007165]; stem cell development [GO:0048864]; stem cell proliferation [GO:0072089]; substantia nigra development [GO:0021762]; thyroid-stimulating hormone-secreting cell differentiation [GO:0060129]; transcription by RNA polymerase II [GO:0006366]; wound healing [GO:0042060]	polymerase II [GO:0006366]; wound healing [GO:0042060]
O43598	DNPH1	2'-deoxynucleoside 5'-phosphate N-hydrolase 1 OS	19.1	0.01	-6.64	1.91E-16	15.72	deoxyribonucleoside monophosphate catabolic process [GO:0009159]; epithelial cell differentiation [GO:0030855]; nucleoside metabolic process [GO:0009116]; positive regulation of cell growth [GO:0030307];	cytosol [GO:0005829]; extracellular exosome [GO:0070062]; nucleus [GO:0005634]; deoxyribonucleoside 5'-monophosphate N-glycosidase activity [GO:0070694]; identical protein binding [GO:0042802]; protein homodimerization activity [GO:0042803]; deoxyribonucleoside

								purine nucleotide catabolic process [GO:0006195]	monophosphate catabolic process [GO:0009159]; epithelial cell differentiation [GO:0030855]; nucleoside metabolic process [GO:0009116]; positive regulation of cell growth [GO:0030307]; purine nucleotide catabolic process [GO:0006195]
Q9BY76	ANGPTL4	Angiopoietin-related protein 4 OS	45.2	0.01	-6.64	1.91E-16	15.72	angiogenesis [GO:0001525]; endothelial cell apoptotic process [GO:0072577]; lipid metabolic process [GO:0006629]; negative regulation of apoptotic process [GO:0043066]; negative regulation of endothelial cell apoptotic process [GO:2000352]; negative regulation of lipoprotein lipase activity [GO:0051005]; positive regulation of angiogenesis [GO:0045766]; protein unfolding [GO:0043335]; response to hypoxia [GO:0001666]; triglyceride homeostasis [GO:0070328]	blood microparticle [GO:0072562]; collagen-containing extracellular matrix [GO:0062023]; extracellular region [GO:0005576]; extracellular space [GO:0005615]; enzyme inhibitor activity [GO:0004857]; identical protein binding [GO:0042802]; signaling receptor binding [GO:0005102]; angiogenesis [GO:0001525]; endothelial cell apoptotic process [GO:0072577]; lipid metabolic process [GO:0006629]; negative regulation of apoptotic process [GO:0043066]; negative regulation of endothelial cell apoptotic process [GO:2000352]; negative regulation of lipoprotein lipase activity [GO:0051005]; positive regulation of angiogenesis [GO:0045766]; protein unfolding [GO:0043335]; response to hypoxia [GO:0001666]; triglyceride homeostasis [GO:0070328]

Q92769	HDAC2	Histone deacetylase 2 OS	55.3	0.01	-6.64	1.91E-16	15.72	behavioral response to ethanol [GO:0048149]; cardiac muscle hypertrophy [GO:0003300]; cellular response to dopamine [GO:1903351]; cellular response to heat [GO:0034605]; cellular response to hydrogen peroxide [GO:0070301]; cellular response to retinoic acid [GO:0071300]; cellular response to transforming growth factor beta stimulus [GO:0071560]; chromatin remodeling [GO:0006338]; circadian regulation of gene expression [GO:0032922]; dendrite development [GO:0016358]; embryonic digit morphogenesis [GO:0042733]; epidermal cell differentiation [GO:0009913]; eyelid development in camera-type eye [GO:0061029]; fungiform papilla formation [GO:0061198]; hair follicle placode formation [GO:0060789]; heterochromatin organization [GO:0070828]; histone deacetylation [GO:0016575]; histone H3 deacetylation [GO:0070932]; histone H4 deacetylation [GO:0070933]; negative regulation of apoptotic process [GO:0043066]; negative regulation of cell migration [GO:0030336]; negative regulation of dendritic spine development [GO:0061000]; negative regulation of DNA binding [GO:0043392]; negative regulation of DNA-binding transcription factor activity [GO:0043433]; negative regulation of MHC class II biosynthetic process [GO:0045347]; negative regulation of neuron projection development [GO:0010977]; negative regulation of peptidyl-lysine acetylation [GO:2000757]; negative regulation of stem cell population maintenance [GO:1902455]; negative regulation of transcription by RNA polymerase II [GO:0000122]; negative regulation of transcription, DNA-templated [GO:0045892]; negative regulation of transforming growth factor beta receptor	chromatin [GO:0000785]; chromosome, telomeric region [GO:0000781]; cytoplasm [GO:0005737]; ESC/E(Z) complex [GO:0035098]; histone deacetylase complex [GO:0000118]; nucleoplasm [GO:0005654]; nucleus [GO:0005634]; NuRD complex [GO:0016581]; protein-containing complex [GO:0032991]; Sin3 complex [GO:0016580]; chromatin binding [GO:0003682]; deacetylase activity [GO:0019213]; enzyme binding [GO:0019899]; heat shock protein binding [GO:0031072]; histone deacetylase activity [GO:0004407]; histone deacetylase binding [GO:0042826]; histone decrotonylase activity [GO:0160009]; NF-kappaB binding [GO:0051059]; promoter-specific chromatin binding [GO:1990841]; protein de-2-hydroxyisobutyrylase activity [GO:0160010]; protein lysine deacetylase activity [GO:0033558]; RNA binding [GO:0003723]; RNA polymerase II-specific DNA-binding transcription factor binding [GO:0061629]; sequence-specific DNA binding [GO:0043565]; behavioral response to ethanol [GO:0048149]; cardiac muscle hypertrophy [GO:0003300]; cellular response to dopamine [GO:1903351]; cellular response to heat [GO:0034605]; cellular response to hydrogen peroxide [GO:0070301]; cellular response to retinoic acid [GO:0071300]; cellular response to transforming growth factor beta stimulus [GO:0071560]; chromatin remodeling [GO:0006338]; circadian regulation of gene expression [GO:0032922]; dendrite development [GO:0016358]; embryonic digit morphogenesis [GO:0042733]; epidermal cell differentiation [GO:0009913]; eyelid development in camera-type eye [GO:0061029]; fungiform papilla formation [GO:0061198]; hair follicle placode formation [GO:0060789]; heterochromatin organization [GO:0070828]; histone deacetylation [GO:0016575]; histone H3 deacetylation [GO:0070932]; histone H4 deacetylation [GO:0070933]; negative regulation of apoptotic process [GO:0043066]; negative regulation of cell

								signaling pathway [GO:0030512]; odontogenesis of dentin-containing tooth [GO:0042475]; positive regulation of cell population proliferation [GO:0008284]; positive regulation of collagen biosynthetic process [GO:0032967]; positive regulation of epithelial to mesenchymal transition [GO:0010718]; positive regulation of interleukin-1 production [GO:0032732]; positive regulation of male mating behavior [GO:1902437]; positive regulation of oligodendrocyte differentiation [GO:0048714]; positive regulation of proteolysis [GO:0045862]; positive regulation of signaling receptor activity [GO:2000273]; positive regulation of stem cell population maintenance [GO:1902459]; positive regulation of transcription by RNA polymerase II [GO:0045944]; positive regulation of transcription, DNA-templated [GO:0045893]; positive regulation of tumor necrosis factor production [GO:0032760]; positive regulation of tyrosine phosphorylation of STAT protein [GO:0042531]; regulation of cell fate specification [GO:0042659]; regulation of stem cell differentiation [GO:2000736]; regulation of transcription by RNA polymerase II [GO:0006357]; response to amphetamine [GO:0001975]; response to caffeine [GO:0031000]; response to cocaine [GO:0042220]; response to hyperoxia [GO:0055093]; response to lipopolysaccharide [GO:0032496]; response to nicotine [GO:0035094]; response to xenobiotic stimulus [GO:0009410]	migration [GO:0030336]; negative regulation of dendritic spine development [GO:0061000]; negative regulation of DNA binding [GO:0043392]; negative regulation of DNA-binding transcription factor activity [GO:0043433]; negative regulation of MHC class II biosynthetic process [GO:0045347]; negative regulation of neuron projection development [GO:0010977]; negative regulation of peptidyl-lysine acetylation [GO:2000757]; negative regulation of stem cell population maintenance [GO:1902455]; negative regulation of transcription by RNA polymerase II [GO:0000122]; negative regulation of transcription, DNA-templated [GO:0045892]; negative regulation of transforming growth factor beta receptor signaling pathway [GO:0030512]; odontogenesis of dentin-containing tooth [GO:0042475]; positive regulation of cell population proliferation [GO:0008284]; positive regulation of collagen biosynthetic process [GO:0032967]; positive regulation of epithelial to mesenchymal transition [GO:0010718]; positive regulation of interleukin-1 production [GO:0032732]; positive regulation of male mating behavior [GO:1902437]; positive regulation of oligodendrocyte differentiation [GO:0048714]; positive regulation of proteolysis [GO:0045862]; positive regulation of signaling receptor activity [GO:2000273]; positive regulation of stem cell population maintenance [GO:1902459]; positive regulation of transcription by RNA polymerase II [GO:0045944]; positive regulation of transcription, DNA-templated [GO:0045893]; positive regulation of tumor necrosis factor production [GO:0032760]; positive regulation of tyrosine phosphorylation of STAT protein [GO:0042531]; regulation of cell fate specification [GO:0042659]; regulation of stem cell differentiation [GO:2000736]; regulation of transcription by RNA polymerase II [GO:0006357]; response to amphetamine [GO:0001975]; response to caffeine [GO:0031000]; response to cocaine [GO:0042220]; response to hyperoxia [GO:0055093]; response to

									lipopolysaccharide [GO:0032496]; response to nicotine [GO:0035094]; response to xenobiotic stimulus [GO:0009410]
Q9NXH8	TOR4A	Torsin-4A OS	46.9	0.01	-6.64	1.91E-16	15.72		endoplasmic reticulum lumen [GO:0005788]; extracellular region [GO:0005576]; integral component of membrane [GO:0016021]; nuclear envelope [GO:0005635]; platelet alpha granule lumen [GO:0031093]; ATP binding [GO:0005524]; ATP hydrolysis activity [GO:0016887]
O15228	GNPAT	Dihydroxyacetone phosphate acyltransferase OS	77.1	0.01	-6.64	1.91E-16	15.72	cerebellum morphogenesis [GO:0021587]; ether lipid biosynthetic process [GO:0008611]; fatty acid metabolic process [GO:0006631]; membrane organization [GO:0061024]; paranodal junction assembly [GO:0030913]; phosphatidic acid biosynthetic process [GO:0006654]; phospholipid biosynthetic process [GO:0008654]; response to fatty acid [GO:0070542]; response to nutrient [GO:0007584]; response to	cytosol [GO:0005829]; membrane [GO:0016020]; mitochondrial membrane [GO:0031966]; peroxisomal matrix [GO:0005782]; peroxisomal membrane [GO:0005778]; peroxisome [GO:0005777]; glycerone-phosphate O-acyltransferase activity [GO:0016287]; cerebellum morphogenesis [GO:0021587]; ether lipid biosynthetic process [GO:0008611]; fatty acid metabolic process [GO:0006631]; membrane organization [GO:0061024]; paranodal junction

								starvation [GO:0042594]; response to xenobiotic stimulus [GO:0009410]; synapse assembly [GO:0007416]	assembly [GO:0030913]; phosphatidic acid biosynthetic process [GO:0006654]; phospholipid biosynthetic process [GO:0008654]; response to fatty acid [GO:0070542]; response to nutrient [GO:0007584]; response to starvation [GO:0042594]; response to xenobiotic stimulus [GO:0009410]; synapse assembly [GO:0007416]
Q86WB0	ZC3HC1	Nuclear-interacting partner of ALK OS	55.2	0.01	-6.64	1.91E-16	15.72	cell cycle [GO:0007049]; cell division [GO:0051301]; negative regulation of extrinsic apoptotic signaling pathway in absence of ligand [GO:2001240]; protein ubiquitination [GO:0016567]	nuclear membrane [GO:0031965]; nucleoplasm [GO:0005654]; nucleus [GO:0005634]; protein kinase binding [GO:0019901]; zinc ion binding [GO:0008270]; cell cycle [GO:0007049]; cell division [GO:0051301]; negative regulation of extrinsic apoptotic signaling pathway in absence of ligand [GO:2001240]; protein ubiquitination [GO:0016567]
P26358	DNMT1	DNA (cytosine-5)-methyltransferase 1 OS	183.1	0.01	-6.64	1.91E-16	15.72	cellular response to amino acid stimulus [GO:0071230]; cellular response to bisphenol A [GO:1903926]; DNA methylation [GO:0006306]; DNA methylation involved in embryo development [GO:0043045]; DNA methylation-dependent heterochromatin assembly [GO:0006346]; maintenance of DNA methylation [GO:0010216]; negative regulation of gene expression [GO:0010629]; negative regulation of histone H3-K9 methylation [GO:0051573]; negative regulation of transcription by RNA polymerase II [GO:0000122]; negative regulation of vascular associated smooth muscle cell apoptotic process [GO:1905460]; negative regulation of vascular associated smooth muscle cell differentiation involved in phenotypic switching [GO:1905931]; positive regulation of DNA methylation-dependent heterochromatin assembly [GO:0090309]; positive regulation of gene expression [GO:0010628]; positive regulation of histone H3-K4 methylation [GO:0051571]; positive regulation of vascular associated smooth muscle cell proliferation [GO:1904707]; Ras	female germ cell nucleus [GO:0001674]; nucleoplasm [GO:0005654]; nucleus [GO:0005634]; pericentric heterochromatin [GO:0005721]; replication fork [GO:0005657]; DNA (cytosine-5-)-methyltransferase activity [GO:0003886]; DNA binding [GO:0003677]; DNA-methyltransferase activity [GO:0009008]; methyl-CpG binding [GO:0008327]; promoter-specific chromatin binding [GO:1990841]; RNA binding [GO:0003723]; zinc ion binding [GO:0008270]; cellular response to amino acid stimulus [GO:0071230]; cellular response to bisphenol A [GO:1903926]; DNA methylation [GO:0006306]; DNA methylation involved in embryo development [GO:0043045]; DNA methylation-dependent heterochromatin assembly [GO:0006346]; maintenance of DNA methylation [GO:0010216]; negative regulation of gene expression [GO:0010629]; negative regulation of histone H3-K9 methylation [GO:0051573]; negative regulation of transcription by RNA polymerase II [GO:0000122]; negative regulation of vascular associated smooth muscle cell apoptotic process [GO:1905460]; negative regulation of vascular associated smooth muscle cell differentiation involved in phenotypic switching [GO:1905931]; positive regulation of DNA methylation-dependent

								protein signal transduction [GO:0007265]; transcription, DNA-templated [GO:0006351]	heterochromatin assembly [GO:0090309]; positive regulation of gene expression [GO:0010628]; positive regulation of histone H3-K4 methylation [GO:0051571]; positive regulation of vascular associated smooth muscle cell proliferation [GO:1904707]; Ras protein signal transduction [GO:0007265]; transcription, DNA-templated [GO:0006351]
Q5TAQ9	DCAF8	DDB1- and CUL4-associated factor 8 OS	66.8	0.01	-6.64	1.91E-16	15.72	protein ubiquitination [GO:0016567]	Cul4-RING E3 ubiquitin ligase complex [GO:0080008]; cytoplasm [GO:0005737]; cytosol [GO:0005829]; mitochondrion [GO:0005739]; nucleoplasm [GO:0005654]; nucleus [GO:0005634]; protein ubiquitination [GO:0016567]
Q9Y333	LSM2	U6 snRNA-associated Sm-like protein LSm2 OS	10.8	0.01	-6.64	1.91E-16	15.72	mRNA catabolic process [GO:0006402]; mRNA splicing, via spliceosome [GO:0000398]; spliceosomal tri-snRNP complex assembly [GO:0000244]	catalytic step 2 spliceosome [GO:0071013]; cytoplasm [GO:0005737]; cytosol [GO:0005829]; Lsm1-7-Pat1 complex [GO:1990726]; Lsm2-8 complex [GO:0120115]; nucleoplasm [GO:0005654]; nucleus [GO:0005634]; P-body [GO:0000932]; precatalytic spliceosome [GO:0071011]; U2-type precatalytic spliceosome [GO:0071005]; U4/U6 x U5 tri-snRNP complex [GO:0046540]; U6 snRNP [GO:0005688]; RNA binding [GO:0003723]; small GTPase binding [GO:0031267]; U6 snRNA binding [GO:0017070]; mRNA catabolic process [GO:0006402]; mRNA splicing, via spliceosome [GO:0000398]; spliceosomal tri-snRNP complex assembly [GO:0000244]
O95639	CPSF4	Cleavage and polyadenylation specificity factor subunit 4 OS	30.2	0.01	-6.64	1.91E-16	15.72	pre-mRNA cleavage required for polyadenylation [GO:0098789]	intracellular membrane-bounded organelle [GO:0043231]; mRNA cleavage and polyadenylation specificity factor complex [GO:0005847]; nucleoplasm [GO:0005654]; RNA binding [GO:0003723]; sequence-specific double-stranded DNA binding [GO:1990837]; zinc ion binding [GO:0008270]; pre-mRNA cleavage required for polyadenylation [GO:0098789]

O75940	SMNDC1	Survival of motor neuron-related-splicing factor 30 OS	26.7	0.01	-6.64	1.91E-16	15.72	apoptotic process [GO:0006915]; mRNA processing [GO:0006397]; RNA splicing, via transesterification reactions [GO:0000375]	Cajal body [GO:0015030]; cytoplasm [GO:0005737]; nuclear speck [GO:0016607]; nucleoplasm [GO:0005654]; nucleus [GO:0005634]; spliceosomal complex [GO:0005681]; RNA binding [GO:0003723]; apoptotic process [GO:0006915]; mRNA processing [GO:0006397]; RNA splicing, via transesterification reactions [GO:0000375]
Q9Y450	HBS1L	HBS1-like protein OS	75.4	0.01	-6.64	1.91E-16	15.72	signal transduction [GO:0007165]; translation [GO:0006412]	cytosol [GO:0005829]; extracellular exosome [GO:0070062]; membrane [GO:0016020]; GTP binding [GO:0005525]; GTPase activity [GO:0003924]; translation elongation factor activity [GO:0003746]; signal transduction [GO:0007165]; translation [GO:0006412]
Q969S9	GFM2	Ribosome-releasing factor 2, mitochondrial OS	86.5	0.01	-6.64	1.91E-16	15.72	mitochondrial translation [GO:0032543]; mitochondrial translational termination [GO:0070126]; ribosome disassembly [GO:0032790]	mitochondrial matrix [GO:0005759]; GTP binding [GO:0005525]; GTPase activity [GO:0003924]; mitochondrial translation [GO:0032543]; mitochondrial translational termination [GO:0070126]; ribosome disassembly [GO:0032790]
Q9Y6G3	MRPL42	39S ribosomal protein L42, mitochondrial OS	16.7	0.01	-6.64	1.91E-16	15.72	mitochondrial translation [GO:0032543]; translation [GO:0006412]	mitochondrial inner membrane [GO:0005743]; mitochondrial large ribosomal subunit [GO:0005762]; mitochondrial small ribosomal subunit [GO:0005763]; mitochondrion [GO:0005739]; plasma membrane [GO:0005886]; RNA binding [GO:0003723]; structural constituent of ribosome [GO:0003735]; mitochondrial translation [GO:0032543]; translation [GO:0006412]
Q9H2W6	MRPL46	39S ribosomal protein L46, mitochondrial OS	31.7	0.01	-6.64	1.91E-16	15.72	mitochondrial translation [GO:0032543]	cell junction [GO:0030054]; mitochondrial inner membrane [GO:0005743]; mitochondrial large ribosomal subunit [GO:0005762]; mitochondrion [GO:0005739]; nucleoplasm [GO:0005654]; structural constituent of ribosome [GO:0003735]; mitochondrial translation [GO:0032543]
P82664	MRPS10	28S ribosomal protein S10, mitochondrial OS	23	0.01	-6.64	1.91E-16	15.72	mitochondrial translation [GO:0032543]	mitochondrial inner membrane [GO:0005743]; mitochondrial small ribosomal subunit [GO:0005763]; mitochondrion [GO:0005739]; mitochondrial translation [GO:0032543]

Q9NRK6	ABCB10	ATP-binding cassette sub-family B member 10, mitochondrial OS	79.1	0.01	-6.64	1.91E-16	15.72	erythrocyte development [GO:0048821]; heme biosynthetic process [GO:0006783]; mitochondrial transport [GO:0006839]; mitochondrial unfolded protein response [GO:0034514]; positive regulation of erythrocyte differentiation [GO:0045648]; positive regulation of heme biosynthetic process [GO:0070455]; positive regulation of hemoglobin biosynthetic process [GO:0046985]	integral component of mitochondrial membrane [GO:0032592]; mitochondrial inner membrane [GO:0005743]; mitochondrion [GO:0005739]; ABC-type transporter activity [GO:0140359]; ATP binding [GO:0005524]; ATP hydrolysis activity [GO:0016887]; protein homodimerization activity [GO:0042803]; erythrocyte development [GO:0048821]; heme biosynthetic process [GO:0006783]; mitochondrial transport [GO:0006839]; mitochondrial unfolded protein response [GO:0034514]; positive regulation of erythrocyte differentiation [GO:0045648]; positive regulation of heme biosynthetic process [GO:0070455]; positive regulation of hemoglobin biosynthetic process [GO:0046985]
Q9BUL9	RPP25	Ribonuclease P protein subunit p25 OS	20.6	0.01	-6.64	1.91E-16	15.72	rRNA processing [GO:0006364]; tRNA 5'-leader removal [GO:0001682]	centriolar satellite [GO:0034451]; multimeric ribonuclease P complex [GO:0030681]; nucleolus [GO:0005730]; nucleoplasm [GO:0005654]; ribonuclease MRP complex [GO:0000172]; ribonuclease P activity [GO:0004526]; ribonuclease P RNA binding [GO:0033204]; RNA binding [GO:0003723]; rRNA processing [GO:0006364]; tRNA 5'-leader removal [GO:0001682]
Q9BVI4	NOC4L	Nucleolar complex protein 4 homolog OS	58.4	0.01	-6.64	1.91E-16	15.72	rRNA processing [GO:0006364]	integral component of membrane [GO:0016021]; Noc4p-Nop14p complex [GO:0030692]; nuclear membrane [GO:0031965]; nucleolus [GO:0005730]; nucleoplasm [GO:0005654]; small-subunit processome [GO:0032040]; RNA binding [GO:0003723]; rRNA processing [GO:0006364]
Q9NRX1	PNO1	RNA-binding protein PNO1 OS	27.9	0.01	-6.64	1.91E-16	15.72		cytosol [GO:0005829]; nucleolus [GO:0005730]; nucleoplasm [GO:0005654]; RNA binding [GO:0003723]
Q9NQZ2	UTP3	Something about silencing protein 10 OS	54.5	0.01	-6.64	1.91E-16	15.72	brain development [GO:0007420]; chromatin organization [GO:0006325]; maturation of SSU-rRNA from tricistronic rRNA transcript (SSU-rRNA, 5.8S rRNA, LSU-rRNA) [GO:0000462]	nucleolus [GO:0005730]; nucleoplasm [GO:0005654]; nucleus [GO:0005634]; small-subunit processome [GO:0032040]; RNA binding [GO:0003723]; brain development [GO:0007420]; chromatin organization [GO:0006325]; maturation of SSU-rRNA from tricistronic rRNA transcript (SSU-rRNA, 5.8S rRNA, LSU-rRNA) [GO:0000462]

P17812	CTPS1	CTP synthase 1 OS	66.6	0.01	-6.64	1.91E-16	15.72	'de novo' CTP biosynthetic process [GO:0044210]; B cell proliferation [GO:0042100]; CTP biosynthetic process [GO:0006241]; glutamine metabolic process [GO:0006541]; nucleobase-containing compound metabolic process [GO:0006139]; pyrimidine nucleobase biosynthetic process [GO:0019856]; response to xenobiotic stimulus [GO:0009410]; T cell proliferation [GO:0042098]	cytoophidium [GO:0097268]; cytoplasm [GO:0005737]; cytosol [GO:0005829]; membrane [GO:0016020]; ATP binding [GO:0005524]; CTP synthase activity [GO:0003883]; identical protein binding [GO:0042802]; 'de novo' CTP biosynthetic process [GO:0044210]; B cell proliferation [GO:0042100]; CTP biosynthetic process [GO:0006241]; glutamine metabolic process [GO:0006541]; nucleobase-containing compound metabolic process [GO:0006139]; pyrimidine nucleobase biosynthetic process [GO:0019856]; response to xenobiotic stimulus [GO:0009410]; T cell proliferation [GO:0042098]
P50579	METAP2	Methionine aminopeptidase 2 OS	52.9	0.01	-6.64	1.91E-16	15.72	N-terminal protein amino acid modification [GO:0031365]; peptidyl-methionine modification [GO:0018206]; protein initiator methionine removal involved in protein maturation [GO:0035551]; protein processing [GO:0016485]	cytoplasm [GO:0005737]; cytosol [GO:0005829]; plasma membrane [GO:0005886]; aminopeptidase activity [GO:0004177]; metal ion binding [GO:0046872]; metalloaminopeptidase activity [GO:0070006]; metalloexopeptidase activity [GO:0008235]; RNA binding [GO:0003723]; N-terminal protein amino acid modification [GO:0031365]; peptidyl-methionine modification [GO:0018206]; protein initiator methionine removal involved in protein maturation [GO:0035551]; protein processing [GO:0016485]
Q9UBL6	CPNE7	Copine-7 OS	70.2	0.01	-6.64	1.91E-16	15.72	cellular response to calcium ion [GO:0071277]; lipid metabolic process [GO:0006629]	cytoplasm [GO:0005737]; extracellular exosome [GO:0070062]; nucleus [GO:0005634]; plasma membrane [GO:0005886]; calcium-dependent phospholipid binding [GO:0005544]; metal ion binding [GO:0046872]; cellular response to calcium ion [GO:0071277]; lipid metabolic process [GO:0006629]
P03928	MT-ATP8	ATP synthase protein 8 OS	8	0.01	-6.64	1.91E-16	15.72	proton motive force-driven ATP synthesis [GO:0015986]; proton motive force-driven mitochondrial ATP synthesis [GO:0042776]	integral component of membrane [GO:0016021]; mitochondrial inner membrane [GO:0005743]; mitochondrial proton-transporting ATP synthase complex [GO:0005753]; mitochondrial proton-transporting ATP synthase complex, coupling factor F(o) [GO:0000276]; proton transmembrane transporter activity [GO:0015078]; proton motive force-driven ATP synthesis [GO:0015986]; proton

									motive force-driven mitochondrial ATP synthesis [GO:0042776]
P84085	ARF5	ADP-ribosylation factor 5 OS	20.5	0.01	-6.64	1.91E-16	15.72	intracellular protein transport [GO:0006886]; retrograde vesicle-mediated transport, Golgi to endoplasmic reticulum [GO:0006890]; vesicle-mediated transport [GO:0016192]	extracellular exosome [GO:0070062]; Golgi apparatus [GO:0005794]; perinuclear region of cytoplasm [GO:0048471]; plasma membrane [GO:0005886]; GTP binding [GO:0005525]; GTPase activity [GO:0003924]; intracellular protein transport [GO:0006886]; retrograde vesicle-mediated transport, Golgi to endoplasmic reticulum [GO:0006890]; vesicle-mediated transport [GO:0016192]
Q8NI22	MCFD2	Multiple coagulation factor deficiency protein 2 OS	16.4	0.01	-6.64	1.91E-16	15.72	protein transport [GO:0015031]; vesicle-mediated transport [GO:0016192]	endoplasmic reticulum membrane [GO:0005789]; endoplasmic reticulum-Golgi intermediate compartment membrane [GO:0033116]; ER to Golgi transport vesicle membrane [GO:0012507]; Golgi apparatus [GO:0005794]; calcium ion binding [GO:0005509]; protein transport [GO:0015031]; vesicle-mediated transport [GO:0016192]
Q9H0H5	RACGAP1	Rac GTPase-activating protein 1 OS	71	0.01	-6.64	1.91E-16	15.72	actomyosin contractile ring assembly [GO:0000915]; mitotic cytokinesis [GO:0000281]; mitotic spindle midzone assembly [GO:0051256]; neuroblast proliferation [GO:0007405]; positive regulation of cytokinesis [GO:0032467]; regulation of attachment of spindle microtubules to kinetochore [GO:0051988]; regulation of embryonic development [GO:0045995]; regulation of small GTPase mediated signal transduction [GO:0051056]; Rho protein signal transduction [GO:0007266]; spermatogenesis [GO:0007283]; sulfate transport [GO:0008272]	acrosomal vesicle [GO:0001669]; centralspindlin complex [GO:0097149]; cleavage furrow [GO:0032154]; cytosol [GO:0005829]; extracellular exosome [GO:0070062]; extrinsic component of cytoplasmic side of plasma membrane [GO:0031234]; Flemming body [GO:0090543]; microtubule [GO:0005874]; midbody [GO:0030496]; mitotic spindle [GO:0072686]; nucleoplasm [GO:0005654]; nucleus [GO:0005634]; spindle [GO:0005819]; spindle midzone [GO:0051233]; alpha-tubulin binding [GO:0043014]; beta-tubulin binding [GO:0048487]; gamma-tubulin binding [GO:0043015]; GTPase activator activity [GO:0005096]; metal ion binding [GO:0046872]; microtubule binding [GO:0008017]; phosphatidylinositol-3,4,5-trisphosphate binding [GO:0005547]; protein kinase binding [GO:0019901]; actomyosin contractile ring assembly [GO:0000915]; mitotic cytokinesis [GO:0000281]; mitotic spindle midzone assembly [GO:0051256]; neuroblast proliferation [GO:0007405]; positive regulation of

									cytokinesis [GO:0032467]; regulation of attachment of spindle microtubules to kinetochore [GO:0051988]; regulation of embryonic development [GO:0045995]; regulation of small GTPase mediated signal transduction [GO:0051056]; Rho protein signal transduction [GO:0007266]; spermatogenesis [GO:0007283]; sulfate transport [GO:0008272]
Q9BW19	KIFC1	Kinesin-like protein KIFC1 OS	73.7	0.01	-6.64	1.91E-16	15.72	cell division [GO:0051301]; microtubule-based movement [GO:0007018]; mitotic metaphase plate congression [GO:0007080]; mitotic sister chromatid segregation [GO:0000070]; mitotic spindle assembly [GO:0090307]	early endosome [GO:0005769]; kinesin complex [GO:0005871]; membrane [GO:0016020]; microtubule [GO:0005874]; microtubule organizing center [GO:0005815]; mitotic spindle [GO:0072686]; nucleus [GO:0005634]; ATP binding [GO:0005524]; ATP hydrolysis activity [GO:0016887]; microtubule binding [GO:0008017]; microtubule motor activity [GO:0003777]; cell division [GO:0051301]; microtubule-based movement [GO:0007018]; mitotic metaphase plate congression [GO:0007080]; mitotic sister chromatid segregation [GO:0000070]; mitotic spindle assembly [GO:0090307]
Q13443	ADAM9	Disintegrin and metalloproteinase domain-containing protein 9 OS	90.5	0.01	-6.64	1.91E-16	15.72	amyloid precursor protein catabolic process [GO:0042987]; cell adhesion [GO:0007155]; cell adhesion mediated by integrin [GO:0033627]; cell migration [GO:0016477]; cell-cell adhesion mediated by integrin [GO:0033631]; cell-matrix adhesion [GO:0007160]; cellular response to lipopolysaccharide [GO:0071222]; integrin-mediated signaling pathway [GO:0007229]; keratinocyte differentiation [GO:0030216]; membrane protein ectodomain proteolysis [GO:0006509]; membrane protein intracellular domain proteolysis [GO:0031293]; monocyte activation [GO:0042117]; positive regulation of cell adhesion mediated by integrin [GO:0033630]; positive regulation of cell migration [GO:0030335]; positive regulation of keratinocyte migration [GO:0051549]; positive regulation of macrophage fusion [GO:0034241]; positive regulation of MAP	cell surface [GO:0009986]; extracellular exosome [GO:0070062]; extracellular space [GO:0005615]; focal adhesion [GO:0005925]; integral component of membrane [GO:0016021]; intrinsic component of external side of plasma membrane [GO:0031233]; collagen binding [GO:0005518]; integrin binding [GO:0005178]; laminin binding [GO:0043236]; metal ion binding [GO:0046872]; metalloendopeptidase activity [GO:0004222]; metalloendopeptidase activity involved in amyloid precursor protein catabolic process [GO:1902945]; metallopeptidase activity [GO:0008237]; protein kinase C binding [GO:0005080]; SH3 domain binding [GO:0017124]; amyloid precursor protein catabolic process [GO:0042987]; cell adhesion [GO:0007155]; cell adhesion mediated by integrin [GO:0033627]; cell migration [GO:0016477]; cell-cell adhesion mediated by integrin [GO:0033631]; cell-matrix adhesion [GO:0007160]; cellular response to lipopolysaccharide [GO:0071222]; integrin-mediated signaling pathway [GO:0007229]; keratinocyte

								kinase activity [GO:0043406]; positive regulation of membrane protein ectodomain proteolysis [GO:0051044]; positive regulation of protein secretion [GO:0050714]; protein processing [GO:0016485]; response to calcium ion [GO:0051592]; response to glucocorticoid [GO:0051384]; response to hydrogen peroxide [GO:0042542]; response to manganese ion [GO:0010042]; response to tumor necrosis factor [GO:0034612]; transforming growth factor beta receptor signaling pathway [GO:0007179]	differentiation [GO:0030216]; membrane protein ectodomain proteolysis [GO:0006509]; membrane protein intracellular domain proteolysis [GO:0031293]; monocyte activation [GO:0042117]; positive regulation of cell adhesion mediated by integrin [GO:0033630]; positive regulation of cell migration [GO:0030335]; positive regulation of keratinocyte migration [GO:0051549]; positive regulation of macrophage fusion [GO:0034241]; positive regulation of MAP kinase activity [GO:0043406]; positive regulation of membrane protein ectodomain proteolysis [GO:0051044]; positive regulation of protein secretion [GO:0050714]; protein processing [GO:0016485]; response to calcium ion [GO:0051592]; response to glucocorticoid [GO:0051384]; response to hydrogen peroxide [GO:0042542]; response to manganese ion [GO:0010042]; response to tumor necrosis factor [GO:0034612]; transforming growth factor beta receptor signaling pathway [GO:0007179]
Q75T13	PGAP1	GPI inositol-deacylase OS	105.3	0.01	-6.64	1.91E-16	15.72	anterior/posterior axis specification [GO:0009948]; attachment of GPI anchor to protein [GO:0016255]; embryonic pattern specification [GO:0009880]; endoplasmic reticulum to Golgi vesicle-mediated transport [GO:0006888]; forebrain regionalization [GO:0021871]; GPI anchor metabolic process [GO:0006505]; positive regulation of ER to Golgi vesicle-mediated transport [GO:1902953]; protein transport [GO:0015031]; sensory perception of sound [GO:0007605]	endoplasmic reticulum [GO:0005783]; endoplasmic reticulum membrane [GO:0005789]; integral component of membrane [GO:0016021]; hydrolase activity, acting on ester bonds [GO:0016788]; phosphatidylinositol deacylase activity [GO:0050185]; anterior/posterior axis specification [GO:0009948]; attachment of GPI anchor to protein [GO:0016255]; embryonic pattern specification [GO:0009880]; endoplasmic reticulum to Golgi vesicle-mediated transport [GO:0006888]; forebrain regionalization [GO:0021871]; GPI anchor metabolic process [GO:0006505]; positive regulation of ER to Golgi vesicle-mediated transport [GO:1902953]; protein transport [GO:0015031]; sensory perception of sound [GO:0007605]
P52888	THOP1	Thimet oligopeptidase OS	78.8	0.01	-6.64	1.91E-16	15.72	peptide metabolic process [GO:0006518]; protein polyubiquitination [GO:0000209]; proteolysis [GO:0006508]	cytosol [GO:0005829]; mitochondrial intermembrane space [GO:0005758]; metal ion binding [GO:0046872]; metalloendopeptidase activity [GO:0004222]; peptide metabolic process

									[GO:0006518]; protein polyubiquitination [GO:0000209]; proteolysis [GO:0006508]
P37268	FDFT1	Squalene synthase OS	48.1	0.01	-6.64	1.91E-16	15.72	cholesterol biosynthetic process [GO:0006695]; farnesyl diphosphate metabolic process [GO:0045338]; steroid biosynthetic process [GO:0006694]	endoplasmic reticulum [GO:0005783]; endoplasmic reticulum membrane [GO:0005789]; integral component of membrane [GO:0016021]; farnesyl-diphosphate farnesyltransferase activity [GO:0004310]; metal ion binding [GO:0046872]; squalene synthase activity [GO:0051996]; cholesterol biosynthetic process [GO:0006695]; farnesyl diphosphate metabolic process [GO:0045338]; steroid biosynthetic process [GO:0006694]
Q9H4A4	RNPEP	Aminopeptidase B OS	72.5	0.01	-6.64	1.91E-16	15.72	proteolysis [GO:0006508]	extracellular exosome [GO:0070062]; extracellular region [GO:0005576]; extracellular space [GO:0005615]; plasma membrane [GO:0005886]; aminopeptidase activity [GO:0004177]; epoxide hydrolase activity [GO:0004301]; metalloaminopeptidase activity [GO:0070006]; metalloexopeptidase activity [GO:0008235]; zinc ion binding [GO:0008270]; proteolysis [GO:0006508]
P58335	ANTXR2	Anthrax toxin receptor 2 OS	53.6	0.01	-6.64	1.91E-16	15.72	toxin transport [GO:1901998]	cell surface [GO:0009986]; endoplasmic reticulum membrane [GO:0005789]; endosome membrane [GO:0010008]; extracellular region [GO:0005576]; integral component of membrane [GO:0016021]; plasma membrane [GO:0005886]; metal ion binding [GO:0046872]; transmembrane signaling receptor activity [GO:0004888]; toxin transport [GO:1901998]
Q96FN4	CPNE2	Copine-2 OS	61.2	0.01	-6.64	1.91E-16	15.72	cellular response to calcium ion [GO:0071277]	cytoplasm [GO:0005737]; extracellular exosome [GO:0070062]; nucleus [GO:0005634]; plasma membrane [GO:0005886]; calcium-dependent phospholipid binding [GO:0005544]; metal ion binding [GO:0046872]; cellular response to calcium ion [GO:0071277]
Q86Y56	DNAAF5	Dynein axonemal assembly factor 5 OS	93.5	0.01	-6.64	1.91E-16	15.72	cilium movement [GO:0003341]; inner dynein arm assembly [GO:0036159]; outer dynein arm assembly [GO:0036158]	cytoplasm [GO:0005737]; dynein axonemal particle [GO:0120293]; dynein intermediate chain binding [GO:0045505]; cilium movement [GO:0003341]; inner dynein arm assembly [GO:0036159]; outer dynein arm assembly [GO:0036158]

Q9NR46	SH3GLB2	Endophilin-B2 OS	43.9	0.01	-6.64	1.91E-16	15.72	membrane organization [GO:0061024]	cytoplasm [GO:0005737]; cytosol [GO:0005829]; membrane [GO:0016020]; nucleoplasm [GO:0005654]; cadherin binding [GO:0045296]; identical protein binding [GO:0042802]; membrane organization [GO:0061024]
Q96CU9	FOXRED1	FAD-dependent oxidoreductase domain-containing protein 1 OS	53.8	0.01	-6.64	1.91E-16	15.72	mitochondrial respiratory chain complex I assembly [GO:0032981]	cytoplasm [GO:0005737]; mitochondrial inner membrane [GO:0005743]; mitochondrial respiratory chain complex I [GO:0005747]; mitochondrion [GO:0005739]; oxidoreductase activity [GO:0016491]; mitochondrial respiratory chain complex I assembly [GO:0032981]
P0CG08	GPR89B	Golgi pH regulator B OS	52.9	0.01	-6.64	1.91E-16	15.72	intracellular pH reduction [GO:0051452]; protein transport [GO:0015031]; regulation of ion transmembrane transport [GO:0034765]; T cell differentiation [GO:0030217]	Golgi cisterna membrane [GO:0032580]; Golgi membrane [GO:0000139]; integral component of membrane [GO:0016021]; voltage-gated anion channel activity [GO:0008308]; intracellular pH reduction [GO:0051452]; protein transport [GO:0015031]; regulation of ion transmembrane transport [GO:0034765]; T cell differentiation [GO:0030217]
Q8WVV9	HNRNPLL	Heterogeneous nuclear ribonucleoprotein L-like OS	60	0.01	-6.64	1.91E-16	15.72	mRNA processing [GO:0006397]; positive regulation of RNA splicing [GO:0033120]; regulation of RNA splicing [GO:0043484]	membrane [GO:0016020]; nucleus [GO:0005634]; mRNA binding [GO:0003729]; RNA binding [GO:0003723]; mRNA processing [GO:0006397]; positive regulation of RNA splicing [GO:0033120]; regulation of RNA splicing [GO:0043484]
Q9H089	LSG1	Large subunit GTPase 1 homolog OS	75.2	0.01	-6.64	1.91E-16	15.72	nuclear export [GO:0051168]; protein transport [GO:0015031]; ribosomal subunit export from nucleus [GO:0000054]	Cajal body [GO:0015030]; cytosol [GO:0005829]; endoplasmic reticulum [GO:0005783]; membrane [GO:0016020]; nuclear body [GO:0016604]; nucleoplasm [GO:0005654]; GTP binding [GO:0005525]; GTPase activity [GO:0003924]; nuclear export [GO:0051168]; protein transport [GO:0015031]; ribosomal subunit export from nucleus [GO:0000054]
Q9H6V9	LDAH	Lipid droplet-associated hydrolase OS	37.3	0.01	-6.64	1.91E-16	15.72	lipid catabolic process [GO:0016042]; lipid storage [GO:0019915]	endoplasmic reticulum [GO:0005783]; lipid droplet [GO:0005811]; sterol esterase activity [GO:0004771]; lipid catabolic process [GO:0016042]; lipid storage [GO:0019915]

Q9NWS0	PIH1D1	PIH1 domain-containing protein 1 OS	32.3	0.01	-6.64	1.91E-16	15.72	box C/D snoRNP assembly [GO:0000492]; chromatin remodeling [GO:0006338]; epithelial cell differentiation [GO:0030855]; establishment of protein localization to chromatin [GO:0071169]; negative regulation of cysteine-type endopeptidase activity involved in apoptotic signaling pathway [GO:2001268]; negative regulation of histone H3-K9 dimethylation [GO:1900110]; negative regulation of histone H3-K9 trimethylation [GO:1900113]; negative regulation of histone H4-K16 acetylation [GO:2000619]; positive regulation of glucose mediated signaling pathway [GO:1902661]; positive regulation of histone H3-K9 acetylation [GO:2000617]; positive regulation of histone H4 acetylation [GO:0090240]; positive regulation of protein serine/threonine kinase activity [GO:0071902]; positive regulation of protein-containing complex assembly [GO:0031334]; positive regulation of TORC1 signaling [GO:1904263]; positive regulation of transcription of nucleolar large rRNA by RNA polymerase I [GO:1901838]; protein stabilization [GO:0050821]; regulation of histone H3-K4 methylation [GO:0051569]; rRNA processing [GO:0006364]; snoRNA localization [GO:0048254]; TORC1 complex assembly [GO:1905669]	cytoplasm [GO:0005737]; nucleolus [GO:0005730]; nucleus [GO:0005634]; pre-snoRNP complex [GO:0070761]; R2TP complex [GO:0097255]; ribonucleoprotein complex [GO:1990904]; RPAP3/R2TP/prefoldin-like complex [GO:1990062]; ATPase binding [GO:0051117]; histone binding [GO:0042393]; phosphoprotein binding [GO:0051219]; protein kinase binding [GO:0019901]; RNA polymerase I core promoter sequence-specific DNA binding [GO:0001164]; box C/D snoRNP assembly [GO:0000492]; chromatin remodeling [GO:0006338]; epithelial cell differentiation [GO:0030855]; establishment of protein localization to chromatin [GO:0071169]; negative regulation of cysteine-type endopeptidase activity involved in apoptotic signaling pathway [GO:2001268]; negative regulation of histone H3-K9 dimethylation [GO:1900110]; negative regulation of histone H3-K9 trimethylation [GO:1900113]; negative regulation of histone H4-K16 acetylation [GO:2000619]; positive regulation of glucose mediated signaling pathway [GO:1902661]; positive regulation of histone H3-K9 acetylation [GO:2000617]; positive regulation of histone H4 acetylation [GO:0090240]; positive regulation of protein serine/threonine kinase activity [GO:0071902]; positive regulation of protein-containing complex assembly [GO:0031334]; positive regulation of TORC1 signaling [GO:1904263]; positive regulation of transcription of nucleolar large rRNA by RNA polymerase I [GO:1901838]; protein stabilization [GO:0050821]; regulation of histone H3-K4 methylation [GO:0051569]; rRNA processing [GO:0006364]; snoRNA localization [GO:0048254]; TORC1 complex assembly [GO:1905669]
Q9UHA3	RSL24D1	Probable ribosome biogenesis protein RLP24 OS	19.6	0.01	-6.64	1.91E-16	15.72	ribosomal large subunit biogenesis [GO:0042273]; translation [GO:0006412]	nucleolus [GO:0005730]; nucleoplasm [GO:0005654]; ribosome [GO:0005840]; structural constituent of ribosome [GO:0003735]; ribosomal large subunit biogenesis [GO:0042273]; translation [GO:0006412]
P98173	FAM3A	Protein FAM3A OS	25.1	0.01	-6.64	1.91E-16	15.72	antifungal humoral response [GO:0019732]; antimicrobial humoral immune response	extracellular space [GO:0005615]; antifungal humoral response [GO:0019732]; antimicrobial

								mediated by antimicrobial peptide [GO:0061844]; negative regulation of antifungal innate immune response [GO:1905035]; negative regulation of gluconeogenesis [GO:0045721]; regulation of lipid biosynthetic process [GO:0046890]	humoral immune response mediated by antimicrobial peptide [GO:0061844]; negative regulation of antifungal innate immune response [GO:1905035]; negative regulation of gluconeogenesis [GO:0045721]; regulation of lipid biosynthetic process [GO:0046890]
Q08174	PCDH1	Protocadherin-1 OS	114.7	0.01	-6.64	1.91E-16	15.72	cell adhesion [GO:0007155]; cell-cell signaling [GO:0007267]; homophilic cell adhesion via plasma membrane adhesion molecules [GO:0007156]; nervous system development [GO:0007399]	cell junction [GO:0030054]; cell-cell junction [GO:0005911]; integral component of plasma membrane [GO:0005887]; intracellular membrane-bounded organelle [GO:0043231]; nucleolus [GO:0005730]; nucleoplasm [GO:0005654]; plasma membrane [GO:0005886]; calcium ion binding [GO:0005509]; cell adhesion [GO:0007155]; cell-cell signaling [GO:0007267]; homophilic cell adhesion via plasma membrane adhesion molecules [GO:0007156]; nervous system development [GO:0007399]
A8MWX3	WASH4P	Putative WAS protein family homolog 4 OS	51.6	0.01	-6.64	1.91E-16	15.72	Arp2/3 complex-mediated actin nucleation [GO:0034314]; endocytic recycling [GO:0032456]; endosomal transport [GO:0016197]; exocytosis [GO:0006887]; regulation of Arp2/3 complex-mediated actin nucleation [GO:0034315]; retrograde transport, endosome to Golgi [GO:0042147]	cytosol [GO:0005829]; early endosome [GO:0005769]; early endosome membrane [GO:0031901]; recycling endosome [GO:0055037]; recycling endosome membrane [GO:0055038]; WASH complex [GO:0071203]; actin binding [GO:0003779]; alpha-tubulin binding [GO:0043014]; gamma-tubulin binding [GO:0043015]; Arp2/3 complex-mediated actin nucleation [GO:0034314]; endocytic recycling [GO:0032456]; endosomal transport [GO:0016197]; exocytosis [GO:0006887]; regulation of Arp2/3 complex-mediated actin nucleation [GO:0034315]; retrograde transport, endosome to Golgi [GO:0042147]
Q96HR9	REEP6	Receptor expression-enhancing protein 6 OS	23.4	0.01	-6.64	1.91E-16	15.72	detection of light stimulus involved in visual perception [GO:0050908]; endoplasmic reticulum organization [GO:0007029]; regulation of intracellular transport [GO:0032386]	clathrin-coated vesicle membrane [GO:0030665]; endoplasmic reticulum [GO:0005783]; endoplasmic reticulum membrane [GO:0005789]; integral component of membrane [GO:0016021]; nucleus [GO:0005634]; photoreceptor inner segment [GO:0001917]; detection of light stimulus involved in visual perception [GO:0050908]; endoplasmic reticulum organization [GO:0007029]; regulation of intracellular transport [GO:0032386]

Q8TD22	SFXN5	Sideroflexin-5 OS	37.1	0.01	-6.64	1.91E-16	15.72	amino acid transport [GO:0006865]; citrate transport [GO:0015746]; mitochondrial transmembrane transport [GO:1990542]	integral component of mitochondrial inner membrane [GO:0031305]; mitochondrion [GO:0005739]; citrate transmembrane transporter activity [GO:0015137]; transmembrane transporter activity [GO:0022857]; amino acid transport [GO:0006865]; citrate transport [GO:0015746]; mitochondrial transmembrane transport [GO:1990542]
O60635	TSPAN1	Tetraspanin-1 OS	26.3	0.01	-6.64	1.91E-16	15.72	protein stabilization [GO:0050821]	cell junction [GO:0030054]; cytoplasm [GO:0005737]; extracellular exosome [GO:0070062]; integral component of plasma membrane [GO:0005887]; intracellular membrane-bounded organelle [GO:0043231]; lysosomal membrane [GO:0005765]; membrane [GO:0016020]; nucleoplasm [GO:0005654]; perinuclear region of cytoplasm [GO:0048471]; plasma membrane [GO:0005886]; vesicle [GO:0031982]; protein stabilization [GO:0050821]
Q9H4I3	TRABD	TraB domain-containing protein OS	42.3	0.01	-6.64	1.91E-16	15.72		
Q8IWB7	WDFY1	WD repeat and FYVE domain-containing protein 1 OS	46.3	0.01	-6.64	1.91E-16	15.72	positive regulation of toll-like receptor 3 signaling pathway [GO:0034141]; positive regulation of toll-like receptor 4 signaling pathway [GO:0034145]	cell junction [GO:0030054]; cytosol [GO:0005829]; early endosome [GO:0005769]; Golgi apparatus [GO:0005794]; intracellular membrane-bounded organelle [GO:0043231]; nucleolus [GO:0005730]; nucleus [GO:0005634]; 1-phosphatidylinositol binding [GO:0005545]; zinc ion binding [GO:0008270]; positive regulation of toll-like receptor 3 signaling pathway [GO:0034141]; positive regulation of toll-like receptor 4 signaling pathway [GO:0034145]
Q8WU90	ZC3H15	Zinc finger CCCH domain-containing protein 15 OS	48.6	0.01	-6.64	1.91E-16	15.72	cytokine-mediated signaling pathway [GO:0019221]; cytoplasmic translation [GO:0002181]; positive regulation of GTPase activity [GO:0043547]	cytosol [GO:0005829]; nucleus [GO:0005634]; cadherin binding [GO:0045296]; metal ion binding [GO:0046872]; RNA binding [GO:0003723]; cytokine-mediated signaling pathway [GO:0019221]; cytoplasmic translation [GO:0002181]; positive regulation of GTPase activity [GO:0043547]

Table S1. Differentially expressed proteins in ANXA1 KO *versus* WT.

This table reports around 130 significantly over expressed proteins (ratio > 2-fold, p-value< 0.1) and around significantly down expressed 90 proteins (ratio < 0.5-fold p-value< 0.1) in ANXA1 KO samples *vs* WT.
Each protein is reported with the following information: UniProt Accession, gene symbol, protein name (i.e., description), molecular weight (i.e., MW, in kDa) and the label-free quantification results expressed as fold changes of ANXA1 KO *vs* WT (and their corresponding Log_2) and the relative p-values (also reported as their minus Log_{10} values). Furthermore, a GO-term analysis for both GO and biological processes is reported, highlighting in light red all the terms related to apoptosis and cell death and in green all the terms related to mitosis and its spindle.
