# Supplementary material for: Role of Intracellular and Extracellular Annexin A1 in MIA PaCa-2 Spheroids Formation and Drug Sensitivity
Source: Cancers (Basel). 2022 Sep 29;14(19):4764. doi: 10.3390/cancers14194764 (PMC9563593; doi:10.3390/cancers14194764)
Supplement: Supplementary file 1 [file cancers-14-04764-s001.zip › Table S2.pdf]

Category	Term	Count	%	P-Value	Genes	Fold Enrichment
UP	GO:0008544~epidermis development	14	10.9375	9.16E-07	P31944, P01040, P49862, Q86YZ3, Q15517, P23490, Q5T750, P15924, Q08188, Q01469, Q9NZT1, P20930, P22735, Q5D862	5.309356725
UP	GO:0043588~skin development	12	9.375	9.76E-06	P02452, P20930, P22735, P31944, P01040, Q86YZ3, Q15517, P23490, Q5D862, P15924, P14923, Q08188	5.188
UP	GO:0030216~keratinocyte differentiation	9	7.03125	4.27E-06	P20930, P22735, P31944, P01040, Q86YZ3, Q15517, P23490, P15924, Q08188	8.458695652
UP	GO:0009913~epidermal cell differentiation	9	7.03125	1.03E-04	P20930, P22735, P31944, P01040, Q86YZ3, Q15517, P23490, P15924, Q08188	5.722058824
UP	GO:0044057~regulation of system process	9	7.03125	0.006534	Q01469, Q12797, P07900, Q99996, P15924, P14923, P42892, P19971, P12883	3.137903226
UP	GO:0031589~cell-substrate adhesion	9	7.03125	0.007203	P02452, P18206, P06756, P12814, P14923, Q15262, P32004, O75044, P02751	3.088095238
UP	GO:0003012~muscle system process	9	7.03125	0.007923	P35749, Q12797, Q6ZMU5, P07900, Q99996, P15924, P14923, Q8WZ42, P12883	3.03984375
UP	GO:0006936~muscle contraction	8	6.25	0.006924	P35749, Q12797, P07900, Q99996, P15924, P14923, Q8WZ42, P12883	3.458666667
UP	GO:0010951~negative regulation of endopeptidase activity	8	6.25	0.008597	P19823, Q8IW75, P01040, P48594, P01009, Q96P63, P02788, P01023	3.325641026
UP	GO:0010466~negative regulation of peptidase activity	8	6.25	0.012808	P19823, Q8IW75, P01040, P48594, P01009, Q96P63, P02788, P01023	3.088095238
UP	GO:0034330~cell junction organization	8	6.25	0.01406	P18206, Q13835, Q02413, P12814, P15924, P14923, Q15262, P02751	3.033918129
UP	GO:0018149~peptide cross-linking	7	5.46875	1.49E-06	P20930, P22735, P01040, P23490, P15924, P02751, Q08188	15.13166667
UP	GO:0060048~cardiac muscle contraction	7	5.46875	0.001101	Q12797, P07900, Q99996, P15924, P14923, Q8WZ42, P12883	5.604320988
UP	GO:0006941~striated muscle contraction	7	5.46875	0.002355	Q12797, P07900, Q99996, P15924, P14923, Q8WZ42, P12883	4.881182796
UP	GO:1903522~regulation of blood circulation	7	5.46875	0.004467	Q12797, P07900, Q99996, P15924, P14923, P42892, P12883	4.323333333
UP	GO:0060047~heart contraction	7	5.46875	0.004467	Q12797, P07900, Q99996, P15924, P14923, Q8WZ42, P12883	4.323333333
UP	GO:0003015~heart process	7	5.46875	0.00773	Q12797, P07900, Q99996, P15924, P14923, Q8WZ42, P12883	3.87991453
UP	GO:0007160~cell-matrix adhesion	7	5.46875	0.008761	P18206, P06756, P12814, P14923, Q15262, P32004, P02751	3.782916667
UP	GO:0034329~cell junction assembly	7	5.46875	0.022829	P18206, Q13835, Q02413, P12814, P14923, Q15262, P02751	3.088095238

UP	GO:0045216~cell-cell junction organization	7	5.46875	0.024998	P18206, Q13835, Q02413, P12814, P15924, P14923, Q15262	3.026333333
UP	GO:0031424~keratinization	6	4.6875	1.08E-06	P22735, P31944, Q86YZ3, Q15517, P23490, Q08188	21.61666667
UP	GO:0006942~regulation of striated muscle contraction	6	4.6875	0.001294	Q12797, P07900, Q99996, P15924, P14923, P12883	6.826315789
UP	GO:0006937~regulation of muscle contraction	6	4.6875	0.005657	Q12797, P07900, Q99996, P15924, P14923, P12883	4.988461538
UP	GO:0008016~regulation of heart contraction	6	4.6875	0.00915	Q12797, P07900, Q99996, P15924, P14923, P12883	4.472413793
UP	GO:0002831~regulation of response to biotic stimulus	6	4.6875	0.015801	P07900, P49862, P27361, P02788, Q9NZM5, P05089	3.93030303
UP	GO:0090257~regulation of muscle system process	6	4.6875	0.017862	Q12797, P07900, Q99996, P15924, P14923, P12883	3.814705882
UP	GO:0002449~lymphocyte mediated immunity	6	4.6875	0.037324	P48594, P05089, P25311, P01893, P04003, P01782	3.163414634
UP	GO:0055117~regulation of cardiac muscle contraction	5	3.90625	0.006297	Q12797, P07900, Q99996, P15924, P14923	6.357843137
UP	GO:1990138~neuron projection extension	5	3.90625	0.016212	P18206, P07900, Q96F07, P32004, P02751	4.912878788
UP	GO:0060560~developmental growth involved in morphogenesis	5	3.90625	0.025225	P18206, P07900, Q96F07, P32004, P02751	4.323333333
UP	GO:0002706~regulation of lymphocyte mediated immunity	5	3.90625	0.028783	P48594, P05089, P25311, P01893, P04003	4.157051282
UP	GO:0048588~developmental cell growth	5	3.90625	0.041169	P18206, P07900, Q96F07, P32004, P02751	3.727011494
UP	GO:0050891~multicellular organismal water homeostasis	4	3.125	0.002878	P20930, P15121, Q86YZ3, Q5D862	12.35238095
UP	GO:0030104~water homeostasis	4	3.125	0.004452	P20930, P15121, Q86YZ3, Q5D862	10.80833333
UP	GO:0014065~phosphatidylinositol 3-kinase signaling	4	3.125	0.011855	Q8IW75, Q8IY47, Q9NZM5, P02751	7.860606061
UP	GO:0048015~phosphatidylinositol-mediated signaling	4	3.125	0.023652	Q8IW75, Q8IY47, Q9NZM5, P02751	6.176190476
UP	GO:0001910~regulation of leukocyte mediated cytotoxicity	4	3.125	0.023652	P48594, P05089, P25311, P01893	6.176190476
UP	GO:0048017~inositol lipid-mediated signaling	4	3.125	0.023652	Q8IW75, Q8IY47, Q9NZM5, P02751	6.176190476

UP	GO:0072376~protein activation cascade	4	3.125	0.028595	P02751, P01023, P04003, P01782	5.764444444
UP	GO:0002027~regulation of heart rate	4	3.125	0.028595	Q99996, P15924, P14923, P12883	5.764444444
UP	GO:0031341~regulation of cell killing	4	3.125	0.034043	P48594, P05089, P25311, P01893	5.404166667
UP	GO:0060135~maternal process involved in female pregnancy	4	3.125	0.034043	P15121, Q02413, P27361, P05089	5.404166667
UP	GO:0048675~axon extension	4	3.125	0.046423	P18206, P07900, P32004, P02751	4.803703704
UP	GO:0045229~external encapsulating structure organization	3	2.34375	0.006074	P22735, Q86YZ3, Q08188	21.61666667
UP	GO:0043163~cell envelope organization	3	2.34375	0.006074	P22735, Q86YZ3, Q08188	21.61666667
UP	GO:0090136~epithelial cell-cell adhesion	3	2.34375	0.011785	P18206, P15924, P14923	16.2125
UP	GO:0061436~establishment of skin barrier	3	2.34375	0.011785	P20930, Q86YZ3, Q5D862	16.2125
UP	GO:0033561~regulation of water loss via skin	3	2.34375	0.019054	P20930, Q86YZ3, Q5D862	12.97
UP	GO:0045109~intermediate filament organization	3	2.34375	0.027731	Q13835, P15924, P07197	10.80833333
UP	GO:0002920~regulation of humoral immune response	3	2.34375	0.037672	P49862, P01023, P04003	9.264285714
UP	GO:0002707~negative regulation of lymphocyte mediated immunity	3	2.34375	0.048745	P48594, P05089, P04003	8.10625
UP	GO:0055008~cardiac muscle tissue morphogenesis	3	2.34375	0.048745	P15924, Q8WZ42, P12883	8.10625
UP	GO:0086091~regulation of heart rate by cardiac conduction	3	2.34375	0.048745	Q99996, P15924, P14923	8.10625
UP	GO:0060324~face development	3	2.34375	0.048745	P02452, Q12797, P27361	8.10625
DOWN	GO:0071902~positive regulation of protein serine/threonine kinase activity	5	5.617978	0.040026	Q13443, P14635, Q9NWS0, P09038, Q9NQS7	3.770348837
DOWN	GO:1901976~regulation of cell cycle checkpoint	4	4.494382	0.022516	Q13769, Q9NXR7, P14635, Q9NQS7	6.3500612

DOWN	GO:0007080~mitotic metaphase plate congression	4	4.494382	0.041837	Q99661, P14635, Q9BW19, Q9NQS7	5.027131783
DOWN	GO:0051310~metaphase plate congression	4	4.494382	0.041837	Q99661, P14635, Q9BW19, Q9NQS7	5.027131783
DOWN	GO:0051988~regulation of attachment of spindle microtubules to kinetochore	3	3.370787	0.026134	Q9H0H5, P14635, Q9NQS7	11.31104651

Table S2. GO enrichment analysis of ANXA1 KO and WT up- and down-regulated proteins.

This table reports the GO enrichment analysis for the *Biological Process* terms for both ANXA1 KO up- and down-regulated proteins.
The following data are reported: the category for which an enriched term was obtained (i.e., from up- or down-regulated proteins), the enriched term, the count (i.e., the number of proteins, defined by DAVID as genes, responsible for the enrichment), an enrichment percentage, the P-value correlated to the enriched terms, the genes (i.e., the UniProt accession numbers) and the fold of the enrichment. The showed terms were retrieved filtering out those whose P-Value was > 0.05 and whose Fold enrichment was < 3.
