# Supplementary material for: Role of Intracellular and Extracellular Annexin A1 in MIA PaCa-2 Spheroids Formation and Drug Sensitivity
Source: Cancers (Basel). 2022 Sep 29;14(19):4764. doi: 10.3390/cancers14194764 (PMC9563593; doi:10.3390/cancers14194764)
Supplement: Supplementary file 1 [file cancers-14-04764-s001.zip › Table S3.pdf]

Category	Term	Count	%	P-Value	Genes	Fold Enrichment
UP	KW-0130~Cell adhesion	11	8.59375	3.37E-05	P18206, P29322, P06756, P01040, Q13835, Q02413, P14923, Q96F07, P32004, Q08554, P02751	4.978602
UP	KW-0417~Keratinization	4	3.125	2.74E-04	P22735, Q86YZ3, P23490, Q08188	23.53521
DOWN	KW-0498~Mitosis	7	7.865169	0.022644	Q86WB0, Q99661, Q9NXR7, P14635, Q9NVM9, Q9BW19, Q9NQS7	3.046296

Table S3. Functional annotation enrichment analysis of ANXA1 KO and WT up- and down-regulated proteins.

This table reports the Functional annotation enrichment analysis for the *Biological Process keywords* terms for both ANXA1 KO and WT up- and down-regulated proteins. The following data are reported: the category for which an enriched term was obtained (i.e., from up- or down-regulated proteins), the enriched term, the count (i.e., the number of proteins, defined by DAVID as genes, responsible for the enrichment), an enrichment percentage, the P-value correlated to the enriched terms, the genes (i.e., the UniProt accession numbers) and the fold of the enrichment. The showed terms were retrieved filtering out those whose P-Value was > 0.05 and whose Fold enrichment was < 3.
