# Supplementary figures and images for: Role of Intracellular and Extracellular Annexin A1 in MIA PaCa-2 Spheroids Formation and Drug Sensitivity
Source: Cancers (Basel). 2022 Sep 29;14(19):4764. doi: 10.3390/cancers14194764 (PMC9563593; doi:10.3390/cancers14194764)

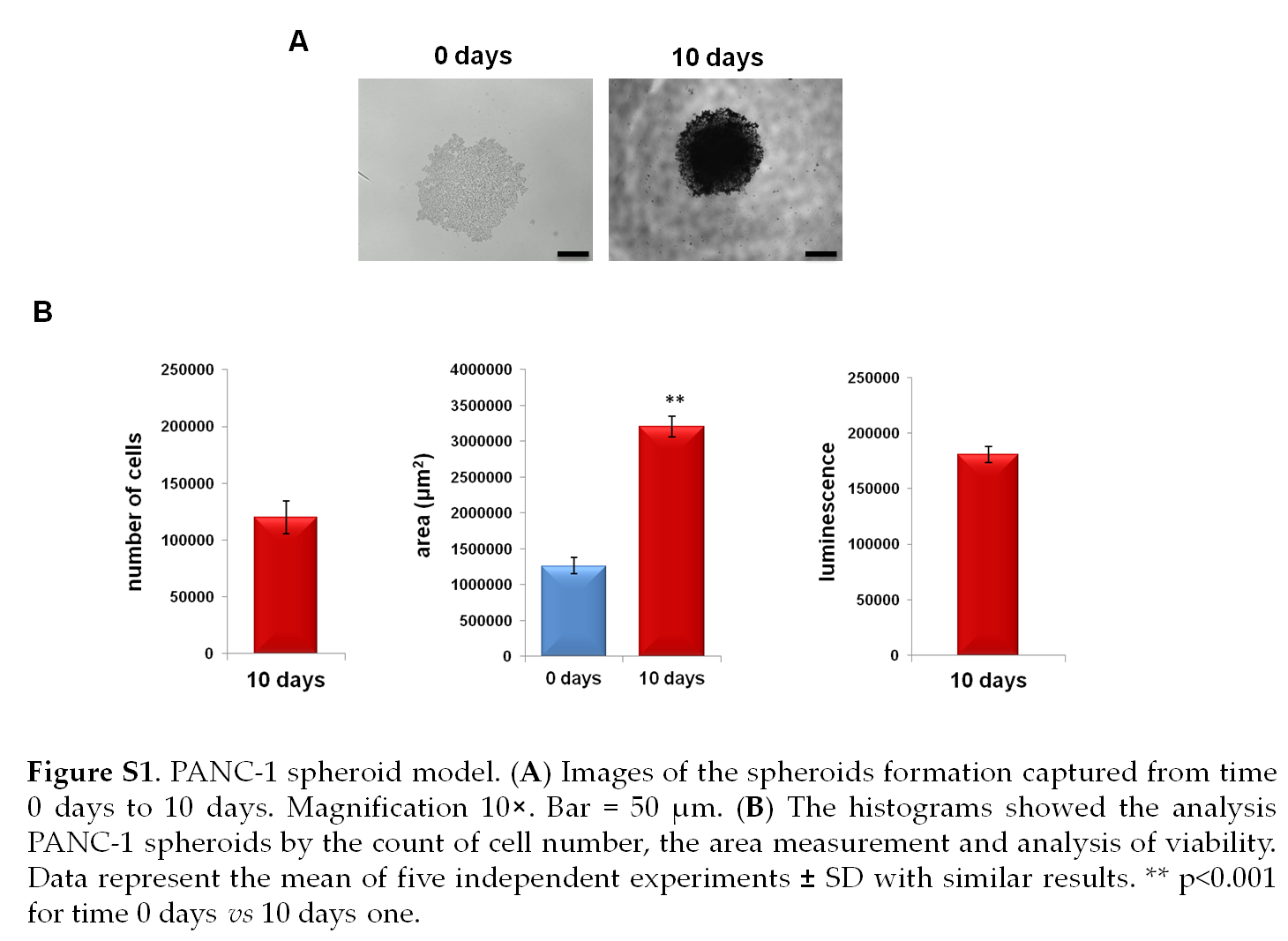

Supplement: Supplementary file 1 [file cancers-14-04764-s001.zip › figure S1.tif]

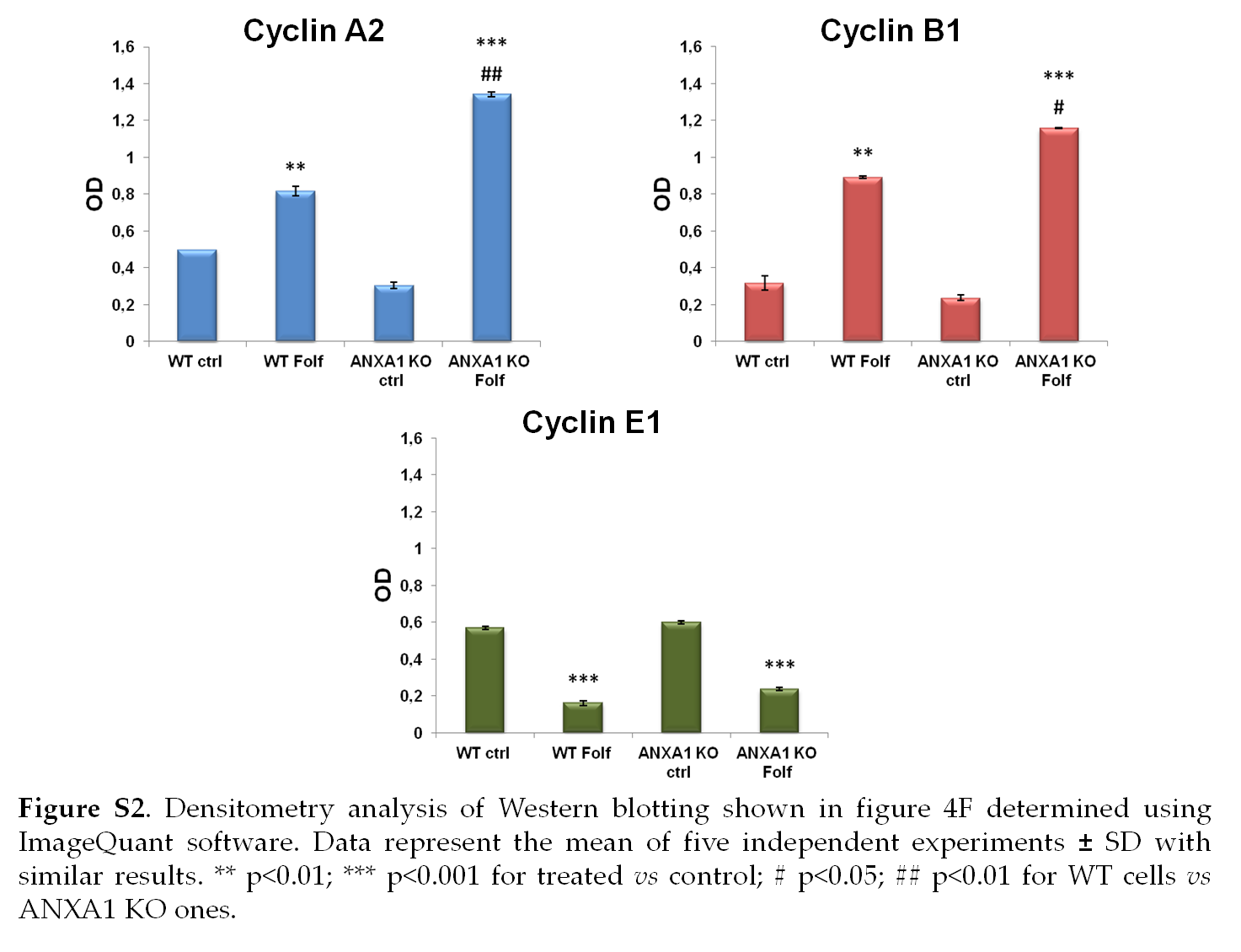

Supplement: Supplementary file 1 [file cancers-14-04764-s001.zip › figure S2.tif]

Cyclin A2

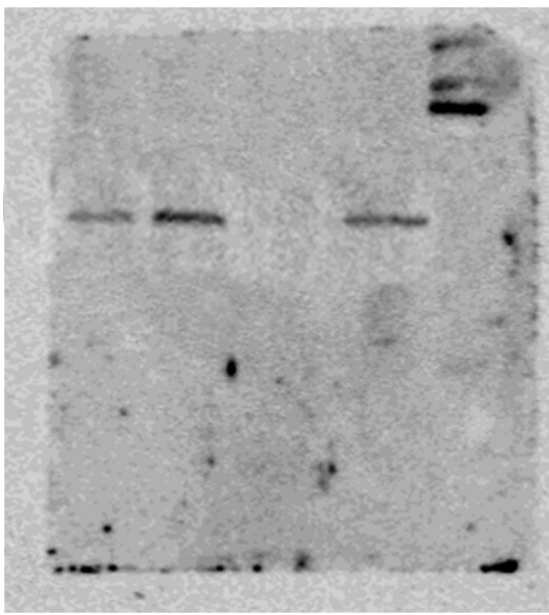

Cyclin E1

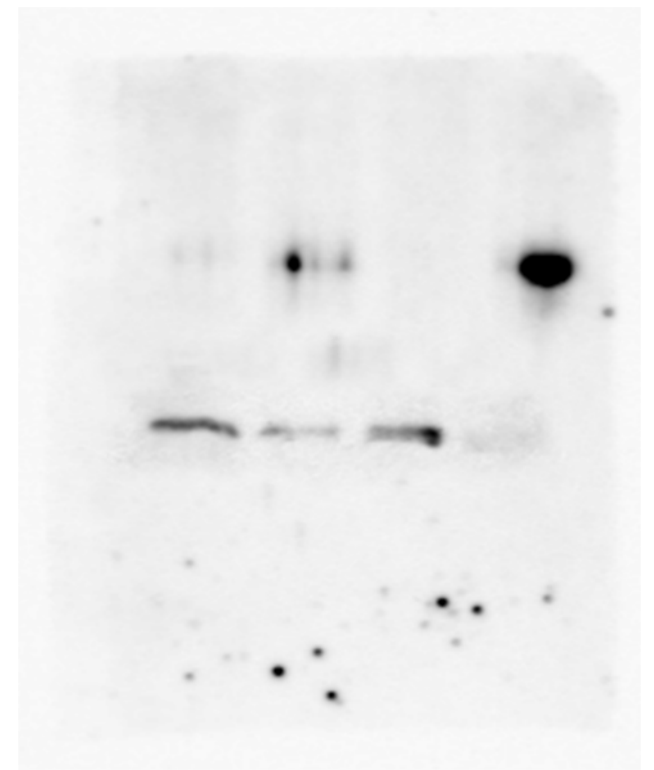

Cyclin B1

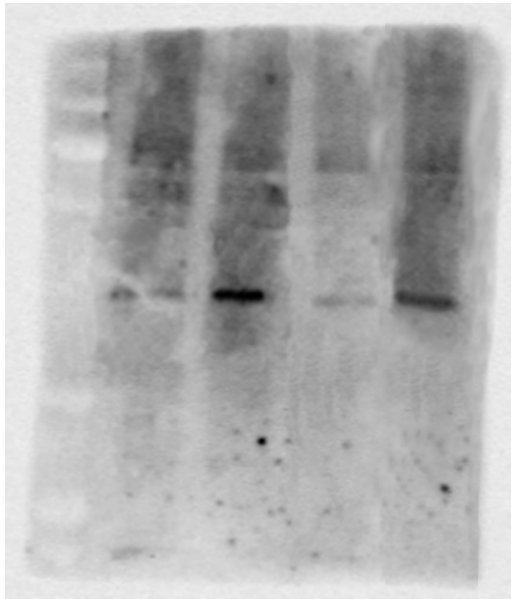

GAPDH

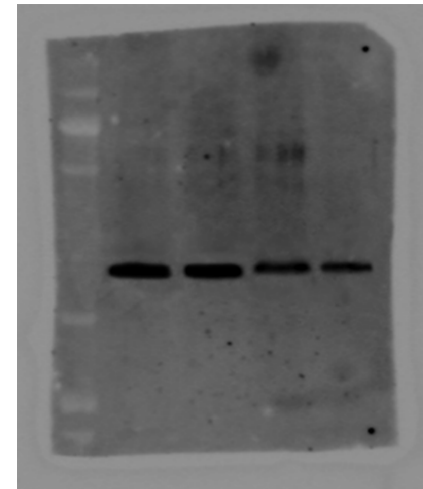

Supplement: Supplementary file 1 [file cancers-14-04764-s001.zip › File S1.pdf]
